# Plant phenotypic differentiation outweighs genetic variation in shaping the lettuce leaf microbiota

**DOI:** 10.1186/s40793-026-00850-6

**Published:** 2026-01-30

**Authors:** Arianna Capparotto, Guillaume Chesneau, Alessandra Tondello, Esteban Orellana, Piergiorgio Stevanato, Tiziano Bonato, Andrea Squartini, Stéphane Hacquard, Marco Giovannetti

**Affiliations:** 1 Department of Biology, University of Padova, 35131 Padua, Italy; 2 Department of Life Sciences and Systems Biology, University of Torino, 10125 Turin, Italy; 3 Department of Plant-Microbe Interactions, Max Planck Institute for Plant Breeding Research, 50829 Cologne, Germany; 4 Department of Agronomy, Food, Natural Resources, Animals and Environment, DAFNAE, University of Padova, 35020 Legnaro, Italy; 5 Department of Environmental and Resource Engineering Waste, Climate & Monitoring, Technical University of Denmark (DTU), 2800 Kongens Lyngby, Denmark; 6 Società Estense Servizi Ambientali (S.E.S.A. S.p.A.), 35042 Este, Italy; 7 Department of Environmental Sciences, Informatics, and Statistics, Ca’ Foscari University Venice, Via Torino 155, 30172 Venezia-Mestre, Italy

**Keywords:** Lettuce, Leaf microbiome, 16S metabarcoding, Leaf phenotypic traits, Edible microbiome, Leaf ionome

## Abstract

Lettuce, a widely consumed raw vegetable, harbors leaf-associated microbial communities whose understanding and prediction are crucial for plant and human health. While environmental factors are known to strongly influence plant leaf microbiomes, the role of plant-specific determinants in shaping microbial diversity remains unclear. In this study, we investigated how three key plant factors -genetic distance, plant variety and leaf micro- and macronutrient content- influence the composition and diversity of lettuce leaf bacterial communities, by analyzing 131 fully-sequenced *Lactuca sativa* genotypes via 16S rRNA amplicon sequencing. Our findings revealed that variety, as defined by breeders, exerts a greater influence on bacterial community diversity than genetic distance or variations in leaf nutrient levels. Together with available and detailed shoot traits they explained 13.4% of the observed bacterial diversity. Inspection of 9 specific leaf morphological traits, with further validation by MAGs analysis, showed that heart formation, head height, and venation types significantly shaped bacterial richness and evenness, mainly acting on non-hub members. These results highlight the strong relationship between leaf morphology and bacterial community structure, suggesting that phenotypic traits play an outsized but understudied role in shaping the leaf microbiota, a crucial aspect of the edible microbiome.

**Supplementary Information:**

The online version contains supplementary material available at 10.1186/s40793-026-00850-6.

## Introduction

Lettuce (*Lactuca sativa*), a leafy vegetable from the Asteraceae family, is one of the most widely consumed green vegetables, holding a significant position in global agriculture [1]. The widespread consumption of lettuce can be largely attributed to its favorable nutritional profile: its leaves are low in calories and fats while being rich in fiber, vitamins, and essential minerals such as iron [2]. In addition to this, emerging evidence highlights the beneficial aspects of the “edible microbiome” associated with lettuce leaves [3, 4]. For example, 31 bacterial genera (such as Pseudomonas, Sphingomonas, Aeromicrobium, and Pantoea) present in fruits and vegetables, including lettuce, have been shown to be shared with the human gut microbiome and to affect its diversity [5]. Additionally, epiphytic lactic acid bacteria found on the surface of lettuce leaves can survive digestion and persist in the gut, positively influencing its microbial composition [6]. Given this evidence, advancing our understanding of the factors that shape the bacterial community on lettuce leaves could foster beneficial microbial populations that are important for both human nutrition and plant health, ultimately reducing resource losses.

While environmental factors, such as air temperature, humidity, solar radiation, wind, geographic location and local neighborhood identity account for most of the variation in leaf bacterial communities [7–9], emerging evidence also points to the impact of intrinsic plant determinants, such as genotypes, leaf morphology and secretion of secondary metabolites, as secondary but important contributors to this variation [10–12]. In lettuce, the impact of genotype has been demonstrated for fungi [13] and for culturable bacteria-associated communities [14, 15]. Recently, genome-wide association (GWA) has been employed to associate host genetic variation with phyllosphere microbiota differentiation, identifying genes potentially associated with microbiome variation [16]. However, the extent to which plant genetic variability shapes the assembly of microbial communities remains poorly understood, partly due to the limited availability of comprehensive leaf microbiota studies on non-model plants. In addition, increasing evidence supports the role of plant morphological and physiological traits in driving microbial community composition. For example, small-scale studies on lettuce have shown that macroscopic [14], as well as microscopic features like stomatal density, epidermal cell patterning, and cuticle hydrophobicity [17], create preferential attachment sites for fungi or bacteria. Regarding leaf nutrient homeostasis, studies in spinach, rocket salad [18], rice [19] and Cassava [20] have shown significant correlations between leaf minerals and diversity indices. Still, none have demonstrated a causative effect of leaf nutritional status on bacterial community distribution. These morphological and physiological differences can lead to uneven bacterial distribution across different plant types, underscoring the need for more integrative studies to understand the interplay between plant genetics, morphology, leaf nutrient content and microbial community structure.

While lettuce has not traditionally been used as a model organism, recent advances have considerably expanded our understanding of its evolutionary history and the molecular mechanisms underlying key plant traits. Notably, Wei and collaborators (2021) fully sequenced 445 *Lactuca* genotypes from accessions preserved by the Centre for Genetic Resources in the Netherlands (CGN). This extensive sequencing effort generated a detailed variation map, combined with available phenotypic traits, providing a valuable resource for exploring phylogenetic relationships and advancing breeding programs.

In this work, we took advantage of this comprehensive collection to conduct a large-scale field experiment using 131 *Lactuca sativa* genotypes. We hypothesized that key and manipulable plant determinants, such as genetic distance, leaf micro- and macronutrient content, and leaf morphology, may influence the leaf-associated bacterial community establishment and differentiation. Taking into account the complex interdependencies existing between plant genotype, phenotype and physiology [10, 21, 22], we uncovered the importance of considering phenotype-microbiome association to better understand, and possibly predict, the phyllosphere microbiota.

## Materials and methods

### Experimental design

 In this study, 131 fully sequenced genotypes of *Lactuca sativa* coming from 26 different countries were selected from the Centre for Genetic Resources (CGN, Wageningen, Netherlands). Non-sterilized seeds were sown and germinated in a growth chamber for 24 h at 21 °C with maximum humidity and then grown under greenhouse conditions for nearly one month at Bronte Garden (Mira, Italy). On April 22nd, 2022 plants were transplanted in the soil of “Azienda Agricola Gambaro” greenhouse (Noale, Italy), where they grew for another month. For the experiment, nine replicates per genotype were transferred into three blocks labeled as “A,” “B,” and “C” in the soil, following a randomized design outlined in Table S1. Each block consisted of 20 lines (numbered 1 to 20) and 20 columns (named a to t), maintaining a plant distance of 20 × 20 cm. Three replicates per genotype were cultivated inside each block. Throughout the experiment’s duration, plants were irrigated using drip irrigation. The entire experimental procedure is reported in Fig. S1.

### Sample collection

 Leaf sample collection took place on May 20th–21st, 2022. During this process, a leaf from the mid-upper part of each plant was selected. Prior to sampling, leaves underwent surface washing with sterile water. The sample collection was done in racks of 96 collection microtubes (QIAGEN Hilden, Germany), following the sequential order outlined in Table S2. Control soil samples (germination substrate, growth soil stage 0, growth soil stage 1 and endophytes) were collected at three different time points as detailed in supplementary materials and methods. Other control samples used in this work included seeds and the leaf endophytes (Table S3, supplementary material and methods). Immediately after collection, all samples were transported to the laboratory on ice and stored at −20 °C. Leaf disks were lyophilized for long-term storage.

### Leaf mineral content quantification

 Leaf disks were used to quantify leaf micro- and macro-nutrient content using the Inductively Coupled Plasma-Optical Emission Spectroscopy (ICP-OES) technology at SESA (Este, Italy). Briefly, samples were weighted with an ultra-analytical balance (sensitivity = 0.01 mg) and then digested at 95 °C for 2 h in nitric acid (HNO3 67–69%) using the DigiPREP (SCP Sciences). Samples were then diluted in 20 ml of ultrapure water and filtered through 25 mm diameter, 0.45-micron pore-size, removable syringe-tip filter membranes (cellulose acetate). 50 µl of yttrium was added as an internal standard to all samples before initiating the ICP-OES detection. Minerals that were poorly detected were excluded from the analysis. For the predominantly detected ones, samples falling below the limit of quantification (< LOQ) were fit in the model, following the procedure reported in a published method [23]. The detailed above-mentioned data and the processing steps are reported in Table S4.

### DNA extraction and sequencing

The leaf disk DNA isolation was performed on a large scale using BioSprint® technology (QIAGEN, Hilden, Germany) in 96-well collection microtubes. Seven empty wells were included as controls to identify potential contaminants from the extraction process. Conversely, DNA extraction from soil and seed samples was carried out using the DNeasy® PowerSoil® kit (QIAGEN Hilden, Germany) following the manufacturer’s instructions.

 To isolate endophytes, a pool of 131 leaves underwent surface sterilization following the procedure outlined in a previous study [24] with some modifications specified in supplementary material and methods and Fig. S1B. DNA isolation was then carried out using the CTAB protocol. DNA from all samples was quantified using Qubit™ dsDNA Assay Kits from Thermo Fisher Scientific, and their concentrations are detailed in Table S5. Subsequently, the DNA samples underwent MiSeq Illumina 16S amplicon sequencing at IGA Technology Services (IGATech, Udine, Italy) following the procedure reported in supplementary material and methods.

### Raw reads processing

 Raw sequencing reads were subjected to processing using Dada2 (v.1.10) in QIIME2 (QIIME2-2023.2). The resulting dataset underwent additional refinement in R (version 4.3.2) to eliminate chloroplasts, eukarya, archaea, mitochondria (Table S6) and possible contaminations that occurred during extraction using the Decontam package (version 1.24.0). The final dataset consists of 12,173 ASVs and 417 samples. R’s Vegan package (version 2.6–4.6) was employed to rarefy the dataset in a size-dependent manner. Given the disparate sampling depths across compartments, each was rarefied to a distinct sampling depth (leaf: sample size = 1,000 reads, soil: sample size = 14,000 reads, seeds: sample size = 200 reads, endophytes: sample size = 4,500 reads). Using the Phyloseq package (version 1.44.0), the rarefied datasets were merged into a Phyloseq object, comprising 9,896 ASVs and 256 samples (9,125 leaf ASVs and 230 leaf samples).

### Identification of groups of closely related genotypes and varieties

 The lettuce phylogenetic tree, as delineated by [25], served as the basis for extracting the coordinates of the *Lactuca sativa* accessions tree. These were subsequently utilized to construct a novel phylogenetic tree in ITOL. A distant member belonging to the *Lactuca saligna* species (TKI-349) was selected as the outgroup to root the tree. Six distinct groups of closely related genotypes were identified based on branch ages. Seven breeder-defined varieties were used as proxies for plant phenotypes. Two genotypes, TKI-071 and TKI-119, did not clearly belong to any genetic distance group and were thus omitted from the analysis. Additionally, TKI-11 was excluded due to a lack of tree-coordinate information, and TKI-92 was removed because there were insufficient phenotypic parameters to impute the missing values. This process resulted in the exclusion of the oilseed variety group. The remaining samples were distributed as described in Table **S7**.

### Community composition and contributions of plant determinants to β diversity

To investigate statistically significant differences at various taxonomic ranks (up to the genus level) between groups of closely related genotypes and varieties, the Kruskal-Wallis test with Dunn’s *post hoc* test (*p-adjusted* method = “fdr”) was applied. Adjusted p-values less than 0.05 were considered significant. The hierarchical tree of the community at each taxonomic level was constructed using GraPhlAn (Graphical Phylogenetic Analysis), version 1.1.4 [26],, in Python3.

 β-diversity across leaf samples was assessed by the Bray-Curtis dissimilarity distance matrix in R using the vegan package. The influence of each plant factor in determining this diversity was tested with the Adonis2 implementation of Permutational Multivariate Analysis of Variance (PERMANOVA), taking into account the potential dependencies existing among variables. To disentangle the independent and combined proportions of variance explained by these significant determinants, we employed the varpart function from the vegan package. To ensure the effectiveness of varpart, missing values in the dataset’s phenotypic traits were imputed using the mice package (version 3.18.0) in R. Table **S8** represents the dataset before and after imputation. Other details are provided in supplementary material and methods.

### Integrative analysis of leaf bacterial communities: functional predictions, diversity, networks, and origins

Statistical differences in bacterial types between leaf morphological traits were assessed using the Kruskal-Wallis test with Dunn’s *post hoc* test.

Phenotypic traits exhibiting differences in α diversity were analyzed in the form of binary input values using the log2foldchange function from the DESeq2 package in R (version 1.40.2), applying the Benjamini-Hochberg multiple-testing correction to control the false discovery rate (fdr). The igraph package (version 2.0.3) was used to visualize the bacterial co-occurrence network for phenotypic groups showing differences in α diversity. The fast expectation–maximization microbial source tracking algorithm (FEAST) package (version 0.1.0 [27]), was used to calculate the relative share of ASVs between the leaf (considered the sink) and four different sources: seeds, germination soil, growth soil before (April 20, 2021) and after (May 21, 2021) plant transplantation, and the endophytic bacterial community.

A more detailed description of the methods and the script used for all the analyses are provided as supplementary material and methods and supplementary script, respectively.

## Results

### Genetic distance and variety influence leaf bacterial community composition and diversity

**Fig. 1:**
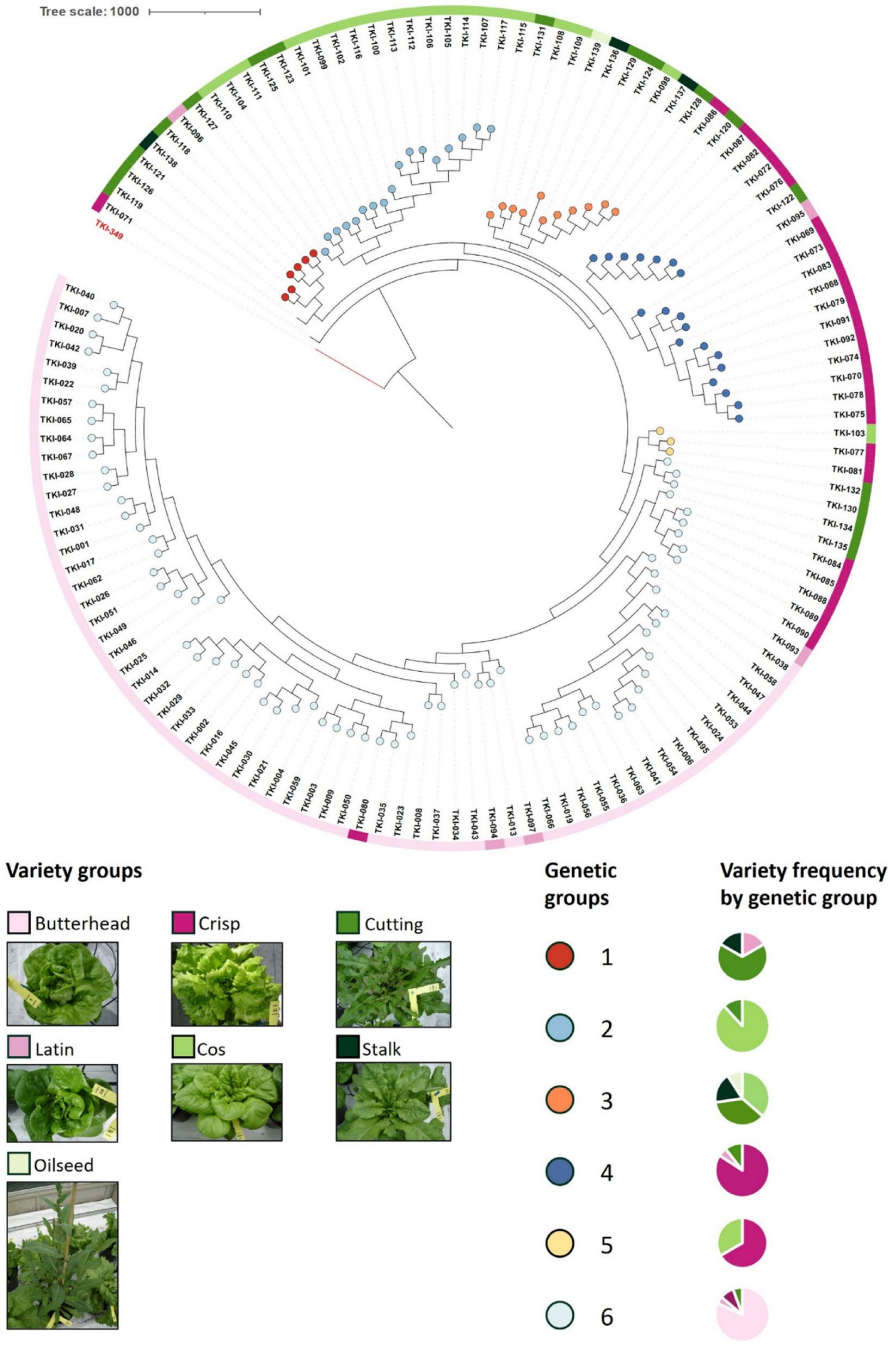
Neighbor-joining tree of 131 *Lactuca sativa* accessions. Tip colors indicate six groups of closely related genotypes, while colored squares represent different varieties. The phenotypic appearance of each variety group is depicted alongside. Bar charts show the proportion of each variety within each genetic group

To assess the impact of genetic distance and variety on the composition and shifts of leaf-associated bacteria, we first extracted and used the phylogenetic tree coordinates for 131 *Lactuca sativa* genotypes [25] to reconstruct the tree represented in Fig. 1. A partial overlap between variety groups and genetic distance groups was observed, as reported in Fig. 1 and Table 1. For instance, 81.94% of Butterhead plants overlap with genetic group 6, while Cos plants correspond mainly with group 2 (88.24%) and Crisp plants with group 4 (84.21%). However, other genetic groups (1, 3, and 5) are composed of several different varieties. The discrepancy between genetic groups and varieties underlies the importance of uncoupling phenotypic and genetic variation when studying the phyllosphere microbiota assembly.

**Table 1:** Percentage of each variety type within groups of closely related genotypes

	Butterhead	Cos	Crisp	Cutting	Latin	Stalk	Oilseed
1				66.67%	16.67%	16.67%	
2		88.24%		11.76%			
3		36.36%		36.36%		18.18%	9.09%
4			84.21%	10.53%	5.26%		
5		33.33%	66.67%				
6	81.94%		8.33%	5.56%	4.17%		

**Fig. 2:**
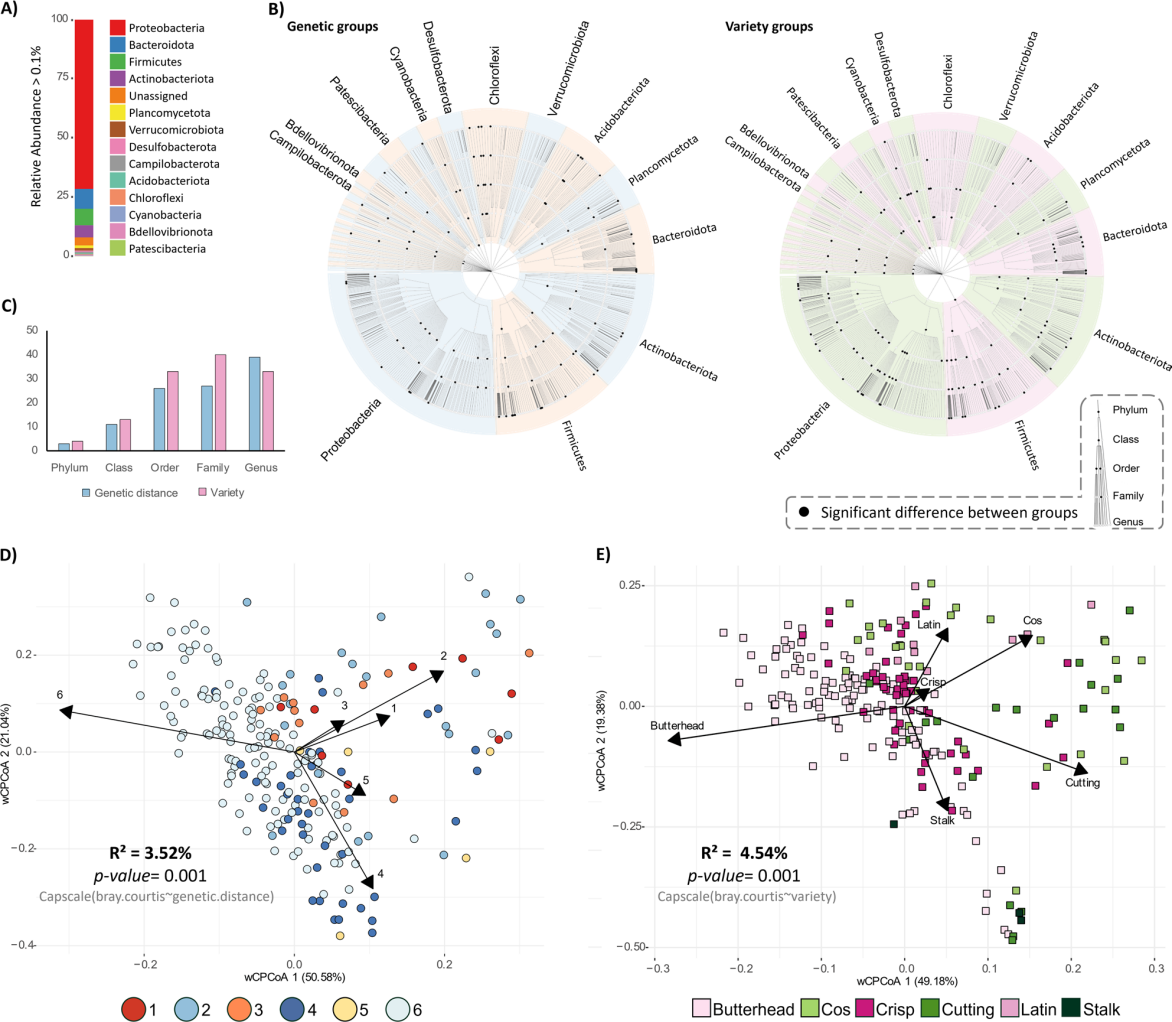
Leaf bacterial community composition and diversity as influenced by genetic distance and variety. **A** Stacked bar plot showing the most abundant community members (relative abundance > 0.1%), from top to bottom. **B** Significant differences in taxa between groups of closely related genotypes and varieties. Most abundant members of the leaf-associated bacterial community (relative abundance > 0.1%) are shown in the text. Nodes represent taxonomic ranks from Phylum (inner ring) to Genus (outer ring). Black dots indicate statistically significant differences of a node between groups of closely related genotypes (left side) and varieties (right side), assessed using Kruskal-Wallis and Dunn *post hoc* tests. Colors distinguish taxonomic groups between genetic groups (blue-to-red scale, left side), and varieties (pink to green scale, right side). **C** Disparities in significant nodes between varieties and groups of closely related genotypes across all taxonomic levels. **D-E** Constrained Analysis of Principal Coordinates (CAP) performed on the Bray-Curtis distance matrix showing the contribution to leaf-associated bacterial community β-diversity of groups of closely related genotypes, and varieties, respectively. Each circle/square represents a single sample. Arrows represent groups of closely related genotypes (left) and variety groups (right). Their length reflects the contribution of each variable to the underlying sample distribution, while their direction shows the orientation of the gradient, with samples positioned in the same direction having higher values for that variable

 Overall, the leaf bacterial communities across all *Lactuca sativa* genotypes (Fig. 2A) were dominated by Proteobacteria (71.3%), followed by Bacteroidota (8.4%), Firmicutes (7%), Actinobacteriota (5.1%), Planctomycetota (1.1%) and Verrucomicrobiota (1.1%). Bacterial composition varied between leaf and control samples, with a higher similarity observed among leaf, control seed, endophyte, and germination soil samples. In contrast, growth soils at stages 0 and 1 were predominantly dominated by Planctomycetota and Actinobacteriota, and they clustered distinctly away from the other samples in the Bray-Curtis distance matrix (Fig. S2, Table S9). In line with these results, the FEAST algorithm identified the seed and endophyte microbiomes as having the largest share of ASVs in common with the leaf microbiome (average contributions of 15.36% and 5.1%, respectively). This was followed by soil at the harvesting stage, soil at the transplanting stage, and finally, the germination substrate (Fig. S3A, C Table S10).

The effects of genetic distance and variety on bacterial community composition (Fig. 2B, C) involve variation in different community members (Table S11). At higher taxonomic ranks -from Phylum to Family- variety determines more bacterial shifts. However, at the genus level, genetic distance has a greater impact, affecting more genera (39) than those influenced by variety (33) (Fig. 2C, Table S11). The influence of plant variety on bacterial community composition is further evidenced by its impact on bacterial β-diversity, which slightly surpasses the contribution of genetic distance. Specifically, plant genetic distance accounted for 3.52% (PERMANOVA, p-value = 0.001) of the total variation in leaf-associated bacterial communities, while variety accounted for 4.54% (PERMANOVA, p-value = 0.001, Fig. 2D, E). Moreover, across varieties butterhead plants show significantly higher Shannon and observed index compared to Cos and Cutting plants (p-value < 0.05), while no difference in α-diversity is observed between genetic groups (Fig. S4). Altogether, these observations suggest that specific plant traits related to plant genotype and/or variety may differentially regulate leaf microbiota.

### Micro- (Fe, Zn, Mn, Na) and macronutrient (Ca, K, Mg, P) concentrations partially affect leaf-associated bacterial β-diversity

**Fig. 3:**
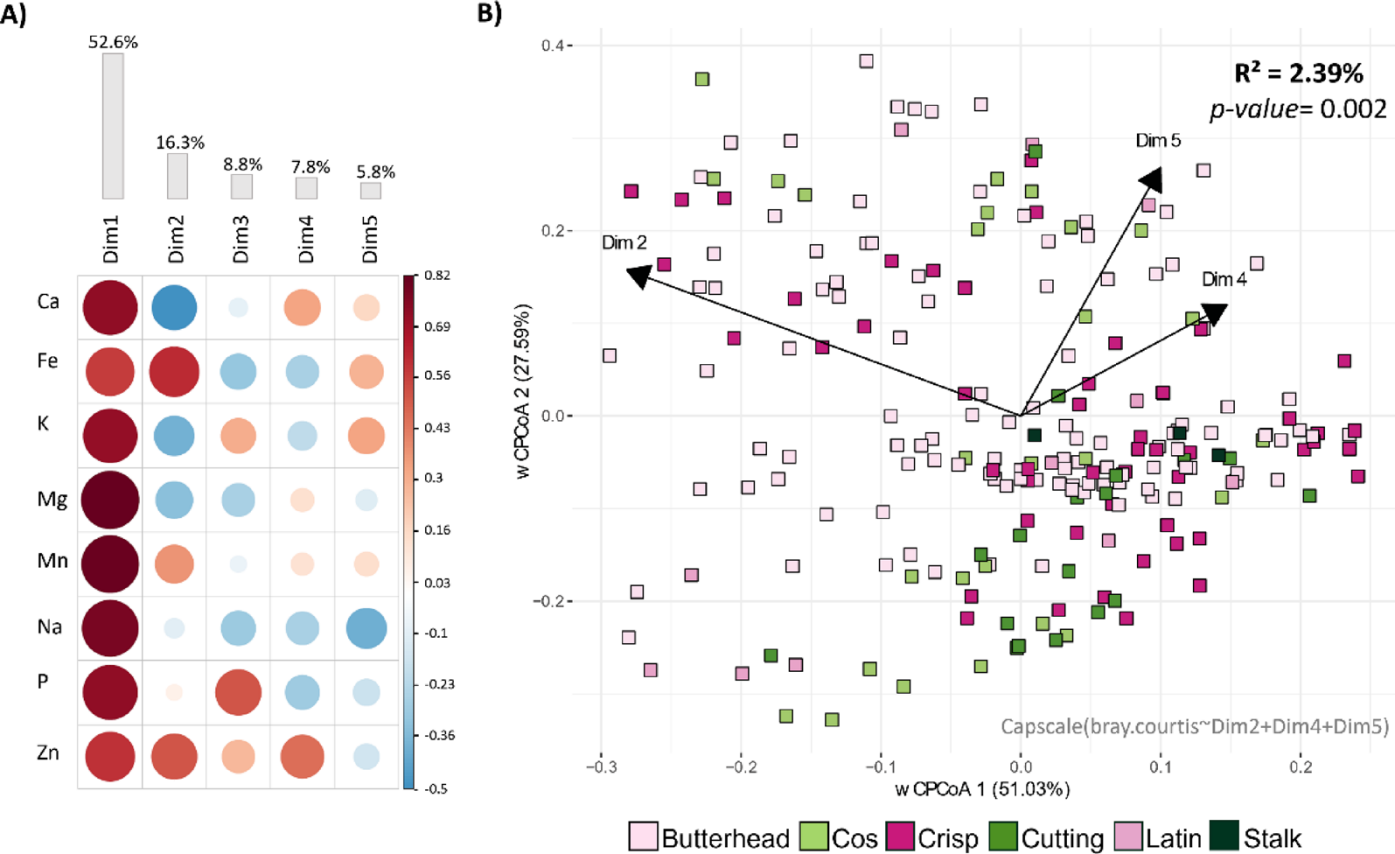
Correlogram and multivariate analysis linking mineral elements density with bacterial community distances. **A** Correlation matrix depicting the relationships between micro- and macronutrients and their PCs. Red indicates positive correlations, while blue indicates negative correlations. Larger dots indicate stronger correlations. **B** Constrained Analysis of Principal Coordinates (CAP) performed on the Bray-Curtis distance matrix illustrating the influence of significant mineral PCs (arrows) on bacterial diversity. Each square represents a single sample, color-coded according to the variety group. Arrows represent the three significant PCs, with their length reflecting the contribution of each variable to the underlying sample distribution, and their orientation indicating the direction in which the variable increases

 To further investigate the plant leaf traits that may explain the differences in bacterial β-diversity observed across genetic and variety groups, we focused on a subset of leaf micro- (Fe, Zn, Mn, Na) and macro- (Ca, K, Mg, P) elements. The strong correlations among most leaf elements (Fig. S5A) led us to perform a PCA ordination. Through this approach, we identified five PCs that collectively capture the combined contributions of these nutrient variables (Fig. 3A). Only PCs 2, 4, and 5, which capture opposing fluctuations between micro- and macronutrient concentration (Fig. S5B), had statistically significant small effects (PERMANOVA, p-value < 0.05) on shaping bacterial β-diversity (Table S12).

We constrained the bacterial Bray-Curtis dissimilarity distance matrix to reflect only the total variation explained by the significant dimensions 2, 4, and 5. The PERMANOVA statistical analysis revealed that leaf micro- and macro-nutrient content accounted for 2.39% (PERMANOVA, p-value = 0.002) of the bacterial β-diversity (Fig. 3B). In addition, they also demonstrated a minor impact on bacterial α-diversity with Dimension 2 being slightly significantly positively correlated with bacterial Shannon and observed indices (Fig. S6A, B).

Overall, these observations suggest that leaf nutritional status exerts a limited influence on the plant leaf-associated microbiota. Moreover, the results underscore the importance of considering the combined effects of micro- and macro-nutrients rather than their individual contributions (Fig. S5B-E).

### Leaf morphological traits disentangle the variety contribution to β-diversity

Given the significant impact of variety on bacterial variation among the three factors investigated, we hypothesized that increasing the resolution of this trait, particularly by including plant phenotypic parameters, could provide a more detailed understanding of factors shaping the leaf-associated bacterial community. We thus included 17 phenotypic parameters in the analysis (Table S8). These parameters, already characterized for each genotype by the collection curators and available on the CNG lettuce collection database, encompass a range of traits: leaf characteristics (e.g., shape, venation), pigmentation-related attributes (e.g., leaf color, anthocyanin content), as well as traits related to juvenile stages (e.g., seedling cotyledon shape) and adult plants (e.g., heart formation, head height).

 The PERMANOVA statistical analysis revealed that out of the 17 phenotypic parameters investigated, 9 were significantly (PERMANOVA, p-value < 0.05) associated with leaf bacterial β-diversity (Table S13). Among these, the most impactful parameter was head shape (R^2^ = 0.012, p-value = 0.001), followed by heart formation (R^2^ = 0.012, p-value = 0.001), head height (R^2^ = 0.01, p-value = 0.001) and leaf venation (R^2^ = 0.018, p-value = 0.001).

**Fig. 4:**
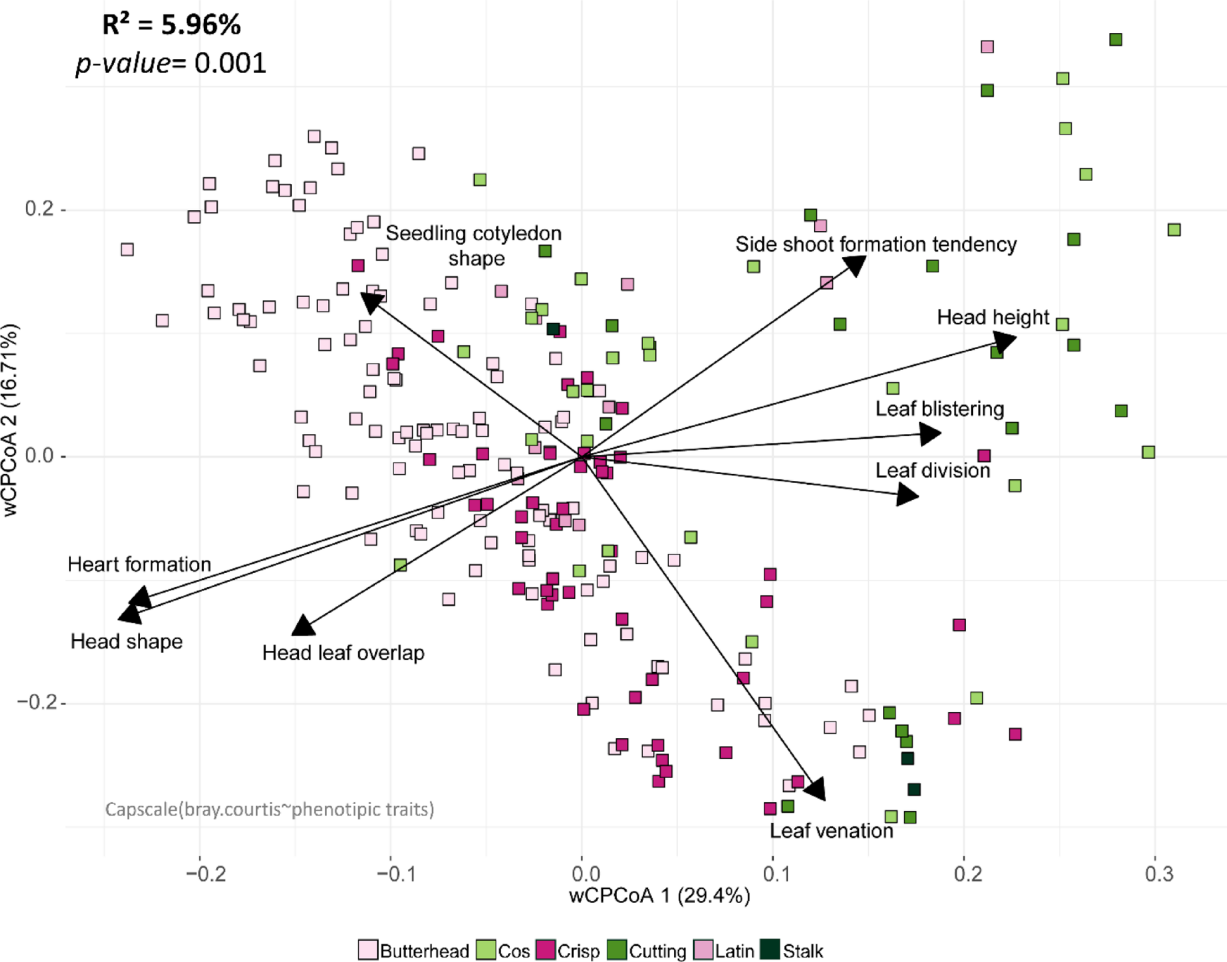
Multivariate dissection of the variety contribution to bacterial β-diversity. Constrained Analysis of Principal Coordinates (CAP) performed on the Bray-Curtis distance matrix illustrating how leaf morphological traits (arrows) contribute to leaf-associated bacterial β-diversity. Samples are color-coded according to the variety groups to emphasize their association with plant phenotypes. Each square represents a single sample. Arrows represent the plant morphological traits studied, with their length reflecting the contribution of each variable to the underlying sample distribution, and their orientation indicating the direction in which the variable increases

We found that the above-mentioned leaf morphological traits explained 5.96% of the variation in bacterial community composition (PERMANOVA, p-value = 0.001, Fig. 4) and also allowed a clear clustering among different lettuce varieties. For example, Butterhead lettuce, known for its tight heart formation capacity, is strongly associated with high values in traits such as head shape, heart formation, and head leaf overlap.

**Fig. 5:**
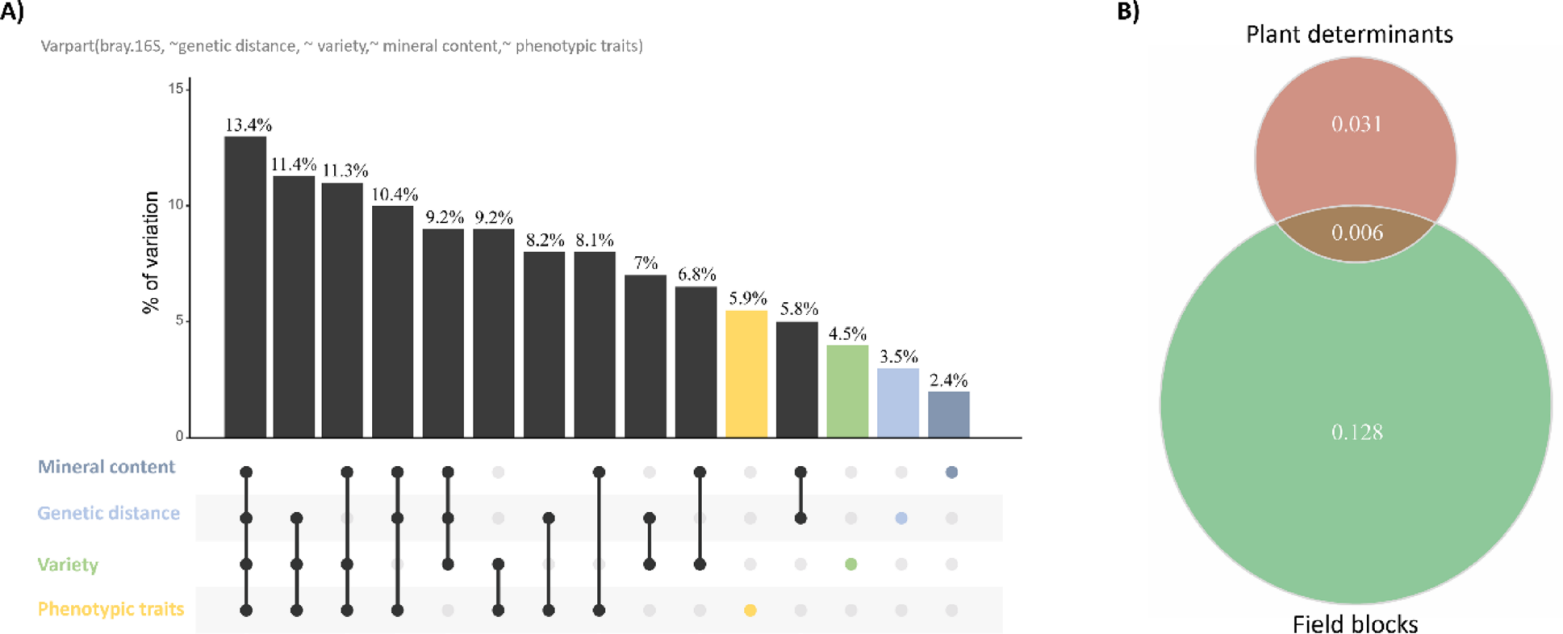
Proportion of leaf-associated bacterial 16 S diversity explained by plant determinants. **A** UpSet plot representing the proportion of bacterial β-diversity (R²) explained by each individual plant determinant and their combinations. Colors distinguish the contributions of individual variables from those of combined variables (in black). **B** Venn diagram showing the individual contributions and the overlap of known plant and environmental factors to leaf-associated bacterial β-diversity

 To dissect the individual and combined contributions of genetic distance, variety, leaf micro- and macronutrient content, and plant morphological parameters, we conducted a Variation Partitioning analysis. The results align with those from the Constrained Analysis of Principal Coordinates, especially in terms of the variance (R^2^) explained by each individual trait (Figs. 2D and E, 3, 4 and 5). Collectively, these factors accounted for 13.4% of the total β-diversity in the leaf-associated bacterial community. Figure 5A shows some overlap in the R^2^ explained by these factors, but their combined effect never exceeds the sum of each individual factor (Table S14-S15).

In summary, these results suggest that despite the substantial environmental exposure of leaves, a significant portion of bacterial diversity is still linked to intrinsic plant characteristics. These factors influence diversity at multiple levels, including bacterial composition, α-diversity, β-diversity, with leaf morphological traits contributing with the largest share.

### Non-hub community members modulate leaf bacterial composition and richness in relation to shoot phenotypic traits

**Fig. 6:**
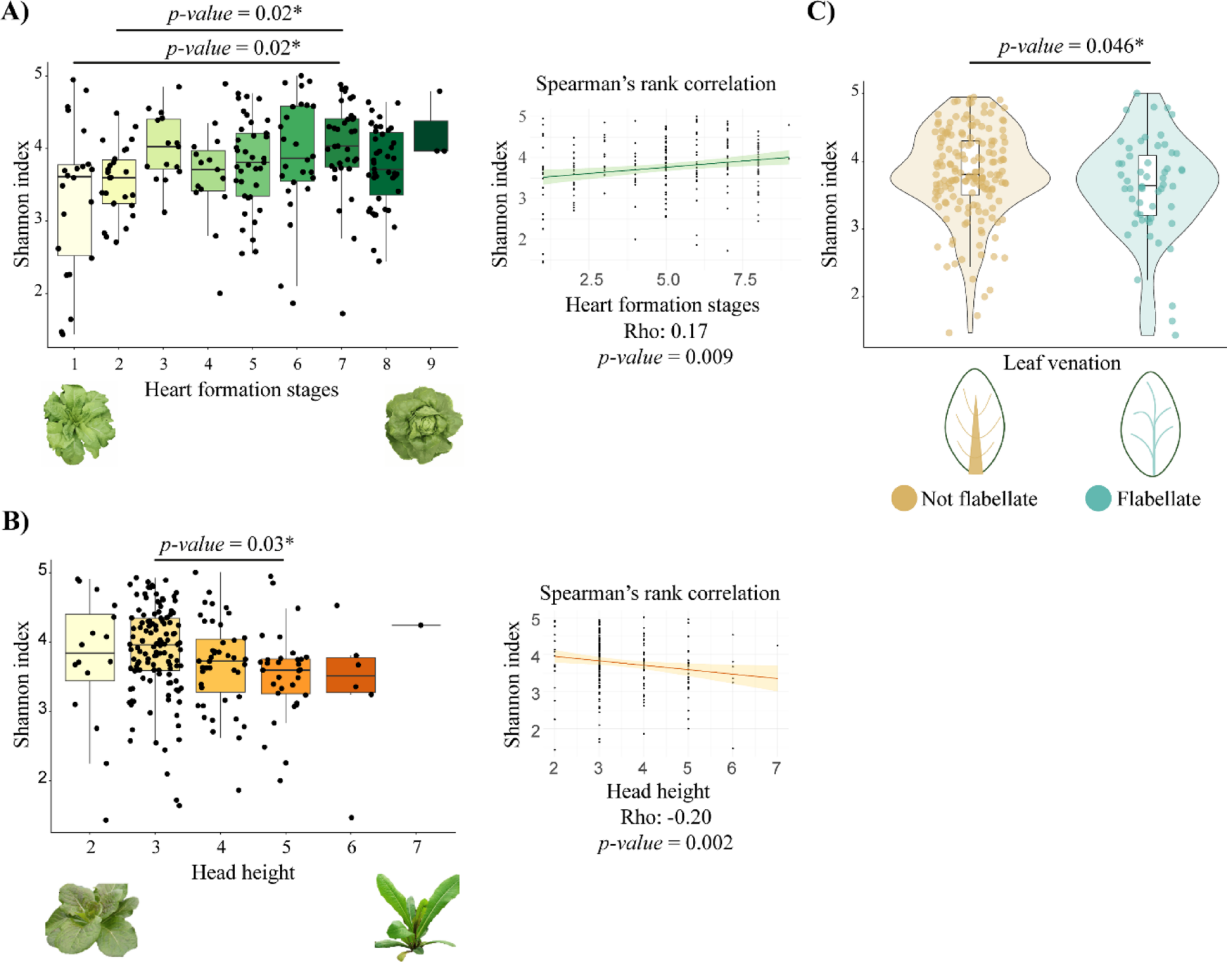
Effect of phenotypic traits on bacterial α-diversity. **A-C** Differences in Shannon diversity index across **A** Heart formation stages, **B** Head heights, and **C** Leaf venation types. Statistical differences between groups were assessed using Dunn *post hoc* and Wilcoxon–Mann–Whitney tests. Spearman’s rank correlation coefficient was used to evaluate the relationship between the tested variables and the α-diversity Shannon index in panels **A** and **B**. Credit: created in BioRender. Capparotto, A. (2024) BioRender.com/f29j677

 After assessing the homogeneous distribution of phenotypic trait groups across field blocks (Fig. S7), we found that among the significant phenotypic traits, heart formation and leaf venation, which explained most of the microbiota variation and drive distinct microbial communities, strongly influenced α diversity (Fig. 6, Fig.S8). This observation aligned with results obtained from bacterial metagenome-assembled genomes (MAGs) within the lettuce genome project (Fig. S9, Table S16). The grouping values for these parameters were previously characterized by breeders. Specifically, significant differences in Shannon and Observed α-diversity (Dunn *post-hoc*, p-value < 0.05) were observed between different heart formation stages and plant heights, with a positive correlation between the Shannon index and heart formation stages (Spearman Rho = 0.17, p-value = 0.009) and a negative correlation between the Shannon index and plant height (Spearman Rho = −0.20, p-value = 0.002, Fig. 6A, B, Fig. S8 and and Fig. S9).

**Fig. 7:**
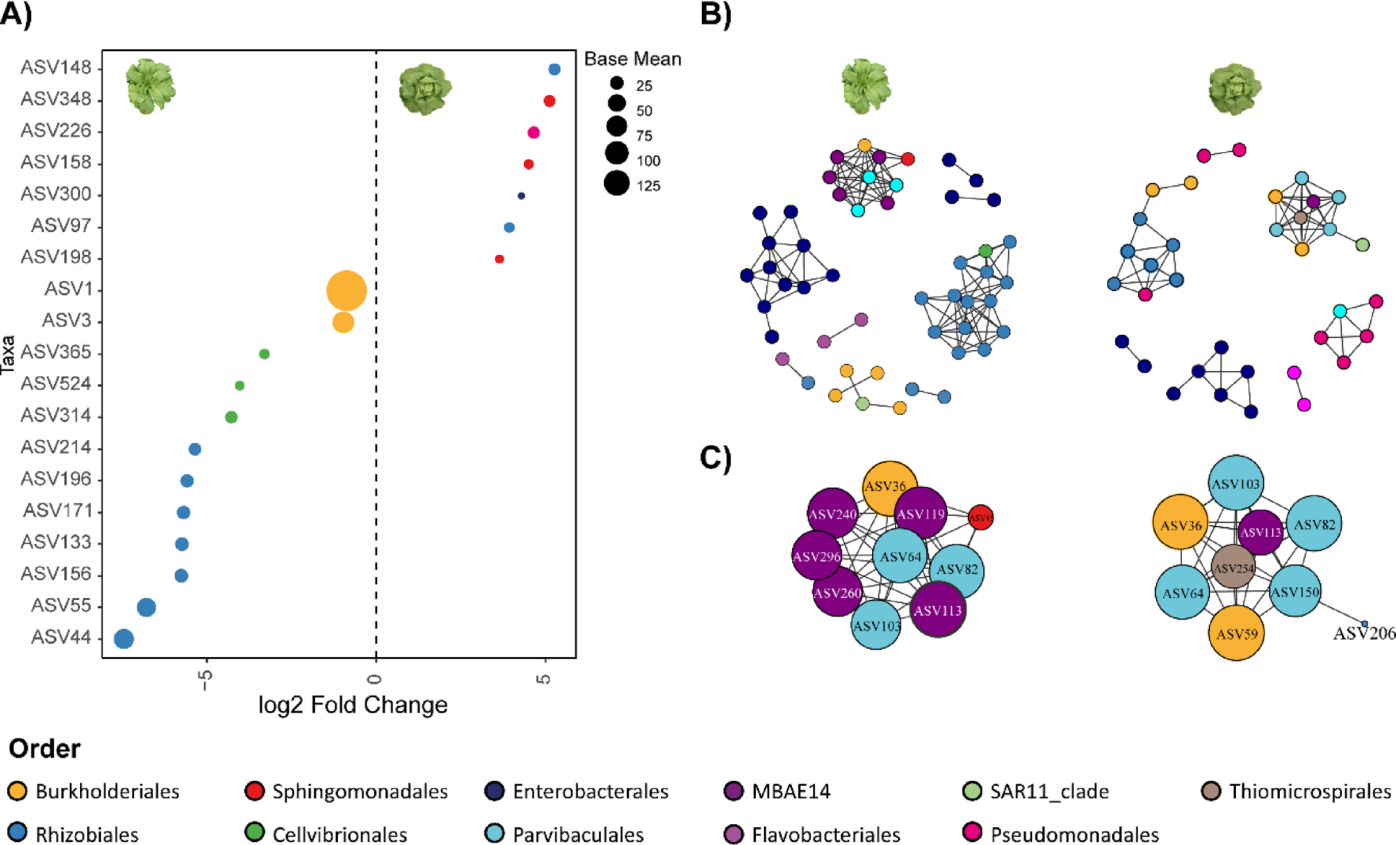
Identification of differentially abundant ASVs between heart formation capacity groups. **A** ASVs with significant changes in relative abundance (p-value < 0.05) between groups 1 and 2 (open-hearted plants, left plot) and group 7 (tight-hearted plants, right plot). Colors correspond to the ASVs classification at the order level, as indicated in the legend. The size of the ASVs represents the base mean effect size, indicating the magnitude of change relative to the reference. **B** Leaf bacterial community network in open-hearted plants (left) and tight-hearted plants (right). Dots represent ASVs, colored according to their order. Positive correlations are shown in black and only significant ones (Spearman correlation > 0.65) are depicted. **C** Hub ASVs of the community. Colors indicate the order, while size corresponds to the hub score

 Additionally, leaf venation was found to impact bacterial α-diversity (Fig. 6C). Indeed, not-flabellate venation types host a bacterial community with a significantly higher Shannon index compared to flabellate types (Wilcoxon–Mann–Whitney test, p-value = 0.046). To investigate which ASVs are differentially enriched between these phenotypic groups and whether these changes are driven by hub taxa within the community, we conducted differential abundance analysis (Fig. 7A) and community co-occurrence network analysis (Fig. 7B, C). Notably, none of the ASVs that differ in abundance between plant types are hub taxa (Fig. 7A), except for ASVs 36 and 59 from the Burkholderiales order and ASVs 103 and 82 from Parvibaculales, identified as a hub taxon in flabellate venation type (Fig. S10A, C). Across all traits analyzed, hub members mostly belong to the Parvibaculales, MBAE14, Burkholderiales, and Thiomicrospirales orders, remaining stable across phenotypic traits (Fig. 7C, S10A, B). The only exception is leaf venation type (Fig. S10A, C), where hub members differ between non-flabellate and flabellate groups. The non-flabellate group is almost entirely composed of Rhizobiales, while the flabellate group displays a pattern similar to that observed in heart formation and head height. Fig. S11 also highlights that the hierarchical cluster, according to bacterial relative abundance across heart formation stages, clearly isolates hub taxa from the remaining bacterial community.

Overall, these findings suggest that changes in ASV relative abundance do not involve hub taxa, which remain stable and show only minor variations between shoot morphologies. This implies that the observed diversity between groups with contrasting phenotypic traits is driven by shifts in the more abundant ASVs that are peripheral within the community network.

In summary, our findings indicate that despite the considerable environmental exposure of leaves, a significant portion of bacterial diversity is strongly associated with intrinsic plant characteristics, with leaf morphological traits playing a pivotal role. These traits influence diversity on multiple fronts, including α-diversity, β-diversity, and the enrichment of specific ASVs, without affecting hub species.

## Discussion

Building on the concept that plant phenotypic traits and leaf nutrient content are direct consequences of the plant’s genetic makeup, which in turn shapes the structure of the phyllosphere bacterial community [10], we investigated the complex interplay among these factors. Specifically, we examined how different leaf physicochemical features, partially driven by genotype, influence the structure and diversity of leaf-associated bacterial communities.

Although the individual contributions of genotype, micro- and macronutrient content, and phenotypic traits may be modest, together they account for up to 13.4% of the total diversity of leaf-associated bacterial communities. This suggests that, despite the substantial influence of environmental variation on bacterial communities, plant-specific determinants still allow for the prediction of a considerable piece of diversity observed in leaves [28, 29]. This is in line with other works on lettuce [13, 14, 17, 30] and Arabidopsis, where the genotype has been reported to explain nearly 10% of the variation in phyllosphere-associated bacterial communities across three studied genotypes [31]. Accordingly, GWAS on Arabidopsis reported that the host genome explained around 10% of the variance of phyllosphere bacterial and fungal communities [32, 33]. Interestingly, in our experimental setup, leaf morphological traits played a central role in setting the plant-bacterial interaction stage compared to genetic distance and leaf micro- and macronutrient content, in line with results on spinach, rocket salad and *Ipomoea hederacea* [18, 34]. This insight is crucial for breeders, as it highlights the potential to manipulate leaf microbiome through plant traits and to discriminate between leaf morphology’s direct and indirect effects, as shown by plant-derived metabolites from silver birch [35].

Among all analyzed traits, shoot morphological characteristics showed a great influence on causing shifts in both α- and β-diversity. These findings are consistent with those of Hunter et al., 2010, which highlighted the role of plant morphotype in differentiating bacterial profiles in lettuce. More specifically, our results indicate that increased heart formation capacity, decreased head heights and a not-flabellate leaf venation type are associated with greater species richness. Interestingly, the same correlations were evident when focusing on MAGs from the whole-genome *L. sativa* sequencing project [25], Fig. S9. We hypothesize that the development of a tightly formed heart may create a distinct ecological niche compared to plants with a more open structure, altering water retention and distribution, and favoring bacterial proliferation [36, 37]. In the case of low head height, the proximity to the soil can expose leaves to a greater reservoir of soil bacteria, increasing the potential for leaf bacterial contamination.

In addition to α-diversity, we reported how the different plant forms associate divergent bacterial members. For example, open-hearted plants were enriched with several ASVs from the Rhizobiales, Cellvibrionales, and Burkholderiales orders. Notably, members of the Rhizobiales and Burkholderiales orders are generally recognized as soil bacteria [38–40], suggesting that their presence in open-hearted plant types may be due to larger environmental exposure, similarly to plants grown close to the soil surface [14, 38]. Notably, two Enterobacteriaceae ASVs enriched in shorter plants belong to the genus *Pantoea*, whose members have been shown to inhibit the growth of certain human pathogens in both pre-and post-harvest conditions [41, 42]. For leaf venation the ASVs enrichment in the non-flabellate type, suggests that in this venation form, where a midrib gives rise to other veins, vein density may be higher, creating more favorable conditions for bacterial proliferation than in flabellate venation types, where veins grow radially from the base [43].

In accordance with the results of Damerum and colleagues [17], positive interactions between bacterial members dominated the community (Fig. 7C, S10). At most taxonomic levels, variety was the primary plant driver of community changes; however, at the genus level, genetic distance had a greater influence, suggesting a possible role of genotype fine-tuning in shaping the leaf bacterial community [44, 45].

Overall, our findings highlight the benefits of working with large and well-documented datasets and underscore the importance of enhancing phenotyping efforts to increase the resolution of analyses. In this study, we explored the complex interconnection of three plant factors: the morphological traits of the leaves, the genotype, and the micro and macronutrients, examining their individual and combined effects on the leaf-associated bacterial communities. Altogether, plant-related factors can explain about 13,4% of the leaf-associated bacteria, while 25% of the leaf microbiota could potentially originate from seeds [46] and soil (Fig. S2 and Fig. S3). These observations lay the groundwork for future studies aimed at developing plant varieties with an enhanced ability to support beneficial microbiota and for further investigation into the unknown factors that shape leaf bacterial communities.

## Limitations of the study

In the present work, some limitations should be considered. First, the sample sizes of the groups of closely related genotypes and varieties are uneven. However, we made every effort to (i) select all the *Lactuca sativa* genotypes available in the CNG collection and (ii) define genetic groups to have a comparable number of varieties, while also being as representative as possible of genetic distances. Despite these efforts, the contributions reported in this paper are relatively low and are primarily intended to guide the direction of future studies.

Additionally, the low bacterial counts on the leaf surface, combined with large chloroplast contamination, resulted in a substantial loss of reads compared to the initial input and a reduction in the number of samples. However, we prioritized read quality over quantity, choosing a threshold that allowed us to maintain a balance between sample loss and the number of ASVs, which aligns with values commonly reported in the literature for leaf microbiomes. Finally, we are aware that the large number of explanatory variables (9 leaf morphological traits) and their correlations, could have led to an overinflation of the model in Fig. 4. However, those traits are statistically significant when considered individually and their variation is also associated with varying α-diversity (Fig. 5).

## Supplementary Information

Below is the link to the electronic supplementary material.

Supplementary Material 1

Supplementary Material 2

Table S4

Table S4

## Data Availability

The authors confirm that the data supporting the findings of this study are available within the article and its supplementary materials. The leaf micro- and macro-nutrient content data are detailed in Table S4 of the supplementary information. DNA sequences generated by 16S amplicon sequencing have been deposited in the NCBI SRA repository and can be accessed via the following DOI: [https://identifiers.org/ncbi/bioproject: PRJNA1173114](https:/identifiers.org/ncbi/bioproject: PRJNA1173114). The dataset on statistically different taxa among varieties and genetic distance groups is provided in Table S11 of the supplementary information. The leaf-associated bacterial community composition data are available in Table S9 of the supplementary information.
