# Supplementary material for: Plant phenotypic differentiation outweighs genetic variation in shaping the lettuce leaf microbiota
Source: Environ Microbiome. 2026 Jan 30;21:35. doi: 10.1186/s40793-026-00850-6 (PMC12931058; doi:10.1186/s40793-026-00850-6)
Supplement: Supplementary file 1 — Supplementary Material 1 [file 40793_2026_850_MOESM1_ESM.pdf]

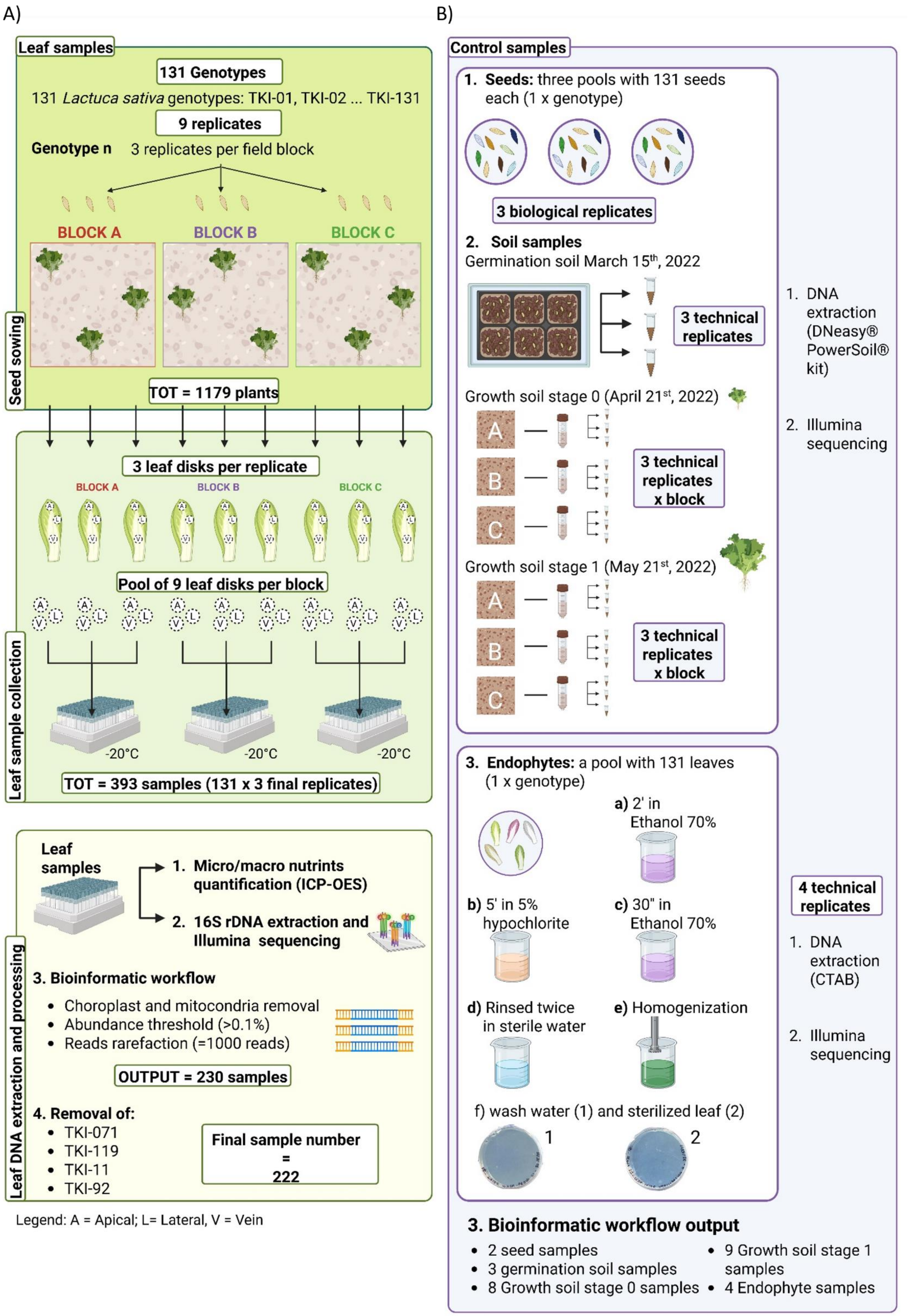


**Fig. S1. Workflow of sample collection and processing.**
**A)** Experimental procedure for sowing lettuce plants, leaf sampling, and bioinformatic analysis. **B)** Experimental workflow for control sampling, including seeds, germination soil, growth soil at stage 0, and growth soil at stage 1, including laboratory and bioinformatic processing. Credit: Created in BioRender. Capparotto, A. (2025) https://BioRender.com/p84g330 .

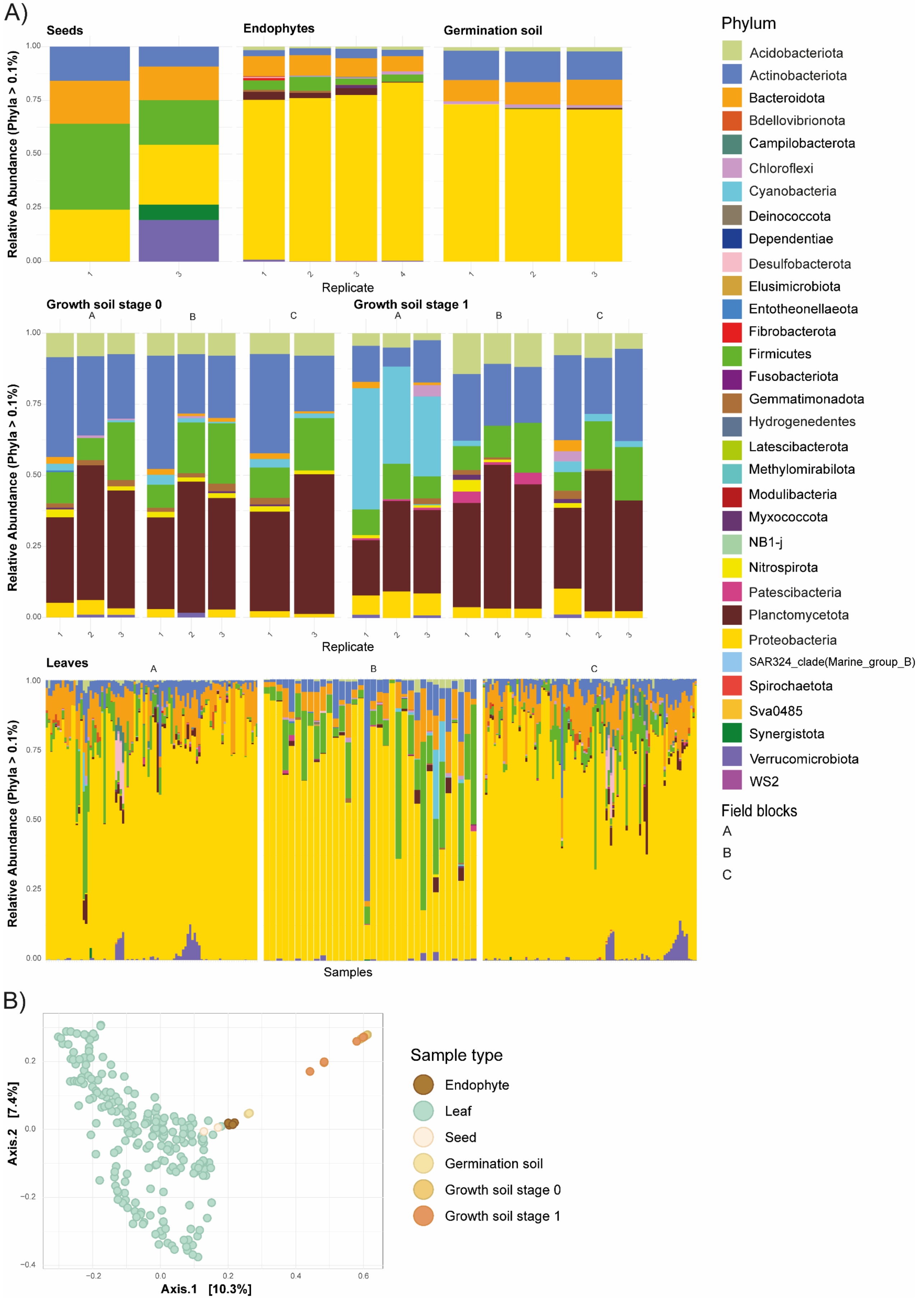


**Fig. S2. Bacterial phylum community composition (relative abundance >0.1%) across different samples.**

**A)** stacked bar plot showing the phylum composition in seed, endophyte, germination soil, growth soil at stage 0, growth soil at stage 1, and leaf samples. Each bar represents a sample replicate, while "A", "B", and "C" indicate the field block positions from which the samples were collected. **B)** Bray-Curtis dissimilarity distance matrix illustrating the beta diversity across the different sample types.

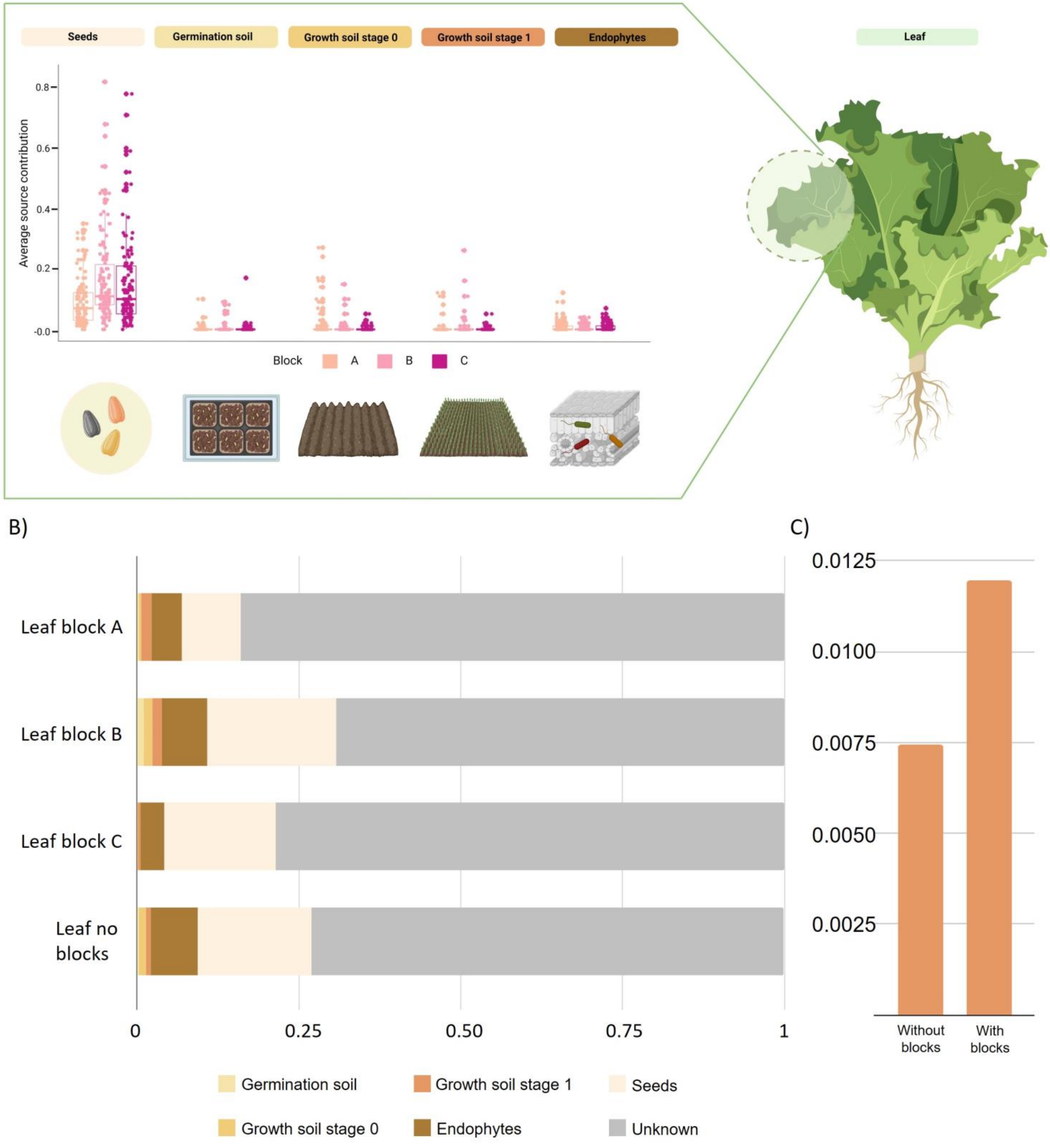


**Fig. S3. Estimated sources of the leaf-associated bacterial community.**
**A)** The share of each source (seeds, germination soil, growth soils, and endophytes) in contributing to the leaf bacterial community according to FEAST. Contributions were calculated separately for each sample within the three field blocks, which are represented by different colors in the boxplots. **B)** Proportions of known and unknown contributions based on analyses by blocks (Leaf Block A, B, and C) and a separate analysis conducted without considering field blocks (last bar). **C)** Variation in the average source contribution for growth stage 1, comparing results without considering blocks (first bar) and with blocks taken into account (second bar). Credit: created in BioRender. Capparotto, A. (2024) BioRender.com/a47q702.

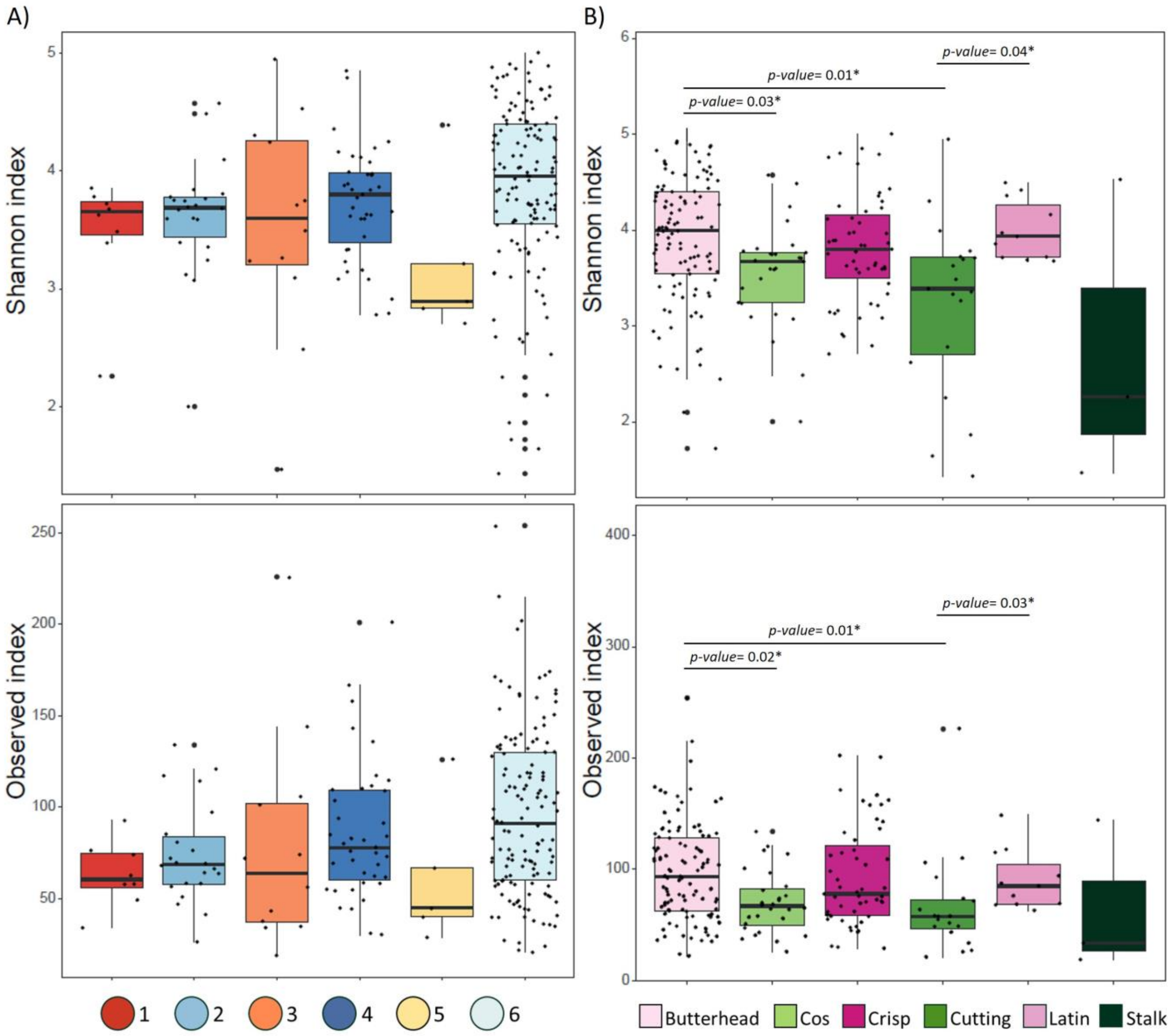


**Fig. S4. α-diversity variation across groups of closely related genotypes and varieties.**
Shannon (Above) and Observed (below) indexes were used to evaluate richness across different genetic groups **(A)** and varieties **(B)**. Statistical differences between groups were evaluated by Kruskal-Wallis followed by Dunn *post-hoc* tests.

A)

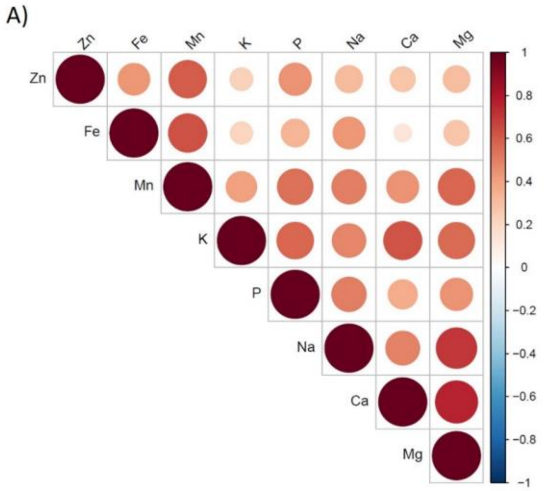


C)

Adonis2(bray.16S~element)

| Element | $R^2$ | *p-value* |
|---|---|---|
| K | ns | ns |
| Ca | ns | ns |
| Mg | ns | ns |
| Na | ns | ns |
| Fe | ns | ns |
| P | ns | ns |
| Mn | ns | ns |
| Zn | ns | ns |

B)

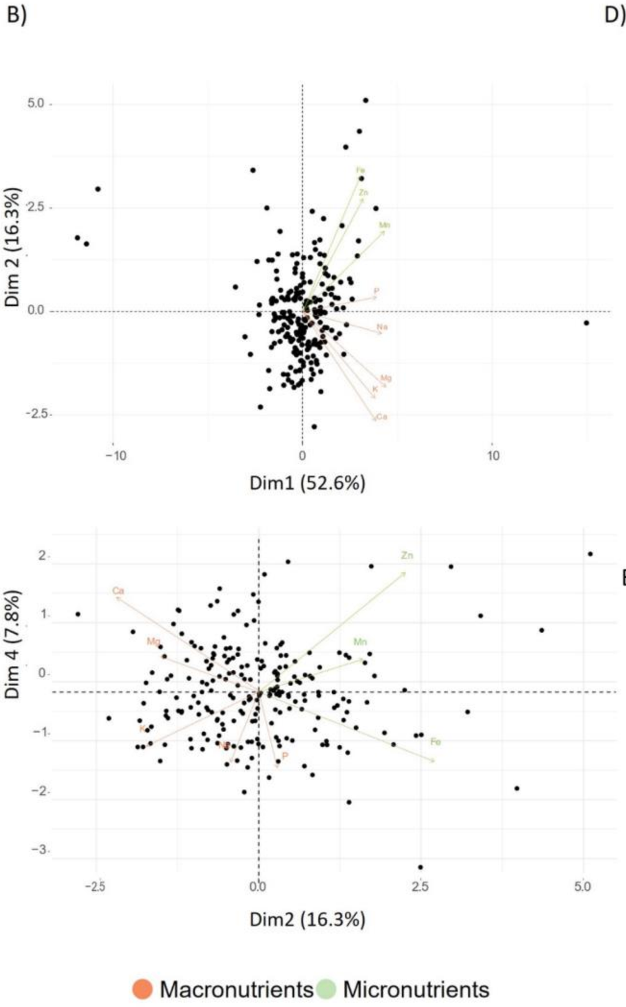


D)

Adonis2(bray.16S~variety(or genetic distance)/element)

| Element | Genetic distance | variety |
|---|---|---|
| K | ns | ns |
| Ca | ns | ns |
| Mg | ns | ns |
| Na | ns | ns |
| Fe | ns | ns |
| P | ns | $R^2$=0.034, *p-value*=0.002 |
| Mn | ns | ns |
| Zn | ns | $R^2$=0.031, *p-value*=0.014 |

E)

| Variable | Dim1 | Dim2 | Dim3 | Dim4 | Dim5 |
|---|---|---|---|---|---|
| **Ca** | 12.528 | 18.862 | 1.124 | 16.826 | 5.959 |
| **Fe** | 7.778 | 28.902 | 14.109 | 11.428 | 17.081 |
| **K** | 12.229 | 11.855 | 13.052 | 6.940 | 22.710 |
| **Mg** | 16.133 | 8.980 | 10.427 | 2.993 | 2.753 |
| **Mn** | 15.571 | 10.186 | 0.671 | 2.570 | 4.546 |
| **Na** | 14.488 | 0.752 | 12.572 | 11.674 | 34.595 |
| **P** | 12.723 | 0.327 | 37.778 | 13.649 | 6.781 |
| **Zn** | 8.551 | 20.137 | 10.267 | 33.920 | 5.575 |

**Fig. S5. Combined and independent mineral contributions to bacterial β-diversity.**
**A)** correlation matrix among micro- and macroelements. Colors differentiate positive correlations (in red) from negative correlations (in blue), with color intensity indicating the strength of the correlation (darker colors represent stronger correlations). **B)** Principal component analysis (PCA) of leaf micro- and macronutrient contents. Colors differentiate macronutrients (in pink) from micronutrients (in green). The upper panel shows the distribution along the first dimension (which does not significantly impact β-diversity) and the second dimension (which significantly influences β-diversity). The lower panel illustrates nutrient distribution according to dimensions 2 and 4, which account for significant variation in β-diversity. **C)** Independent mineral contributions to bacterial β-diversity. PERMANOVA analysis was performed to determine the effects of individual minerals on β-diversity. **D)** Nested mineral contributions to bacterial β-diversity by genetic distance and variety groups. PERMANOVA was applied to reveal effects. **E)** percentage contribution of each variable to every principal component (PC) obtained from the PCA.

A)

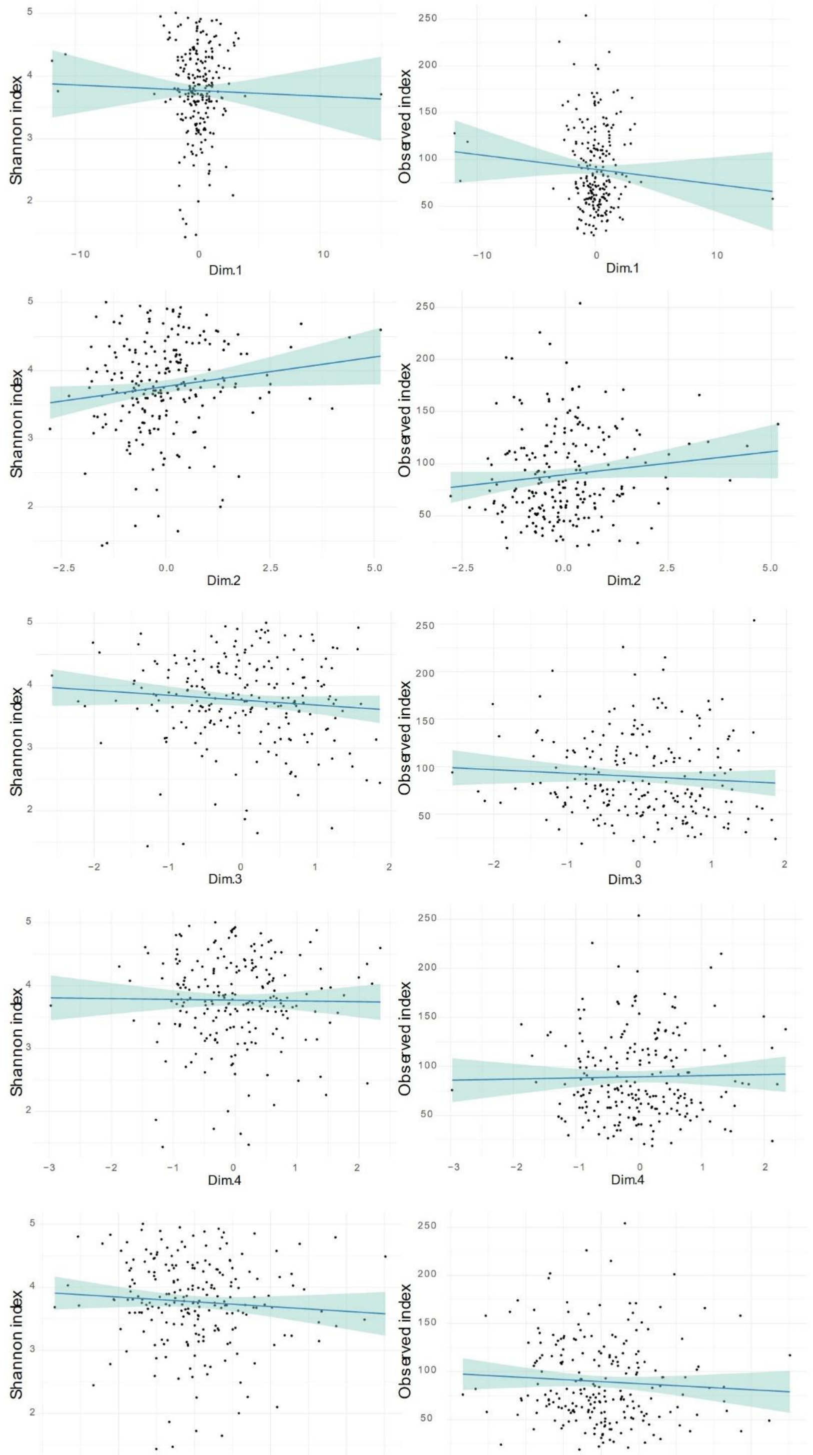


B)

| | Dim 1 | Dim2 | Dim3 | Dim4 | Dim5 |
|---|---|---|---|---|---|
| Observed | ns | *p-value* = 0.049<br>rho = 0.13 | ns | ns | ns |
| Shannon | ns | p-value = 0.019<br>rho = 0.16 | ns | ns | ns |

**Fig. S6. Correlation between mineral concentrations and bacterial α-diversity.**
In panel **A**, Spearman's rank correlation coefficient was used to evaluate correlations between each mineral dimension and the Shannon and Observed α-diversity indexes. Statistical analysis and Spearman's rho coefficients for each component are reported in panel **B**.

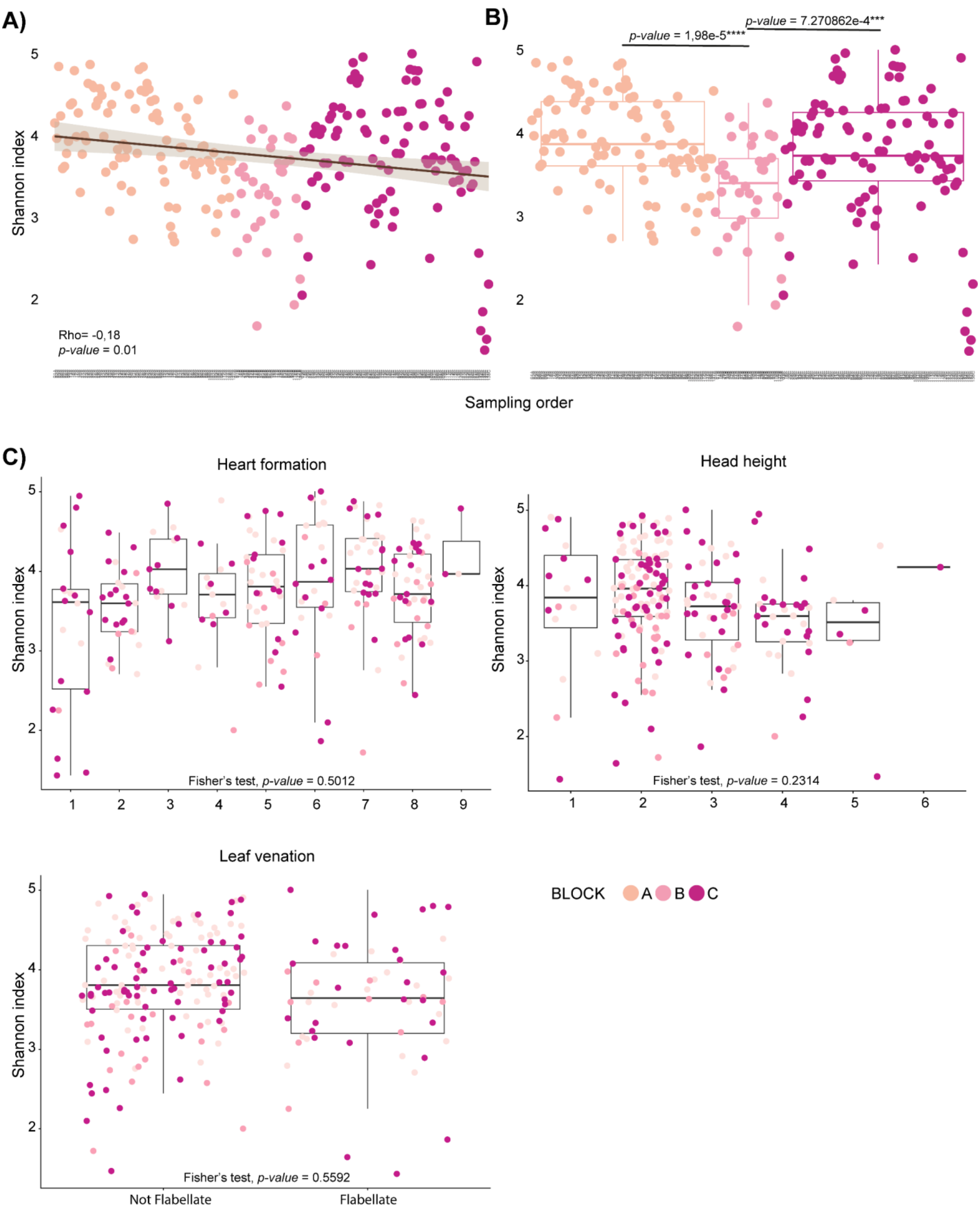


**Fig. S7. Sequential sampling influence on α-diversity.**

**A)** Variation in Shannon α-diversity based on sampling order (left side) and field block (right side). The X-axis shows sample IDs according to their sampling order, with colors representing different field blocks. In the left panel, Spearman's rank correlation, using Spearman's ρ coefficient, was applied to identify significant correlations. In the right panel, differences in Shannon diversity among field blocks were assessed using the Kruskal-Wallis test followed by Dunn's post-hoc tests. **B)** Influence of the block of origin of each sample on differences in α-diversity among the studied phenotypic traits.

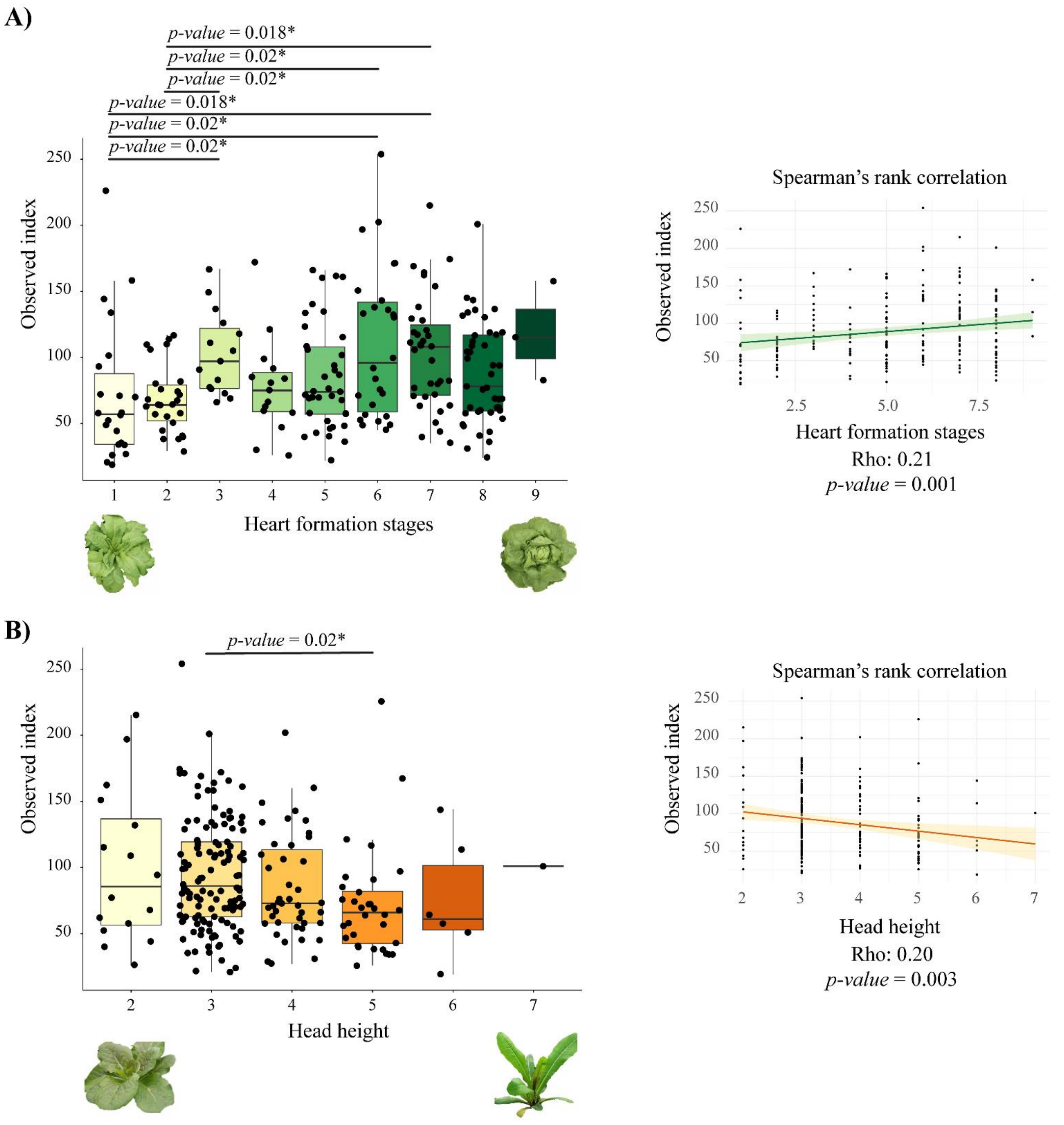


**Fig. S8. Shoot phenotypic traits strongly affect bacterial α-diversity.**

Variation in observed bacterial richness is analyzed across **A)** Heart formation stages and **B)** Head height type. Statistical differences between groups were calculated using the Kruskal-Wallis test followed by Dunn's *post-hoc* tests. Spearman's rank correlation, with Spearman's rho coefficient, was applied in panels **A** and **B** to evaluate correlations between: **A)** Different heart formation stages and **B)** Head heights.

A)

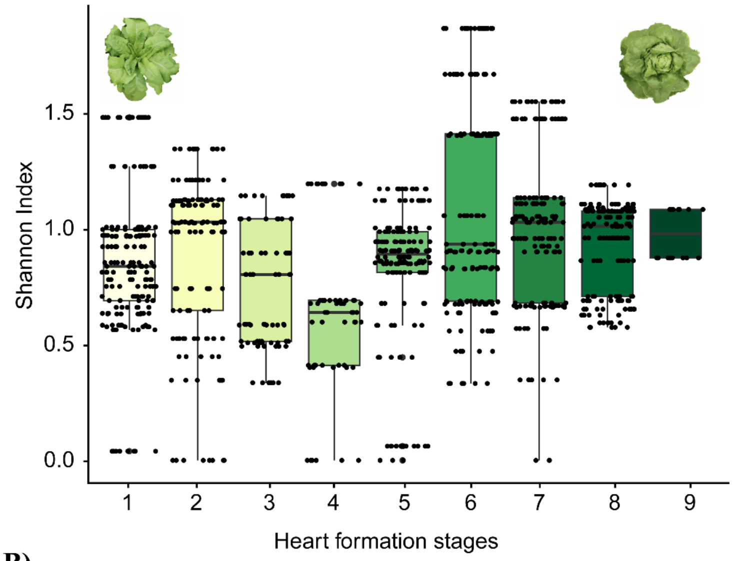


| Group 1 | Group 2 | Statistic | P-value adjusted |
|---|---|---|---|
| 1 | 3 | -2.1698 | 4.911962e-02 * |
| 1 | 2 | 2.9010 | 7.047581e-03** |
| 1 | 4 | -3.1510 | 3.660564e-03** |
| 1 | 8 | 3.2408 | 3.064612e-03** |
| 1 | 6 | 5.4365 | 3.911894e-07**** |
| 1 | 7 | 4.4771 | 2.723825e-05**** |
| 2 | 6 | 2.1975 | 4.796626e-02* |
| 2 | 5 | -2.8679 | 7.436731e-03** |
| 2 | 3 | -4.3561 | 3.970501e-05**** |
| 2 | 4 | -5.1162 | 1.402390e-06**** |
| 3 | 9 | 2.9217 | 6.961770e-03** |
| 3 | 6 | 6.3190 | 4.735986e-09**** |
| 3 | 7 | 5.5869 | 2.079862e-07**** |
| 3 | 8 | 4.6368 | 1.414723e-05**** |
| 4 | 5 | 3.0696 | 4.538545e-03** |
| 4 | 9 | 3.5038 | 1.270058e-03** |
| 4 | 6 | 6.9017 | 1.848629e-10**** |
| 4 | 7 | 6.2389 | 5.284859e-09**** |
| 4 | 8 | 5.3788 | 4.498051e-07**** |
| 5 | 8 | 3.1916 | 3.395218e-03** |
| 5 | 6 | 5.3192 | 5.357908e-07**** |
| 5 | 7 | 4.3918 | 3.677453e-05**** |

B)

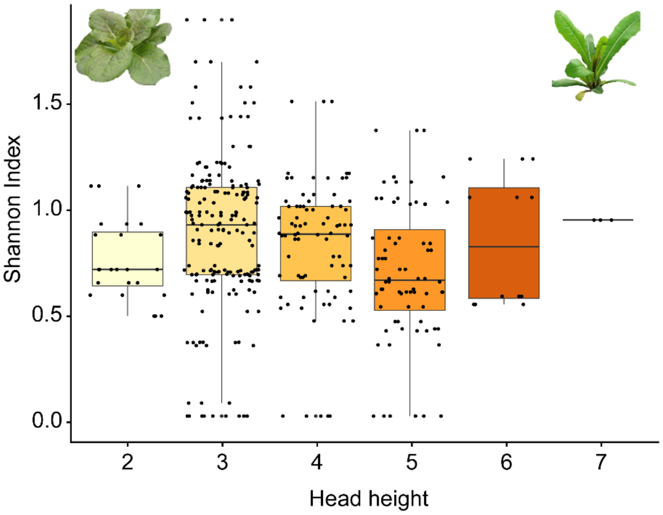


| Group 1 | Group 2 | Statistic | P-value adjusted |
|---|---|---|---|
| 2 | 4 | 3.2335 | 3.056206e-03** |
| 2 | 6 | 3.1025 | 4.111097e-03** |
| 3 | 4 | -3.3992 | 2.533755e-03** |
| 5 | 6 | 3.3121 | 2.777479e-03** |
| 2 | 3 | 5.4179 | 4.522180e-07**** |
| 3 | 5 | -8.4161 | 5.833429e-16**** |
| 4 | 5 | -4.3039 | 8.387478e-05**** |

C)

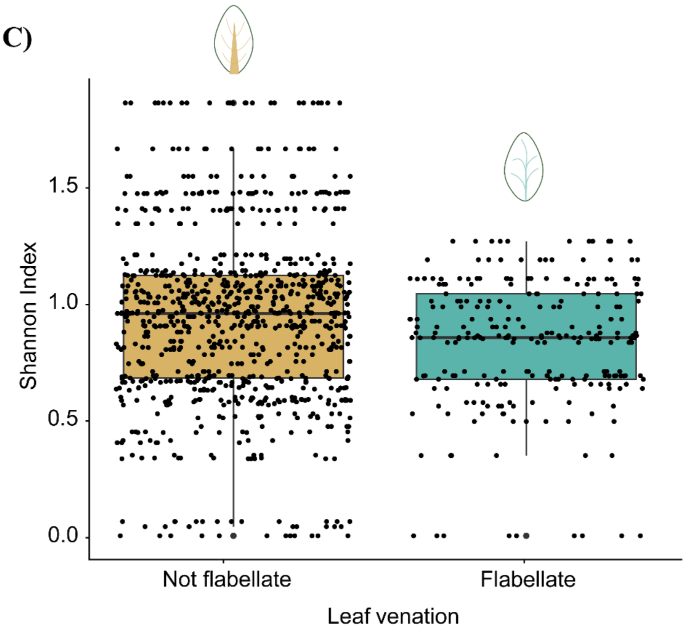


| Group 1 | Group 2 | Statistic | P-value adjusted |
|---|---|---|---|
| 1 | 2 | -3.7963 | 0.00014685*** |

**Fig. S9. Leaf metagenome-assembled genomes are differentially enriched among shoot phenotypic traits**

Variation in Shannon bacterial richness is analyzed across **A)** Heart formation stages **B)** Head height and **C)** Leaf venation type. Statistical differences between groups were calculated using the Dunn's test.

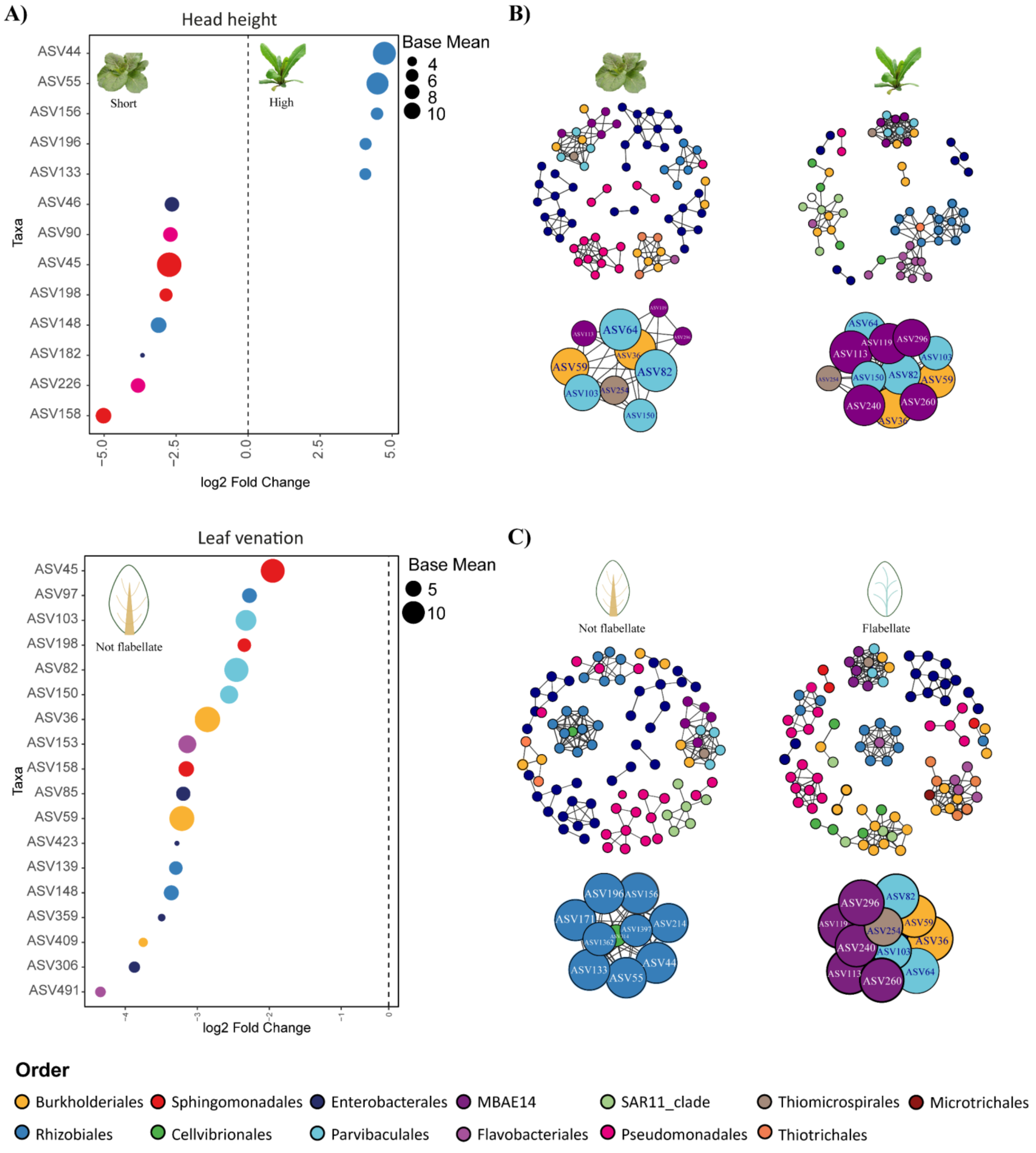


**Fig. S10. Differentially abundant ASVs between head height groups and leaf venation types.**
**A)** The upper panel shows ASVs with different relative abundances between head height Groups 3 (short plants, left side) and 5 (tall plants, right side). The lower panel represents ASVs with differing relative abundances between non-flabellate venation types (left side) and flabellate types (right side). Colors indicate the order level, as shown in the figure legend. The size of the dots reflects the base mean effect size. The community network for the two forms of each phenotypic trait is shown in panel **B)** for head height and panel **C)** for leaf venation. In each panel, the community network is displayed at the top, while hub community members are depicted at the bottom. Positive correlations are colored black, and only significant correlations (Spearman correlation > 0.65) are shown. The size of the ASVs in the hub corresponds to the hub score.

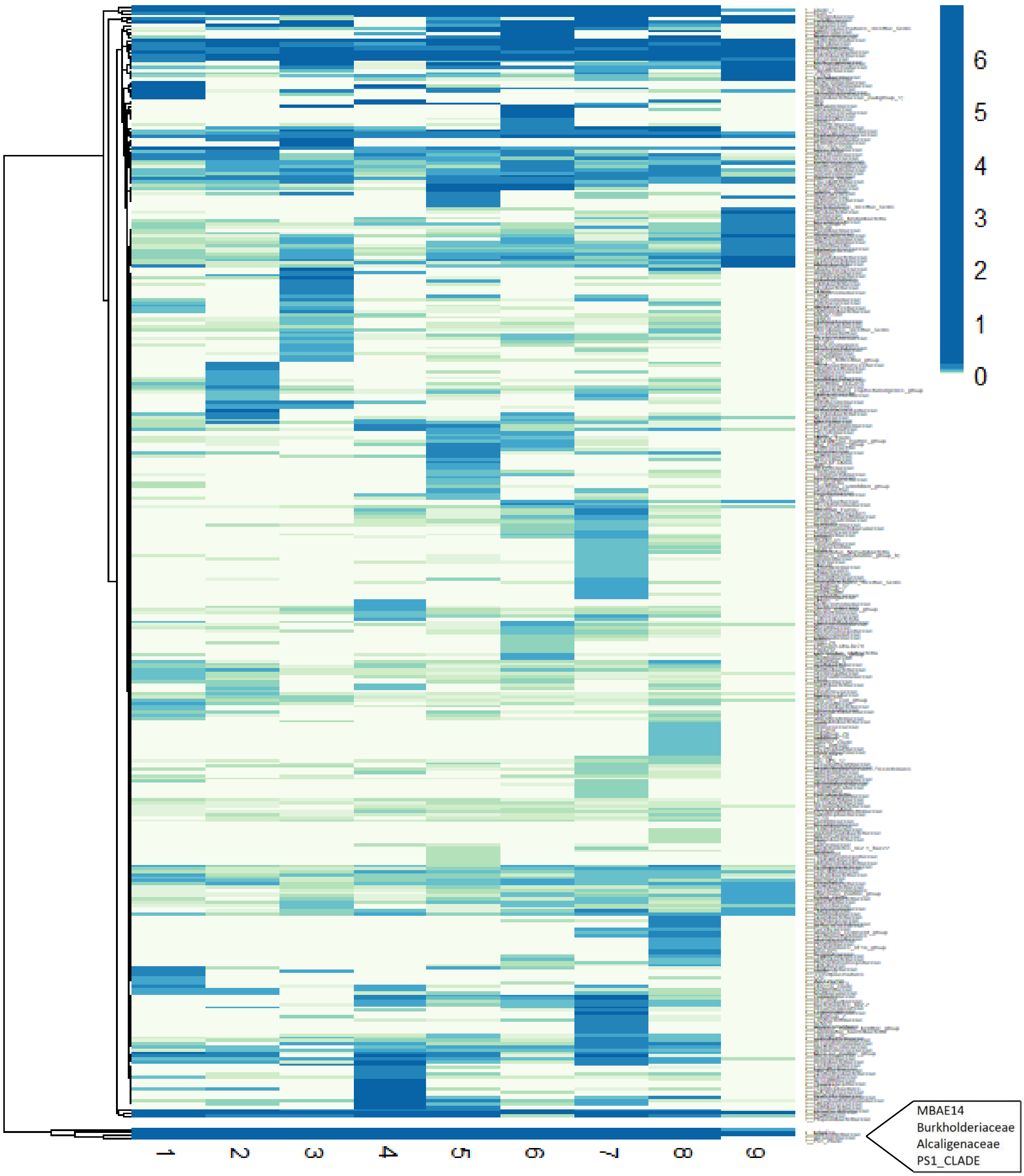


**Fig. S11. Relative abundance of leaf-associated bacterial community families across different heart formation groups.**

In the heatmap, the X-axis represents the heart formation stages, while the Y-axis shows the families, which are clustered according to their relative abundances across groups. Low relative abundances are depicted in light blue, whereas high relative abundances are indicated by dark blue.

## SUPPLEMENTARY MATERIAL AND METHODS

### Sample collection

Using a single-hole punch, we gathered three leaf disks (3 mm diameter) from each leaf, originating from three distinct areas: the central vein, the right leaf lamina, and the apical part of the leaf. Leaf disks from plants of the same genotype and grown in the same block were combined, resulting in a total of 9 leaf disks per genotype in each block and three final replicates for each genotype. Control soil samples were collected at three different time points: on March 15$^{th}$, 2022 (germination substrate), on April 21$^{st}$, 2022 (Growth soil stage 0), and on May 21$^{st}$, 2022 (Growth soil stage 1). In the latter two instances, sample collection involved pooling samples from three different points in the field (upper, median, and lower regions). Leaf samples for endophyte control were collected on May 30$^{th}$, 2022. Specifically, a leaf from the mid-upper part of the plant was collected for each genotype following the randomized design detailed in Table **S3**.

### Leaf mineral content quantification

Minerals that were poorly detected (Al, As, B, Ba, Be, Cd, Co, Cr, Cu, Li, Mo, Ni, Pb, Sb, Se, Sn) were excluded from the analysis, and only the predominantly detected ones (Ca, Fe, K, Mg, Mn, Na, P, Zn) were considered. For the latter, samples falling below the limit of quantification (<LOQ) were fit in the model, following the procedure reported in a published method (Stuart L. Beal 2001).

In brief, the LOQ was initially calculated using equation 1 (Eq.1):

Eq 1. $LOQ = \frac{\left(\frac{A(mg)\ x\ 20\ ml}{1000\ ml}\right)}{B}$

Where 'A' represents the concentration of the initial point on the linear absorbance curve (mg/L), which is specific to each mineral; '20 ml' means the volume used for sample dilution; and 'B' denotes the average weight of the leaves (0.002132 g)."

Finally, the concentration of these samples (mg/g) was calculated following equation 2 (Eq.2):

Eq 2. $[Sample] = \frac{LOQ}{\sqrt{2}}$

### DNA extraction and sequencing

To isolate endophytes, under biological hood conditions, leaves were first submerged in 70% ethanol (2 minutes), followed by immersion in 5% hypochlorite (5 minutes), and then in 70% ethanol (30 seconds). Finally, leaves were washed twice in sterile water before being homogenized with a sterile blender. The final water wash and the imprint of the leaf surface

following the sterilization procedure were plated on NB agar. As shown in Fig. S1B, no bacterial growth was observed, confirming the success of the sterilization procedure.

All DNA samples underwent MiSeq Illumina 16S amplicon sequencing at IGA Technology Services (IGATech, Udine, Italy). To summarize the process briefly, 2 × 250 bp stretches of 16S rDNA spanning the V3-V4 region were amplified using the 16S-341F (5'- CCTACGGGNBGCASCAG -3') and 16S-805R (5'- GACTACNVGGGTATCTAATCC -3') primers. During the initial amplification step, PNA clamping was applied to block the amplification of host chloroplast and mitochondrial 16S sequences, adhering to the manufacturer's protocol (PNA Bio Inc, Newbury Park, CA).

**Raw reads processing**

Forward and reverse reads were filtered and trimmed at 250 bp before merging. The merged reads underwent chimera removal and taxa assignment utilizing the Silva database (Silva database trained on the V3-V4 region, 2023). Only highly abundant Amplicon Sequence Variants (ASVs) with a relative abundance greater than 0.1% and samples with at least 100 reads were retained for subsequent analyses.

**Community composition and contributions of plant determinants to β diversity**

For a matter of visualization, the arrows in the Constrained Analysis of Principal Coordinates (CAP) were scaled down by a factor of 2.

Given the relatively minor impact of individual micronutrients and macronutrients on β diversity and the strong correlation existing among them, we chose to extract their principal components (PCs). Principal component analysis (PCA) was thus conducted in R, utilizing the Factoextra and FactoMineR packages. Only significant plant determinants were integrated into the varpart function following testing against the Bray-Curtis distance matrix.

**Integrative analysis of leaf bacterial communities: functional predictions, diversity, networks, and origins**

For the log2foldchange function application, variables were transformed into a binary format to include only those groups where significant α diversity differences were detected. Samples with a p-value $< 0.05$ were considered.

To enhance the community network representation using igraph we applied different minimum prevalence thresholds (the number of samples in which ASVs were not expressed): 6 for both heart formation groups, 10 for plants with high head heights, 4 for those with short head heights, 12 for the non-flabellate leaf venation type, and 5 for the flabellate type. Spearman's correlation coefficient was employed to assess the relationships between taxa within the

network, and only significant correlations (Spearman's correlation > 0.65) were included in the final image.

**Metagenome-assembled genome recovery and taxonomic profiling of leaf metagenomes**

A total of 137 leaf metagenomic samples were obtained from Wei et al. 2021. Sequencing reads were aligned to the reference genome of Lactuca sativa (GCA_002870075.4) using Bowtie2 (v2.5.4) with default parameters. Reads that did not align to the host genome were extracted and grouped into four clusters based on taxonomic profiles generated using MetaPhlAn4 (v4.2.0). Metagenomic assembly was performed independently for each cluster using both SPAdes (v4.2.0) and MEGAHIT (v1.2.9). Resulting assemblies were binned using four binning tools: MetaBAT2 (v2.18.1), MaxBin2 (v2.2.7), CONCOCT (v1.1.0), and VAMB (v3.0.9). All bins from each assembly strategy were subsequently dereplicated using dRep (v3.4.2) to produce a non-redundant set of metagenome-assembled genomes (MAGs). Genome quality, including completeness and contamination, was assessed using CheckM2 (v1.1.0) with default parameters. Taxonomic classification of MAGs was performed with GTDB-Tk (v2.4.1) using the GTDB database release R226. Relative abundances of the dereplicated MAGs were estimated with CoverM (v1.2.0) using default settings. Additionally, transformed abundance values were calculated as transcripts per million (TPM).

# Supplementary script

Arianna Capparotto et al

2024-08-01

```
knitr::kable(head(mtcars), "latex", longtable = TRUE)
```

### 1-Processing and clean data from QIIME2

###Denoising

```
Denoising <- read.csv("yourPath/Denoising.tsv", header = TRUE, sep = "\t", dec = ".")

Denoising
```

### 1.2 - Construct phyloseq objects

```
#libraries

library("phyloseq")
library("ggplot2")
library("readxl")
library("dplyr")
library("tibble")
library("tidyverse")
library("magrittr")


#Upload tables

##ASV Table
asv16S <- read.table(file="yourPath/16S_table-FINAL.txt",sep="\t",dec = ".",header=TRUE)
##Tax Table
tax16S <- read.table(file="yourPath/taxonomy.tsv",sep="\t",dec = ".",header=TRUE)
##Design
  design <- read.table(file="yourPath/Design.txt",sep="\t",dec = ",",header=TRUE)

##Sequences
library(Biostrings)
refseq16S <- readDNAStringSet("yourPath/dna-sequences.fasta")

#Manage data for Phyloseq
asv16S <- asv16S %>%
    tibble::column_to_rownames("OTU.ID")
tax16S <- tax16S %>%
```

```
    tibble::column_to_rownames("OTU.ID")

#Collapse Tax Table
library(stringr)
tax16S[c("Kingdom","Phylum", "Class", "Order", "Family", "Genus", "Species")]
<- str_split_fixed(tax16S$Taxon, ';', 7)
tax16S[tax16S == ""] <- NA
tax16S[tax16S == " "] <- NA
design <- design %>%
    tibble::column_to_rownames("sampleID")

otu_mat <- as.matrix(asv16S)
tax_mat <- as.matrix(tax16S)

#Create Phyloseq Object
ps16S_0 <- phyloseq(tax_table(tax_mat),
                  otu_table(otu_mat, taxa_are_rows = TRUE), sample_data(design), refseq(refseq16S))
```

### 1.3 - Mitocondria, chloroplast and Archaea Contaminations removal

```
# TAXA Calculate the number of taxa before filtering
num_taxa_before <- ntaxa(ps16S_0)


# Filtered ASV based on taxonomy
ps16S_1a <- subset_taxa(ps16S_0, !is.na(Taxon) & !Taxon %in% c("Unassigned"))
ps16S_1b <- subset_taxa(ps16S_1a, !Order %in% c(" o__Chloroplast"))
ps16S_1c <- subset_taxa(ps16S_1b, !Order %in% c("o__Mitochondria"))
ps16S_1d <- subset_taxa(ps16S_1c, !Family %in% c("f__Mitochondria"))
ps16S_1e <- subset_taxa(ps16S_1d, !Family %in% c("f__Chloroplast"))
ps16S_1f <- subset_taxa(ps16S_1e, !Genus %in% c(" g__Mitochondria"))
ps16S_1g <- subset_taxa(ps16S_1f, !Taxon %in% c("d__Eukaryota"))
ps16S_1h <- subset_taxa(ps16S_1g, !Kingdom %in% c("d__Eukaryota"))
ps16S_1i <- subset_taxa(ps16S_1h, !Kingdom %in% c("d__Archaea"))
```

### 1.4 - Remove blank samples contamination

```
################## decontam
library(here)
packageVersion("here")
# 1.0.1
library(decontam)
packageVersion("decontam")
packageVersion("decontam")
# 1.16.0
library(phyloseq)
packageVersion("phyloseq")
# 1.40.0
library(Biostrings)
packageVersion("Biostrings")
```

```r
# 2.64.0
library(tidyverse)
packageVersion("tidyverse")
# 1.3.2

d16S_leaves <- subset_samples(ps16S_1i, tissue %in% c("leaf"))
d16S_leaves <- subset_taxa(d16S_leaves, !taxa_sums(d16S_leaves) == 0)
d16S_soil <- subset_samples(ps16S_1i, tissue %in% c("soil"))
d16S_soil <- subset_taxa(d16S_soil, !taxa_sums(d16S_soil) == 0)
d16S_endophytes <- subset_samples(ps16S_1i, tissue %in% c("endophytes"))
d16S_endophytes <- subset_taxa(d16S_endophytes, !taxa_sums(d16S_endophytes) == 0)
d16S_seeds <- subset_samples(ps16S_1i, tissue %in% c("seed"))
d16S_seeds <- subset_taxa(d16S_seeds, !taxa_sums(d16S_seeds) == 0)

# import clean phyloseq from blanks
phyloseq_bianchi <- readRDS("ps_blanks.rds")

# prepare dataset
ps16S_1i.2 <- merge_phyloseq(d16S_leaves, phyloseq_bianchi)
sample_data(ps16S_1i.2)$FEAST[394:400] <- paste("blank")
base::as.data.frame(phyloseq::sample_data(ps16S_1i.2))
base::as.data.frame(phyloseq::sample_data(d16S_leaves))
view(sample_data(ps16S_1i.2))

# inspect libraries sizes

library("gtable")

df <- as.data.frame(sample_data(ps16S_1i.2))
df$LibrarySize <- sample_sums(ps16S_1i.2)
df <- df[order(df$LibrarySize), ]
df$Index <- seq(nrow(df))
df$FEAST <- as.factor(df$FEAST)
ggplot(data = df, aes(x = Index, y = LibrarySize, color = FEAST)) + geom_point() +
    theme(legend.title = element_text(size = 5), legend.text = element_text(size = 5),
        legend.key.size = unit(0.5, "cm"), legend.key.width = unit(0.5, "cm"))

# Prevalence based-decontamination

sample_data(ps16S_1i.2)$is.neg <- sample_data(ps16S_1i.2)$FEAST == "blank"

contamdf.prev <- isContaminant(ps16S_1i.2, method = "prevalence", neg = "is.neg")
table(contamdf.prev$contaminant)

# Make phyloseq object of presence-absence in negative controls and true
# samples
ps.pa <- transform_sample_counts(ps16S_1i.2, function(abund) 1 * (abund > 0))
ps.pa.neg <- prune_samples(sample_data(ps.pa)$FEAST == "blank", ps.pa)
ps.pa.pos <- prune_samples(sample_data(ps.pa)$FEAST == "leaf", ps.pa)
# Make data.frame of prevalence in positive and negative samples
df.pa <- data.frame(pa.pos = taxa_sums(ps.pa.pos), pa.neg = taxa_sums(ps.pa.neg),
    contaminant = contamdf.prev$contaminant)
ggplot(data = df.pa, aes(x = pa.neg, y = pa.pos, color = contaminant)) + geom_point() +
```

```
    xlab("Prevalence (Negative Controls)") + ylab("Prevalence (True Samples)")

# trim contaminants

ps.noncontam <- prune_taxa(!contamdf.prev$contaminant, ps16S_1i.2)
ps.noncontam

ps16S_1i.3 <- merge_phyloseq(ps.noncontam, d16S_endophytes, d16S_seeds, d16S_soil)
view(otu_table(ps16S_1i.3))

ps16S_1i.3 = subset_samples(ps16S_1i.3, FEAST != "blank")
```

### 1.5 - Create final tables resuming the process

```
num_taxa_after1 <- data.frame(Before_filtering = ntaxa(ps16S_0), Remove_Unassigned = ntaxa(ps16S_1a),
    remove_chloroplast = ntaxa(ps16S_1e), remove_mithocondria = ntaxa(ps16S_1f),
    remove_Eukaryota = ntaxa(ps16S_1h), remove_Archaea = ntaxa(ps16S_1i), romove_blanks = ntaxa(ps16S_1i

# READS Before after filtering Calculate the total number of reads per sample
total_reads <- colSums(otu_table(ps16S_0))
total_reads_Unassigned <- colSums(otu_table(ps16S_1a))
total_reads_Chloro <- colSums(otu_table(ps16S_1e))
total_reads_Mito <- colSums(otu_table(ps16S_1f))
total_reads_Eukaria <- colSums(otu_table(ps16S_1h))
total_reads_Archaea <- colSums(otu_table(ps16S_1i))
total_reads_blanks <- colSums(otu_table(ps16S_1i.3))


# Gather
total_filtering_reads <- cbind(row.names(total_reads), total_reads, total_reads_Unassigned,
    total_reads_Chloro, total_reads_Mito, total_reads_Eukaria, total_reads_Archaea,
    total_reads_blanks)
total_filtering_reads_f <- merge(total_filtering_reads, design, by = "row.names")
write.csv(total_filtering_reads_f, file = "total_filtering_reads_f.csv", row.names = FALSE)
write.csv(total_filtering_reads, file = "total_filtering_reads.csv", row.names = FALSE)


# Create a data frame with sample IDs and total reads
reads_per_sample_table <- data.frame(SampleID = sample_names(ps16S_1i.3), TotalReads = total_reads,
    TotalReads_Unassigned = total_reads_Unassigned, TotalReads_Bacterial = total_reads_Chloro,
    total_reads_Eukaria = total_reads_Eukaria, total_reads_blanks = total_reads_blanks,
    Percent_Bacteria = 100 * total_reads_blanks/total_reads)
```

### 1.6 - Remove samples with less than 100 counts and set a relative abundance

```
# Rename ASV
dna.16S <- taxa_names(ps16S_1i.3)
names(dna.16S) <- taxa_names(ps16S_1i.3)
ps16S_2 <- merge_phyloseq(ps16S_1i.3, dna.16S)
```

```r
taxa_names(ps16S_2) <- paste0("ASV", seq(ntaxa(ps16S_2)))

# Subset Samples (remove sample with less than 100 counts)
ps16S_3 <- prune_samples(sample_sums(ps16S_2) >= 100, ps16S_2)
ps16S_4 <- filter_taxa(ps16S_3, function(x) sum(x) > 0, TRUE)

# Filter ASV (keep ASV > 0.1% RA per sample)
ps16S_5 <- transform_sample_counts(ps16S_4, function(x) ifelse((x/sum(x)) >= 0.001,
    x, 0))
ps16S_6 <- filter_taxa(ps16S_5, function(x) sum(x) > 0, TRUE)
otu_table16S <- data.frame(otu_table(ps16S_6))
ps16S_6_w <- ps16S_6

saveRDS(ps16S_5, "ps16S_less_clean_noblanks.rds")
saveRDS(ps16S_6, "ps16S_clean_noblanks.rds")
```

### 1.7 - phylogenetic tree

```r
# create phylogenetic tree Neighbor-Joining tree were constructed the packages
# DECIPHER v 2.12.0 and Phangorn v 2.5.5 using default paramaters.

library("DECIPHER")
packageVersion("DECIPHER")

seqs.16S <- refseq(ps16S_6)
alignment.16S <- AlignSeqs(DNAStringSet(seqs.16S), anchor = NA)
library("phangorn")
packageVersion("phangorn")
phang.align.16S <- phyDat(as(alignment.16S, "matrix"), type = "DNA")
dm.16S <- dist.ml(phang.align.16S)  #measure of distances
treeNJ.16S <- NJ(dm.16S)  # Note, tip order != sequence order
fit.16S <- pml(treeNJ.16S, data = phang.align.16S)
fit.GTR.16S <- update(fit.16S, k = 4, inv = 0.2)
fit.GTR.16S <- optim.pml(fit.GTR.16S, model = "GTR", optInv = TRUE, optGamma = TRUE,
    rearrangement = "stochastic", control = pml.control(trace = 0))
d16S.tree <- phyloseq(tax_table(ps16S_6), sample_data(ps16S_6), otu_table(ps16S_6,
    taxa_are_rows = FALSE), refseq(ps16S_6), phy_tree(treeNJ.16S))
saveRDS(d16S.tree, "16S_PS_clean_tree.rds")


# import phylogenetic tree
library(ape)
treeNJ.16S <- readRDS("treeNJ.16S.decontam.rds")

# join the phylogenetic tree

ps16S_6 <- phyloseq(tax_table(ps16S_6), sample_data(ps16S_6), otu_table(ps16S_6),
    refseq(ps16S_6), phy_tree(treeNJ.16S))

write.tree(phy_tree(ps16S_6), "output.tree.noblanks")

saveRDS(ps16S_6, "ps16S_clean_noblanks_withtree.rds")
```

### 1.8 - Size dependent rarefaction for compartment

```
# create a dataset for each compartment

# leaf
d16S_filtered_leaves <- subset_samples(ps16S_6_w, tissue %in% c("leaf"))
d16S_filtered_leaves <- subset_taxa(d16S_filtered_leaves, !taxa_sums(d16S_filtered_leaves) ==
    0)

# seed
d16S_filtered_seed <- subset_samples(ps16S_6_w, tissue %in% c("seed"))
d16S_filtered_seed <- subset_taxa(d16S_filtered_seed, !taxa_sums(d16S_filtered_seed) ==
    0)

# soil
d16S_filtered_soil <- subset_samples(ps16S_6_w, tissue %in% c("soil"))
d16S_filtered_soil <- subset_taxa(d16S_filtered_soil, !taxa_sums(d16S_filtered_soil) ==
    0)

# endophytes
d16S_filtered_endophyte <- subset_samples(ps16S_6_w, tissue %in% c("endophytes"))
d16S_filtered_endophyte <- subset_taxa(d16S_filtered_endophyte, !taxa_sums(d16S_filtered_endophyte) ==
    0)

# rarefy each compartment

library("ranacapa")

r16S_leaf <- rarefy_even_depth(d16S_filtered_leaves, sample.size = 1000, rngseed = 900)

r16S_soil <- rarefy_even_depth(d16S_filtered_soil, sample.size = 14000, rngseed = 900)

r16S_seeds <- rarefy_even_depth(d16S_filtered_seed, sample.size = 200, rngseed = 900)

r16S_endophytes <- rarefy_even_depth(d16S_filtered_endophyte, sample.size = 4500,
    rngseed = 900)

# create the merged phyloseq object with each rarefied compartment

phy_tree <- readRDS("treeNJ.16S.decontam.rds")
merged_phylo <- merge_phyloseq(r16S_leaf, r16S_soil, r16S_seeds, r16S_endophytes)
vector_asv <- row.names(otu_table(merged_phylo))
vector_sample <- row.names(sample_data(merged_phylo))
phy_tree_filtered <- prune_taxa(vector_asv, phy_tree)
merged_phylo_tree <- phyloseq(tax_table(merged_phylo), sample_data(merged_phylo),
    otu_table(merged_phylo), refseq(merged_phylo), phy_tree(phy_tree))

# visualize the output of rarefaction

rcurve16S <- ggrare(merged_phylo_tree, color = "tissue", step = 999, se = FALSE) +
    labs(title = "16S rRNA gene")
rcurve16S
```

```
rcurve16Sf_rarefied <- rcurve16S + theme(strip.background = element_rect(color = "white",
    fill = "#585858", size = 1, linetype = "solid"), strip.text.x = element_text(size = 12,
    color = "white"), axis.title.x = element_text(size = 12, face = "bold"), axis.title.y = element_tex
    face = "bold"), plot.title = element_text(size = 16), axis.text.x = element_text(face = "bold"),
    axis.text.y = element_text(face = "bold")) + ylab("Species richness") + xlab("Sample size") +
    ggtitle("16S rRNA gene") + facet_grid(~tissue, scale = "free")

rcurve16Sf_rarefied
```

***2-Community composition*** # 2- Community composition ### 2.1 - Figure 2A

```
library(ggplot2)

#The most abundnat members of the community (Relative abundance >0.1%)
# Prepare the data
data <- data.frame(
  Phylum = c("Proteobacteria", "Bacteroidota", "Firmicutes", "Actinobacteriota", "Unassigned",
             "Planctomycetota", "Verrucomicrobiota", "Desulfobacterota", "Campilobacterota",
             "Acidobacteriota", "Chloroflexi", "Cyanobacteria", "Bdellovibrionota", "Patescibacteria"),
  Percentage = c(71.31652174, 8.385217391, 6.978695652, 5.0765217390,3.416956522, 1.089130435, 1.065217


my_colors <- c("#E41A1C", "#377EB8", "#4DAF4A", "#984EA3", "#FF7F00",
               "#FFFF33", "#A65628", "#F781BF", "#999999", "#66C2A5",
               "#FC8D62", "#8DA0CB", "#E78AC3", "#A6D854")

# Reorder the levels of Phylum based on their abundance
data$Phylum <- factor(data$Phylum, levels = data$Phylum[order(data$Percentage, decreasing = T)])

# Create the stacked bar plot
stacked_ggp <- ggplot(data, aes(x = "", y = Percentage, fill = Phylum, group = Phylum)) +
  geom_bar(stat = 'identity', position = 'stack', width = 1) +
  scale_fill_manual(values = my_colors) +
  guides(fill = guide_legend(title = "Factors Explaining Variation")) +
  labs(title = "Stacked Bar Plot of Phylum Percentages") +
  theme_bw() +
  theme(panel.border = element_blank(),
        panel.grid.major = element_blank(),
        panel.grid.minor = element_blank(),
        legend.position = "right")

stacked_ggp
```

### 2.2 - Figure 2B- - Statistical analysis to find taxa differences between varieties and groups of closely related genotypes

```
#To do this part, the community threshold (relative abundance >0.1%) has been recalculated using only l

ps16S_3<-subset_samples(ps16S_6_w, tissue %in% c("leaf"))
ps16S_4 <- filter_taxa(ps16S_3, function(x) sum(x) > 0, TRUE)
```

```
#And a new phyloseq object (r16S_leaf) have been created with the same procedure reported above (Part 1

#Obtain a table made of OTU + TAXA table to calculate the relative abundance at each single taxonomic r

#convergence table
taxa.table_1<-tax_table(r16S_leaf)
taxa.table_1<-as.data.frame(taxa.table_1)
otu.table_1<-as.data.frame(otu_table(r16S_leaf))
convergence_table_1<- (cbind(taxa.table_1[4:9], otu.table_1))
write.csv(convergence_table_1, "convergence_table_1.csv")
write.csv(samples, "sample_data_leaf.csv")

#Then, create the tables (one for each taxonomic rank) by following these instructions:

  #1) Open the Excel file and create a pivot table from the converged table by clicking on "Recommended

  #2) Select all samples.

  #3) Copy the table into a new Excel file, transposing the entire table.

  #4) Calculate the relative abundance using the formula:

  #Relative abundance = Count in each cell/ Sum of all counts per sample

  #5) Ensure that the sum of the relative abundances for each sample equals 1.

  #6) Save the table as .csv file.

#Import data and perform statistical analysis by running the following code:

#libraries
library(ggExtra)
library(multcomp)
library(ggpubr)
library(rstatix)
library(patchwork)
library(tidyverse)

#Import dataset - change every time the table imported at the taxonomic levels you want to perform stat
  data <- read.csv("C:/yourPath/species.csv", #phylum, order, class, family, genus
                header=T,sep = ";", na.string=c("na", "NA", "null"))

#convert the table to have your taxa in a single column
data %>%
 pivot_longer(., cols = (2:294),  names_to= "species" , values_to = "Abundance")-> data2

#tranform to factor
data2$genotype<-as.factor(data2$genotype)
data2$species <- as.factor(data2$species) #change with the taxa you are examining
data2$variety <- as.factor(data2$variety)
data2$genetic.distance <- as.factor(data2$genetic.distance)

#Statistic - change "species" with the taxa you are examining and "genetic distance" with the factor fo
```

```
#Krukall Wallis test
x <- unique(data2$species)
models_genetic.distance  <- sapply(x, function(My){
  kruskal.test(Abundance~ genetic.distance, data=data2, species== My)
}, simplify=FALSE)
models_genetic.distance

table_genetic.distance  <- do.call(rbind.data.frame, models_genetic.distance )
table_genetic.distance

#Dunn Post hoc test
data2 %>%
  group_by(species) %>%
  dunn_test(Abundance~ genetic.distance, p.adjust.method = "fdr") -> dunns_species_genetic.distance

write.csv(dunns_species_genetic.distance, "dunns_species_genetic.distance.csv")

#Now move to phyton3 and GraPhlAn graphical interface.
    #1 - form the table " "dunns_species_genetic.distance" select all the taxa for which there is signi
    #2 - Create a table with unique significant taxa names
    #3 - Create figure 2B-C by following the script of Segata et al., 2015
```

**2.3 - Figure S2A- Bacterial phylum community composition (relative abundance >0.1%) across different samples.**

```
library(ggplot2)
library(vegan)
library(dplyr)
library(scales)
library(grid)
library(reshape2)
library(phyloseq)

merged_phylo_tree <- subset_samples(merged_phylo_tree, !(genotype2 %in% c("11", "71", "119", "92")))

#leaf
r16S_L <- subset_samples(merged_phylo_tree, tissue %in% c("leaf"))
r16S_L <- subset_taxa(r16S_L, !taxa_sums(r16S_L) == 0)
#seed
r16S_seed <- subset_samples(merged_phylo_tree, tissue %in% c("seed"))
r16S_seed <- subset_taxa(r16S_seed, !taxa_sums(r16S_seed) == 0)
#endophytes
r16S_endophytes <- subset_samples(merged_phylo_tree, FEAST %in% c("endophytes"))
r16S_endophytes <- subset_taxa(r16S_endophytes, !taxa_sums(r16S_endophytes) == 0)
#soilstage0
r16S_soil0 <- subset_samples(merged_phylo_tree, FEAST %in% c("soil1"))
r16S_soil0 <- subset_taxa(r16S_soil0, !taxa_sums(r16S_soil0) == 0)
#soilstage1
r16S_soil1 <- subset_samples(merged_phylo_tree, FEAST %in% c("soil2"))
r16S_soil1 <- subset_taxa(r16S_soil1, !taxa_sums(r16S_soil1) == 0)
#germination soil
```

```r
r16S_soil_germ <- subset_samples(merged_phylo_tree, FEAST %in% c("soil 3"))
r16S_soil_germ <- subset_taxa(r16S_soil_germ, !taxa_sums(r16S_soil_germ) == 0)


#agglomerate at the desired taxonomic ranck
erie_phylum <- r16S_L %>%              #Change with the desired compartment
  tax_glom(taxrank = "Phylum") %>%
  transform_sample_counts(function(x) {x/sum(x)} ) %>%
  psmelt() %>%
  arrange(Phylum)


phylum_colors <- c(
  " p__Acidobacteriota" = "#CBD588",
  " p__Actinobacteriota" = "#5F7FC7",
  " p__Bacteroidota" = "orange",
  " p__Bdellovibrionota" = "#DA5724",
  " p__Campilobacterota" = "#508578",
  " p__Chloroflexi" = "#CD9BCD",
  " p__Cyanobacteria" = "#00FFFF",
  " p__Deinococcota" = "#8A7C64",
  " p__Desulfobacterota" = "#FFC0CB",
  " p__Dependentiae" = "#0000CD",
  " p__Elusimicrobiota" = "#D1A33D",
  " p__Entotheonellaeota" = "#1E90FF",
  " p__Fibrobacterota" = "#FF0000",
  " p__Fusobacteriota" = "#800080",
  " p__Firmicutes" = "#00FF00",
  " p__Hydrogenedentes" = "#5E738F",
  " p__Gemmatimonadota" = "#AD6F3B",
  " p__Latescibacterota" = "#ADFF2F",
  " p__Methylomirabilota" = "#40E0D0",
  " p__Myxococcota" = "#673770",
  " p__Modulibacteria" = "#B71C1C",
  " p__Nitrospirota"= "#FFFF00",
  " p__NB1-j" = "#A5D6A7",
  " p__Patescibacteria" = "#D14285",
  " p__Planctomycetota" = "#652926",
  " p__Proteobacteria" = "#FFD700",
  " p__Verrucomicrobiota" = "#8569D5",
  " p__WS2" = "#FF00FF",
  " p__SAR324_clade(Marine_group_B)" = "#90CAF9",
  " p__Sva0485" = "#FBC02D",
  " p__Spirochaetota" ="#F44336",
  " p__Synergistota" = "#008000"
)


################ 1- seeds

erie_phylum$Phylum<-as.factor(erie_phylum$Phylum)
erie_phylum$replicate<-as.factor(erie_phylum$replicate)
```

```r
# Plot
plot <- ggplot(erie_phylum, aes(x = replicate, y = Abundance, fill = Phylum)) +
  geom_bar(stat = "identity") +
  scale_fill_manual(values = phylum_colors) + personalizzati
  theme_minimal() +
  theme(
    axis.title.x = element_text(),
    axis.title.y = element_text(size = 11, face = "bold"),
    plot.title = element_text(size = 12, face = "bold"),
    axis.text.x = element_text(angle = 45, hjust = 1, size = 7)
  ) +
  guides(fill = guide_legend(reverse = FALSE, keywidth = 1, keyheight = 1)) +
  ylab("Relative Abundance (Phyla > 0.01%) \n") +
  ggtitle("Seed bacterial community")

##########2- endophytes

erie_phylum$Phylum<-as.factor(erie_phylum$Phylum)
erie_phylum$replicate<-as.factor(erie_phylum$replicate)

# Plot
plot <- ggplot(erie_phylum, aes(x = replicate, y = Abundance, fill = Phylum)) +
  geom_bar(stat = "identity") +
  scale_fill_manual(values = phylum_colors) +  personalizzati
  theme_minimal() +
  theme(
    axis.title.x = element_text(),
    axis.title.y = element_text(size = 11, face = "bold"),
    plot.title = element_text(size = 12, face = "bold"),
    axis.text.x = element_text(angle = 45, hjust = 1, size = 7)
  ) +
  guides(fill = guide_legend(reverse = FALSE, keywidth = 1, keyheight = 1)) +
  ylab("Relative Abundance (Phyla > 0.01%) \n") +
  ggtitle("Endophytic bacterial community")

##########3- germination soil

erie_phylum$Phylum<-as.factor(erie_phylum$Phylum)
erie_phylum$replicate<-as.factor(erie_phylum$replicate)

# Plot
plot <- ggplot(erie_phylum, aes(x = replicate, y = Abundance, fill = Phylum)) +
  geom_bar(stat = "identity") +
  scale_fill_manual(values = phylum_colors) +
  theme_minimal() +
  theme(
    axis.title.x = element_text(),
    axis.title.y = element_text(size = 11, face = "bold"),
    plot.title = element_text(size = 12, face = "bold"),
    axis.text.x = element_text(angle = 45, hjust = 1, size = 7)
  ) +
  guides(fill = guide_legend(reverse = FALSE, keywidth = 1, keyheight = 1)) +
  ylab("Relative Abundance (Phyla > 0.01%) \n") +
```

```
  ggtitle("Endophytic bacterial community")


##########4- Growth soil stage 0

erie_phylum$Phylum<-as.factor(erie_phylum$Phylum)
erie_phylum$replicate<-as.factor(erie_phylum$replicate)
erie_phylum$block<-as.factor(erie_phylum$block)

# Plot
plot <- ggplot(erie_phylum, aes(x = replicate, y = Abundance, fill = Phylum)) +
   facet_grid( ~ block, scales = 'free') +
  geom_bar(stat = "identity") +
  scale_fill_manual(values = phylum_colors) +
  theme_minimal() +
  theme(
    axis.title.x = element_text(),
    axis.title.y = element_text(size = 11, face = "bold"),
    plot.title = element_text(size = 12, face = "bold"),
    axis.text.x = element_text(angle = 45, hjust = 1, size = 7)
  ) +
  guides(fill = guide_legend(reverse = FALSE, keywidth = 1, keyheight = 1)) +
  ylab("Relative Abundance (Phyla > 0.01%) \n") +
  ggtitle("Endophytic bacterial community")

##########4- Growth soil stage 1

erie_phylum$Phylum<-as.factor(erie_phylum$Phylum)
erie_phylum$replicate<-as.factor(erie_phylum$replicate)
erie_phylum$block<-as.factor(erie_phylum$block)

# Plot
plot <- ggplot(erie_phylum, aes(x = replicate, y = Abundance, fill = Phylum)) +
   facet_grid( ~ block, scales = 'free') +
  geom_bar(stat = "identity") +
  scale_fill_manual(values = phylum_colors) +
  theme_minimal() +
  theme(
    axis.title.x = element_text(),
    axis.title.y = element_text(size = 11, face = "bold"),
    plot.title = element_text(size = 12, face = "bold"),
    axis.text.x = element_text(angle = 45, hjust = 1, size = 7)
  ) +
  guides(fill = guide_legend(reverse = FALSE, keywidth = 1, keyheight = 1)) +
  ylab("Relative Abundance (Phyla > 0.01%) \n") +
  ggtitle("Endophytic bacterial community")

##########5- Leaves

erie_phylum$Phylum<-as.factor(erie_phylum$Phylum)
erie_phylum$sampleID3<-as.factor(erie_phylum$sampleID3)
erie_phylum$block<-as.factor(erie_phylum$block)
```

```
# Plot
plot <- ggplot(erie_phylum, aes(x = sampleID3, y = Abundance, fill = Phylum)) +
   facet_grid( ~ block, scales = 'free') +
  geom_bar(stat = "identity") +
  scale_fill_manual(values = phylum_colors) +
  theme_minimal() +
  theme(
    axis.title.x = element_text(),
    axis.title.y = element_text(size = 11, face = "bold"),
    plot.title = element_text(size = 12, face = "bold"),
    axis.text.x = element_text(angle = 45, hjust = 1, size = 7),
      legend.position = "right",
    legend.box.spacing = unit(0.5, "lines")
  ) +
  guides(fill = guide_legend(reverse = FALSE, keywidth = 1, keyheight = 1, ncol=1)) +
  ylab("Relative Abundance (Phyla > 0.01%) \n") +
  ggtitle("Leaf bacterial community")
```

###2.4 - Table S9

```
erie_abundance <- r16S_L %>% #changed with the desired compartment
  tax_glom(taxrank = "Phylum") %>%
  transform_sample_counts(function(x) {x / sum(x)* 100}) %>%
  psmelt() %>%
  arrange(Phylum)

erie_abundance <- erie_abundance %>%
  group_by(Phylum) %>%
  summarize(asv_count = n())

erie_abundance <- erie_abundance %>%
  mutate(total_asvs = sum(asv_count)) %>%
  mutate(relative_abundance = asv_count / total_asvs * 100) %>%
  ungroup()

top_phyla <- erie_abundance %>%
  group_by(Phylum) %>%
  summarize(mean_relative_abundance = mean(relative_abundance)) %>%
  arrange(desc(mean_relative_abundance))
```

**2.5 - Figure S2B- Bray-Curtis dissimilarity distance matrix illustrating the beta diversity across the different sample types.**

```
bray.pcoa.16S_s <-ordinate(merged_phylo_tree,"PCoA","bray")



i.bray.pcoa.16S <- plot_ordination(merged_phylo_tree,
                                   bray.pcoa.16S_s,
                                   type = "samples",
                                   color = "FEAST",
```

```
                                    label = "Unique.ID") +
  geom_point(aes(fill = FEAST, color = FEAST),
             size = 5,
             shape = 21,
             stroke = 1.5,
             alpha = 1) +
  ggtitle("16S rRNA - Bray") +
  theme_minimal() +
  theme(panel.background = element_rect(fill = "white")) +
  scale_color_manual(values = c(
    "leaf" = "#b7e1cd",
    "soil2" = "#e59864",
    "soil1" = "#f1cd79",
    "soil 3" = "#f6e4a9",
    "endophytes" = "#a97b36",
    "seed" = "#fef1e0"
  )) +
  scale_fill_manual(values = c(
    "leaf" = "#b7e1cd",
    "soil2" = "#e59864",
    "soil1" = "#f1cd79",
    "soil 3" = "#f6e4a9",
    "endophytes" = "#a97b36",
    "seed" = "#fef1e0"
  )) +
  scale_color_manual(values = c(
    "leaf" = "#99c7b3",
    "soil2" = "#d88b49",
    "soil1" = "#d2b86b",
    "soil 3" = "#e3d093",
    "endophytes" = "#8c5c2a",
    "seed" = "#f1d7b3"
  ))
```

***3-Beta diversity analysis*** # 3- Beta diversity analysis ### 3.1 - Figures 2D-E

```
#library
library(vegan)

#Create a subset of the phyloseq object to include only leaf samples
r16S_L <- subset_samples(merged_phylo_tree, tissue %in% c("leaf"))
r16S_L <- subset_taxa(r16S_L, !taxa_sums(r16S_L) == 0)
r16S_L= subset_samples(r16S_L, genotype2 != "11") #no in nature tree
r16S_L= subset_samples(r16S_L, genotype2 != "71") #no belonging to genetic group
r16S_L= subset_samples(r16S_L, genotype2 != "119") #no belonging to genetic group
r16S_L= subset_samples(r16S_L, genotype2 != "92") #Too much Na values

#PERMANOVA statistical analysis (Adonis2)

bray.16S <- phyloseq::distance(r16S_L, method = "bray")
mdat.16S_bray <- as(sample_data(r16S_L), "data.frame")

adonis.16STiss_bray <- adonis2(bray.16S ~ genetic.distance, #change to variety
```

```r
                                data = mdat.16S_bray, perm = 999)
print(adonis.16STiss_bray)

#Figure 2E-F - Constrained Analysis of Principal Coordinates (CAP)

Cap_cluster<- capscale(bray.16S~genetic.distance,#change to variety
                      data = mdat.16S_bray, add=FALSE, subset, na.action = na.omit)

##ANOVA-like permutation analysis
perm_test <- anova(Cap_cluster, permutations = 999)
p_value <- perm_test$Pr[1]
p_value

##Run variability table function
variability_table <- function(cca){

        chi <- c(cca$tot.chi,
                       cca$CCA$tot.chi, cca$CA$tot.chi)
        variability_table <- cbind(chi, chi/chi[1])
        colnames(variability_table) <- c("inertia", "proportion")
        rownames(variability_table) <- c("total", "constrained", "unconstrained")
        return(variability_table)
}


##Generate variability table
var_tbl <- variability_table(Cap_cluster)
ci <- quantile(perm_test$F.perm, c(.05,.95))*perm_test$chi[1]/var_tbl["total", "inertia"]
eig_CCA <- Cap_cluster$CCA$eig
eig_CA <- Cap_cluster$CA$eig
variance <- var_tbl["constrained", "proportion"]
variance_NMDS <- var_tbl["unconstrained", "proportion"]

##Extract unweighted and  unweighted CCA values and unconstrained CA values (here you basically extract

###Extract colnames
colnames <- c(paste("u", colnames(Cap_cluster$CCA$u)[1], sep=""), paste("u", colnames(Cap_cluster$CCA$u

 ### Extract datapoints
points = data.frame(c(Cap_cluster$CCA$u[, 1]),
                    c(Cap_cluster$CCA$u[, 2]),
                    c(Cap_cluster$CCA$w[, 1]),
                    c(Cap_cluster$CCA$w[, 2]),
                    c(Cap_cluster$CA$u[, 1]),
                    c(Cap_cluster$CA$u[, 2]))
colnames(points) <- colnames
rownames(points)


##Calculate axis explained variation
eig_tot <- sum(Cap_cluster$CCA$eig)
eigenvalues <- Cap_cluster$CCA$eig/eig_tot
eigenvalues
```

```r
##Bind with metadata


df <- cbind(points, mdat.16S_bray[match(rownames(points), rownames(mdat.16S_bray)),])


#Extract Arrows

#script for extracting genetic distance arrows
arrow <- data.frame(Cap_cluster$CCA$biplot)
Cap_cluster2<- capscale(bray.16S ~ genetic.distance-1,  data = mdat.16S_bray, add=FALSE,na.action=na.exc
genetic.distance1<- data.frame(Cap_cluster2$CCA$biplot)[1,]
arrows2 <- rbind(arrow,genetic.distance1)
new_row_names <- sub("genetic.distance", "", row.names(arrows2))
row.names(arrows2)<-new_row_names
arrows2$names <- row.names(arrows2)


#script for extracting variety arrows
Cap_cluster3<- capscale(bray.16S ~ variety-1,  data = mdat.16S_bray, add=FALSE,na.action=na.exclude , su
varietyButterhead<- data.frame(Cap_cluster3$CCA$biplot)[1,]
arrows2 <- rbind(arrow,varietyButterhead)
new_row_names <- sub("variety", "", row.names(arrows2))
row.names(arrows2)<-new_row_names

#Colored palette

#genetic distance
colors <- c("#d73027","#91bfdb", "#fc8d59","#4575b4", "#fee090","#e0f3f8")

#Variety
colors_var<- c( "#fde0ef","#a1d76a","#c51b7d","#4d9221","#e9a3c9","#013220","#e6f5d0")


#Plot
pwCPOA <- ggplot(df, aes(x=wCAP1, y=wCAP2)) +
  geom_point(aes(fill=genetic.distance), shape=22, size=3.5, na.value = "grey50") +
  scale_fill_manual(values = colors) + #colors_var for variery
  labs(
    x = paste("wCPCoA 1 (", format(100 * eig_CCA[1] / sum(eig_CCA), digits=4), "%)", sep=""),
    y = paste("wCPCoA 2 (", format(100 * eig_CCA[2] / sum(eig_CCA), digits=4), "%)", sep="")
  ) +
  ggtitle(paste(format(100 * variance, digits=3), " % of variance; p=", format(p_value, digits=2), sep=
  theme(
    legend.position = "right",
    panel.grid.major = element_line(color = "#EDEDED"),
    panel.grid.minor = element_line(color = "#EDEDED"),
    panel.background = element_rect(fill = "white"),
    plot.background = element_rect(fill = "white")
  ) +
  geom_segment(data = arrows2, aes(x = 0, y = 0, xend = CAP1, yend = CAP2), size = 0.5,
               arrows2 = arrow(type = "closed", length = unit(0.2, "inches"))) +
  geom_text(data = arrows2, aes(x = CAP1, y = CAP2, label = row.names(arrows2)), vjust = -1.5, hjust = 
```

```
pwCPOA
```

### 3.2 - Figures 3A and S5A-E

```
#libraries
library("FactoMineR")
library("factoextra")

#transform to factor and numeric
mdat.16S_bray$variety<-as.factor(mdat.16S_bray$variety)
mdat.16S_bray$genetic.distance<-as.factor(mdat.16S_bray$genetic.distance)
mdat.16S_bray$block<-as.factor(mdat.16S_bray$block)
mdat.16S_bray$P<-as.numeric(mdat.16S_bray$P)
mdat.16S_bray$Ca<-as.numeric(mdat.16S_bray$Ca)
mdat.16S_bray$Fe<-as.numeric(mdat.16S_bray$Fe)
mdat.16S_bray$K<-as.numeric(mdat.16S_bray$K)
mdat.16S_bray$Mg<-as.numeric(mdat.16S_bray$Mg)
mdat.16S_bray$Mn<-as.numeric(mdat.16S_bray$Mn)
mdat.16S_bray$Na<-as.numeric(mdat.16S_bray$Na)
mdat.16S_bray$Zn<-as.numeric(mdat.16S_bray$Zn)

#adjust the table with the right row.names
row.names(mdat.16S_bray)<-NULL
row.names(mdat.16S_bray)<- mdat.16S_bray$sampleID3
view(mdat.16S_bray)

# log transform mineral data
mdat.16S_bray_active <- log(mdat.16S_bray[20:27])

#Extract principal components
res.pca <- PCA(mdat.16S_bray_active, graph = FALSE)
print(res.pca)
var <- get_pca_ind(res.pca)
var<-as.data.frame(var$coord)

mineral_content<-var[, c("Dim.2", "Dim.4", "Dim.5"), drop = FALSE] #to use in varpart

#Figure S5A- correlation matrix among micro- and macronutrients

library(gplots)
cor_matrix <- cor(mdat.16S_bray_active, use = "complete.obs")

#color palette
default_colors <- colorRampPalette(c("#053061", "#2166AC", "#4393C3", "#92C5DE", "#D1E5F0",
                                     "#FFFFFF", "#FDDBC7", "#F4A582", "#D6604D", "#B2182B",
                                     "#67001F"))(200)

#plot
corrplot(cor_matrix, type = "upper", order = "hclust", col=default_colors,
         tl.col = "black", tl.srt = 45)
```

```r
#Figure 3A - visualize Correlations between variables and dimensions
#library
library("corrplot")

default_colors <- colorRampPalette(c("#053061", "#2166AC", "#4393C3", "#92C5DE", "#D1E5F0",
                                     "#FFFFFF", "#FDDBC7", "#F4A582", "#D6604D", "#B2182B",
                                     "#67001F"))(200)

corrplot(res.pca$var$cor, is.corr=FALSE, col=default_colors, tl.cex=0.8, cl.cex=0.8, addrect=2)

#Figure S5B

fviz_pca_biplot(res.pca,
                axes = c(1,2),
                legend.title = "Mineral variation",
                col.var = "black",
                pointshape = 19,
                pointsize = 2.5,
                labelsize = 3,
                label = "var",
                title = "Mineral variation"
)

#Figure S5B
fviz_pca_biplot(res.pca,
                axes = c(2,4),
                legend.title = "Mineral variation",
                col.var = "black",
                pointshape = 19,
                pointsize = 2.5,
                labelsize = 3,
                label = "var",
                title = "Mineral variation"
)


##############Fig. S5E
### percentage contribution of each variable to every principal component (PC) obtained from the PCA.
res.pca <- PCA(mdat.16S_bray_active, graph = FALSE)
percent_contribution <- res.pca$var$contrib
```

### 3.3 - Figures 3B, S5D, and Table S12

```r
#add mineral content coordinates to mdat.16S_bray table
mdat.16S_bray <- as(sample_data(r16S_L), "data.frame")
mdat.16S_bray$sampleID  <- rownames(mdat.16S_bray)
rownames(mdat.16S_bray) <- NULL
mdat.16S_bray <- cbind(sampleID = mdat.16S_bray$sampleID, mdat.16S_bray[, -ncol(mdat.16S_bray)])
mdat.16S_bray$sample_ID <- NULL
sampleID3_values <- rownames(mdat.16S_bray)
```

```
var <- var %>% mutate(sampleID3 = rownames(var))
mdat.16S_bray <- left_join(mdat.16S_bray, var, by = "sampleID3")
rownames(mdat.16S_bray) <- mdat.16S_bray[,1]

#Tranfrom mineral into numeric
mdat.16S_bray$P<-as.numeric(mdat.16S_bray$P)
mdat.16S_bray$Ca<-as.numeric(mdat.16S_bray$Ca)
mdat.16S_bray$Fe<-as.numeric(mdat.16S_bray$Fe)
mdat.16S_bray$K<-as.numeric(mdat.16S_bray$K)
mdat.16S_bray$Mg<-as.numeric(mdat.16S_bray$Mg)
mdat.16S_bray$Mn<-as.numeric(mdat.16S_bray$Mn)
mdat.16S_bray$Na<-as.numeric(mdat.16S_bray$Na)
mdat.16S_bray$Zn<-as.numeric(mdat.16S_bray$Zn)
mdat.16S_bray$Dim.1<-as.numeric(mdat.16S_bray$Dim.1)
mdat.16S_bray$Dim.2<-as.numeric(mdat.16S_bray$Dim.2)
mdat.16S_bray$Dim.3<-as.numeric(mdat.16S_bray$Dim.3)
mdat.16S_bray$Dim.4<-as.numeric(mdat.16S_bray$Dim.4)
mdat.16S_bray$Dim.5<-as.numeric(mdat.16S_bray$Dim.5)

#Table S12 - PERMANOVA (Adonis2) to test significance of dimensions

adonis.16Sb_phenotypic_traits <- adonis2(bray.16S~Dim.2, #Change dimension
                                data = mdat.16S_bray, perm = 999, na.action=na.exclude )
adonis.16Sb_phenotypic_traits

#Figure S5D - nested PERMANOVA (Adonis 2)
adonis.16Sb_phenotypic_traits <- adonis2(bray.16S~genetic.distance/Fe, #Change genetic distance with va
                                data = mdat.16S_bray, perm = 999, na.action=na.exclude )
adonis.16Sb_phenotypic_traits

#Figure 3B - Constrained Analysis of Principal Coordinates (CAP)

Cap_cluster<- capscale(bray.16S~Dim.2+Dim.4+Dim.5,#Only significant dimensions
                       data = mdat.16S_bray, add=FALSE, subset, na.action = na.omit)

##ANOVA-like permutation analysis
perm_test <- anova(Cap_cluster, permutations = 999)
p_value <- perm_test$Pr[1]
p_value

##Run variability table function
variability_table <- function(cca){

        chi <- c(cca$tot.chi,
                       cca$CCA$tot.chi, cca$CA$tot.chi)
        variability_table <- cbind(chi, chi/chi[1])
        colnames(variability_table) <- c("inertia", "proportion")
        rownames(variability_table) <- c("total", "constrained", "unconstrained")
        return(variability_table)
}


##Generate variability table
```

```
var_tbl <- variability_table(Cap_cluster)
ci <- quantile(perm_test$F.perm, c(.05,.95))*perm_test$chi[1]/var_tbl["total", "inertia"]
eig_CCA <- Cap_cluster$CCA$eig
eig_CA <- Cap_cluster$CA$eig
variance <- var_tbl["constrained", "proportion"]
variance_NMDS <- var_tbl["unconstrained", "proportion"]

##Extract unweighted and  unweighted CCA values and unconstrained CA values (here you basically extract

###Extract colnames
colnames <- c(paste("u", colnames(Cap_cluster$CCA$u)[1], sep=""), paste("u", colnames(Cap_cluster$CCA$u

 ### Extract datapoints
points = data.frame(c(Cap_cluster$CCA$u[, 1]),
                    c(Cap_cluster$CCA$u[, 2]),
                    c(Cap_cluster$CCA$w[, 1]),
                    c(Cap_cluster$CCA$w[, 2]),
                    c(Cap_cluster$CA$u[, 1]),
                    c(Cap_cluster$CA$u[, 2]))
colnames(points) <- colnames
rownames(points)


##Calculate axis explained variation
eig_tot <- sum(Cap_cluster$CCA$eig)
eigenvalues <- Cap_cluster$CCA$eig/eig_tot
eigenvalues

##Bind with metadata


df <- cbind(points, mdat.16S_bray[match(rownames(points), rownames(mdat.16S_bray)),])


#Extract Arrows

#script for extracting genetic distance arrows
arrow <- data.frame(Cap_cluster$CCA$biplot)

#Plot
pwCPOA <- ggplot(df, aes(x=wCAP1, y=wCAP2)) +
  geom_point(aes(fill=genetic.distance), shape=22, size=3.5, na.value = "grey50") +
  scale_fill_manual(values = colors_var) +
  labs(
    x = paste("wCPCoA 1 (", format(100 * eig_CCA[1] / sum(eig_CCA), digits=4), "%)", sep=""),
    y = paste("wCPCoA 2 (", format(100 * eig_CCA[2] / sum(eig_CCA), digits=4), "%)", sep="")
  ) +
  ggtitle(paste(format(100 * variance, digits=3), " % of variance; p=", format(p_value, digits=2), sep=
  theme(
    legend.position = "right",
    panel.grid.major = element_line(color = "#EDEDED"),
    panel.grid.minor = element_line(color = "#EDEDED"),
    panel.background = element_rect(fill = "white"),
```

```
    plot.background = element_rect(fill = "white")
  ) +
  geom_segment(data = arrow, aes(x = 0, y = 0, xend = CAP1, yend = CAP2), size = 0.5,
               arrow = arrow(type = "closed", length = unit(0.2, "inches"))) +
  geom_text(data = arrow, aes(x = CAP1, y = CAP2, label = row.names(arrow)), vjust = -1.5, hjust = NA)


pwCPOA
```

#### 3.4 - impute missing values using mice package.

```
library(mice, warn.conflicts = FALSE)
library(VIM)

# import Design
  design_MICE <- read.table(file="your path/Design - not imputed.txt",sep="\t",dec = ",",header=TRUE)

design_MICE <- design_MICE[1:131, c(3, 26:42)]

#Visualize missing data
md.pattern(design_MICE)
aggr_plot <- aggr(design_MICE, col=c('navyblue','red'), numbers=TRUE, sortVars=TRUE, labels=names(data)

#perform imputation

tempData <- mice(design_MICE,m=5,maxit=50,meth='pmm',seed=500)
summary(tempData)

Design <- complete(tempData,1)

#save it and import it in the next chunck
```

#### 3.5 - Figure 4

```
#the imputed dataset have been used to perform phenotypic traits analysis

#Figure 4
Cap_cluster<- capscale(bray.16S~Heart.formation+Head.height+Head.shape+Leaf.venation+Head.leafs.overlap
                       data = mdat.16S_bray, add=FALSE, subset, na.action = na.omit)

##ANOVA-like permutation analysis
perm_test <- anova(Cap_cluster, permutations = 999)
p_value <- perm_test$Pr[1]
p_value

##Run variability table function
variability_table <- function(cca){

        chi <- c(cca$tot.chi,
```

```
                       cca$CCA$tot.chi, cca$CA$tot.chi)
        variability_table <- cbind(chi, chi/chi[1])
        colnames(variability_table) <- c("inertia", "proportion")
        rownames(variability_table) <- c("total", "constrained", "unconstrained")
        return(variability_table)
}


##Generate variability table
var_tbl <- variability_table(Cap_cluster)
ci <- quantile(perm_test$F.perm, c(.05,.95))*perm_test$chi[1]/var_tbl["total", "inertia"]
eig_CCA <- Cap_cluster$CCA$eig
eig_CA <- Cap_cluster$CA$eig
variance <- var_tbl["constrained", "proportion"]
variance_NMDS <- var_tbl["unconstrained", "proportion"]

##Extract unweighted and  unweighted CCA values and unconstrained CA values (here you basically extract

###Extract colnames
colnames <- c(paste("u", colnames(Cap_cluster$CCA$u)[1], sep=""), paste("u", colnames(Cap_cluster$CCA$u

 ### Extract datapoints
points = data.frame(c(Cap_cluster$CCA$u[, 1]),
                    c(Cap_cluster$CCA$u[, 2]),
                    c(Cap_cluster$CCA$w[, 1]),
                    c(Cap_cluster$CCA$w[, 2]),
                    c(Cap_cluster$CA$u[, 1]),
                    c(Cap_cluster$CA$u[, 2]))
colnames(points) <- colnames
rownames(points)


##Calculate axis explained variation
eig_tot <- sum(Cap_cluster$CCA$eig)
eigenvalues <- Cap_cluster$CCA$eig/eig_tot
eigenvalues

##Bind with metadata


df <- cbind(points, mdat.16S_bray[match(rownames(points), rownames(mdat.16S_bray)),])


#Extract Arrows

#script for extracting genetic distance arrows
arrow <- data.frame(Cap_cluster$CCA$biplot)

#Plot
pwCPOA <- ggplot(df, aes(x=wCAP1, y=wCAP2)) +
  geom_point(aes(fill=genetic.distance), shape=22, size=3.5, na.value = "grey50") +
  scale_fill_manual(values = colors_var) +
  labs(
```

```
    x = paste("wCPCoA 1 (", format(100 * eig_CCA[1] / sum(eig_CCA), digits=4), "%)", sep=""),
    y = paste("wCPCoA 2 (", format(100 * eig_CCA[2] / sum(eig_CCA), digits=4), "%)", sep="")
  ) +
  ggtitle(paste(format(100 * variance, digits=3), " % of variance; p=", format(p_value, digits=2), sep=
  theme(
    legend.position = "right",
    panel.grid.major = element_line(color = "#EDEDED"),
    panel.grid.minor = element_line(color = "#EDEDED"),
    panel.background = element_rect(fill = "white"),
    plot.background = element_rect(fill = "white")
  ) +
  geom_segment(data = arrow, aes(x = 0, y = 0, xend = CAP1, yend = CAP2), size = 0.5,
               arrow = arrow(type = "closed", length = unit(0.2, "inches"))) +
  geom_text(data = arrow, aes(x = CAP1, y = CAP2, label = row.names(arrow)), vjust = -1.5, hjust = NA)


pwCPOA
```

***4 - Alpha diversity analysis*** # 4- Alpha diversity analysis ### 4.1 - Create a table with Shannon and Observed indexes

```
#r16S
#merged_phylo_tree
#r16S_raref
#r16S_L

#subset
r16S_L <- subset_samples(merged_phylo_tree, tissue %in% c("leaf"))
r16S_L= subset_samples(r16S_L, genotype2 != "11") #no tree coordinated
r16S_L= subset_samples(r16S_L, genotype2 != "71") #no belonging to genetic group
r16S_L= subset_samples(r16S_L, genotype2 != "119") #no belonging to genetic group
r16S_L= subset_samples(r16S_L, genotype2 != "92") #too much NA values

library(picante)
#Table of alpha-diversity estimators
table_r16S <- estimate_richness(r16S_L, split = TRUE, measures=c("Observed", "InvSimpson","Shannon"))

#Bind design + table of alpha_div
sdr16S <- sample_data(r16S_L)
datar16S <- cbind(sample_data(sdr16S), table_r16S)

#add phylogenetic info to table
rgyrBPD <- as.data.frame.matrix(otu_table(r16S_L))
PD_matrix <- pd(samp = t(rgyrBPD),tree = phy_tree(r16S_L), include.root = F)

#Bind design + table of alpha_div
datar16S <- merge(datar16S, PD_matrix, by="row.names", all=TRUE)
```

#### 4.2 - Figures S4 - Variation in alpha diversity between varieties and groups of closely related genotypes

```
#libraries
library(ggplot2)
library(tidyverse)
library(ggpubr)
library(rstatix)
library(tidyverse)
library(ggthemes)
library(multcompView)
library(agricolae)
library(dplyr)

#transforma as factor
datar16S$sampleID3 <- as.factor(datar16S$sampleID3)
datar16S$genotype2<-as.factor(datar16S$genetic.distance) #these are groups of closely related genotypes
datar16S$variety<- as.factor(datar16S$variety)
datar16S$block<-as.factor(datar16S$block)

#Figure S4B - variety

#Observed index
#Add Dunn comparisons to the graph
res_obs<-datar16S %>%
  dunn_test(Observed ~ variety, p.adjust.method = "fdr")

stat.test <- res_obs %>% add_xy_position(x = "variety")


ggplot(datar16S, aes(variety, Observed),  na.rm = FALSE) +
  geom_boxplot(aes(fill = variety), show.legend = TRUE) +
  geom_jitter(size=1)+
  labs(x="variety", y="Observed ASV richness") +
  scale_fill_manual(values = colors_var)+
  theme_bw() +
  theme(panel.grid.major = element_blank(), panel.grid.minor = element_blank())+
  stat_pvalue_manual(stat.test, hide.ns = TRUE)->plot_16S_Observed_b

plot_16S_Observed_b

#Shannon index
res_obs<-datar16S %>%
  dunn_test(Shannon ~ variety, p.adjust.method = "fdr")

stat.test <- res_obs %>% add_xy_position(x = "variety")


ggplot(datar16S, aes(variety, Shannon),  na.rm = FALSE) +
  geom_boxplot(aes(fill = variety), show.legend = TRUE) +
  geom_jitter(size=1)+
  labs(x="variety", y="Shannon ASV richness") +
  scale_fill_manual(values = colors_var)+
```

```
  theme_bw() +
  theme(panel.grid.major = element_blank(), panel.grid.minor = element_blank())+
  stat_pvalue_manual(stat.test, hide.ns = TRUE)->plot_16S_Shannon_b

plot_16S_Shannon_b

##Figure S4A - genetic.distance

#Observed index
#Add Dunn comparisons to the graph
res_obs<-datar16S %>%
  dunn_test(Observed ~ genetic.distance, p.adjust.method = "fdr")

stat.test <- res_obs %>% add_xy_position(x = "genetic.distance")


ggplot(datar16S, aes(genetic.distance, Observed),  na.rm = FALSE) +
  geom_boxplot(aes(fill = genetic.distance), show.legend = TRUE) +
  geom_jitter(size=1)+
  labs(x="genetic.distance", y="Observed ASV richness") +
  scale_fill_manual(values = colors_var)+
  theme_bw() +
  theme(panel.grid.major = element_blank(), panel.grid.minor = element_blank())+
  stat_pvalue_manual(stat.test, hide.ns = TRUE)->plot_16S_Observed_b

plot_16S_Observed_b

#Shannon index
res_obs<-datar16S %>%
  dunn_test(Shannon ~ genetic.distance, p.adjust.method = "fdr")

stat.test <- res_obs %>% add_xy_position(x = "genetic.distance")


ggplot(datar16S, aes(genetic.distance, Shannon),  na.rm = FALSE) +
  geom_boxplot(aes(fill = genetic.distance), show.legend = TRUE) +
  geom_jitter(size=1)+
  labs(x="genetic.distance", y="Shannon ASV richness") +
  scale_fill_manual(values = colors_var)+
  theme_bw() +
  theme(panel.grid.major = element_blank(), panel.grid.minor = element_blank())+
  stat_pvalue_manual(stat.test, hide.ns = TRUE)->plot_16S_Shannon_b

plot_16S_Shannon_b
```

### 4.3 - Figures 6 and S8

```
#transform phenotypic traits to factors

datar16S$Seedling.cotyledon.shape<-as.factor(datar16S$Seedling.cotyledon.shape)
datar16S$Head.height<-as.factor(datar16S$Head.height)
```

```
datar16S$Seedling.anthocyanin.presence<-as.factor(datar16S$Seedling.anthocyanin.presence)
datar16S$Leaf.shape.cultivated.material<-as.factor(datar16S$Leaf.shape.cultivated.material)
datar16S$Leaf.blistering<-as.factor(datar16S$Leaf.blistering)
datar16S$Leaf.margin.undulation<-as.factor(datar16S$Leaf.margin.undulation)
datar16S$Leaf.venation<-as.factor(datar16S$Leaf.venation)
datar16S$Leaf.division<-as.factor(datar16S$Leaf.division)
datar16S$Leaf.color<-as.factor(datar16S$Leaf.division)
datar16S$Leaf.color.intensity<-as.factor(datar16S$Leaf.color.intensity)
datar16S$Leaf.anthocyanin.content<-as.factor(datar16S$Leaf.anthocyanin.content)
datar16S$Side.shoot.formation.tendency<-as.factor(datar16S$Side.shoot.formation.tendency)
datar16S$Tipburn.sensitivity<-as.factor(datar16S$Tipburn.sensitivity)
datar16S$Plant.diameter<-as.factor(datar16S$Plant.diameter)
datar16S$Head.shape<-as.factor(datar16S$Head.shape)
datar16S$Head.leafs.overlap<-as.factor(datar16S$Head.leafs.overlap)
datar16S$Head.height<-as.factor(datar16S$Head.height)
datar16S$Heart.formation<-as.factor(datar16S$Heart.formation)
datar16S$Observed<-as.numeric(datar16S$Observed)
datar16S$Shannon<-as.numeric(datar16S$Shannon)


#Calculate statistical differences in alpha-diversity inside each phenotypical traits

datar16S %>%
 pivot_longer(., cols=29:45, names_to= "trait", values_to = "values") -> data_phen

library(ggplot2)

#Shannon index differences
res_Shannon<-data_phen %>%
  group_by(trait) %>%
  dunn_test(Shannon ~ values, p.adjust.method = "fdr")
stat.test <- res_Shannon %>% add_xy_position(x = "trait")

#Observed index differences
res_obs<-data_phen %>%
  group_by(trait) %>%
  dunn_test(Observed ~ values, p.adjust.method = "fdr")
stat.test <- res_obs %>% add_xy_position(x = "trait")

#Visualize alpha-diversity plot

#Figure 6A- Shannon diversity between heart formation groups

Heart.palette <- c("#ffffe5", "#f7fcb9", "#d9f0a3", "#addd8e", "#78c679", "#41ab5d", "#238443", "#00683

gg_traits <- datar16S %>%
  ggplot(aes(x = Heart.formation, y = Shannon, fill = Heart.formation)) +
  geom_boxplot(na.rm = TRUE) +  # Ignora i NA nel boxplot
  geom_jitter(na.rm = TRUE) +
  theme_classic() +
  scale_fill_manual(values = Heart.palette)

gg_traits
```

```r
#Figure S8A- Observed diversity between heart formation groups
gg_traits <- datar16S %>%
  ggplot(aes(x = Heart.formation, y = Observed, fill = Heart.formation)) +
  geom_boxplot(na.rm = TRUE) +  # Ignora i NA nel boxplot
  geom_jitter(na.rm = TRUE) +
  theme_classic() +
  scale_fill_manual(values = Heart.palette)
gg_traits

#Figure 6B- Shannon diversity between Head height groups

Height.palette <- c("#ffffd4", "#fee391", "#fec44f", "#fe9929", "#d95f0e", "#993404")


gg_traits <- datar16S %>%
  ggplot(aes(x = Head.height, y = Shannon, fill = Head.height)) +
  geom_boxplot(na.rm = TRUE) +
  geom_jitter(na.rm = TRUE) +
  theme_classic() +
  scale_fill_manual(values = Height.palette)

#Figure S8B- Observed diversity between head height groups
gg_traits <- datar16S %>%
  ggplot(aes(x = Head.height, y = Observed, fill = Head.height)) +
  geom_boxplot(na.rm = TRUE) +
  geom_jitter(na.rm = TRUE) +
  theme_classic() +
  scale_fill_manual(values = Height.palette)

#Figure 5C- Shannon diversity between Leaf venation groups

#palette
colors <- c("#d8b36580", "#5ab4ac80")
dark_colors <- c("#d8b365", "#5ab4ac")

# Violin plot leaf venation Shannon
gg_traits <- datar16S %>%
  ggplot(aes(x = Leaf.venation, y = Shannon)) +
  geom_violin(aes(fill = Leaf.venation), na.rm = TRUE, alpha = 0.2) +
  geom_boxplot(aes(fill = Leaf.venation), width = 0.1, na.rm = TRUE, fill = "white", color = "black") +
  geom_jitter(aes(fill = Leaf.venation, color = Leaf.venation), width = 0.2, size = 3, shape = 21, strol
  theme_classic() +
  scale_fill_manual(values = colors) +
  scale_color_manual(values = dark_colors) +
  guides(fill = FALSE)
gg_traits
```

#### 4.4 - Figures 6A-B and S8A-B- Spearman correlations between phenotypic traits and alpha-diversity indexes

```r
library(ggplot2)
library(Kendall)

#transform to factor
datar16S$Head.height<-as.numeric(datar16S$Head.height)
datar16S$Heart.formation<-as.numeric(datar16S$Heart.formation)

#Figure 6A- spearman's Rho correlation coefficient for heart formation and Shannon index

res<-cor.test(datar16S$Heart.formation, datar16S$Shannon, method ="spearman")
print(res)

#scatter plot
ggplot(datar16S, aes(x = Heart.formation, y = Shannon)) +
  geom_point() +  # Color of the points
  geom_smooth(method = "lm", color = "#006837", fill = "#addd8e")
  labs(x = "Heart formation", y = "Shannon index") +
  theme_minimal()

#Figure S8A- spearman's Rho correlation coefficient for heart formation and Observed index

res<-cor.test(datar16S$Heart.formation, datar16S$Observed, method ="spearman")
print(res)

#scatter plot
ggplot(datar16S, aes(x = Heart.formation, y = Observed)) +
  geom_point() +  # Color of the points
  geom_smooth(method = "lm", color = "#006837", fill = "#addd8e") +
  labs(x = "Heart formation", y = "Observed index") +
  theme_minimal()

#Figure 6B- spearman's Rho correlation coefficient for Head height and Shannon index

res<-cor.test(datar16S$Head.height, datar16S$Shannon, method ="spearman")
print(res)

#scatter plot
ggplot(datar16S, aes(x = Head.height, y = Shannon)) +
  geom_point() +
  geom_smooth(method = "lm", color = "#d95f0e", fill = "#fee391")
  labs(x = "Heart formation", y = "Shannon index") +
  theme_minimal()

#Figure S8B- spearman's Rho correlation coefficient for Head height and Observed index

res<-cor.test(datar16S$Head.height, datar16S$Observed, method ="spearman")
print(res)

#scatter plot
ggplot(datar16S, aes(x = Head.height, y = Observed)) +
  geom_point() +
  geom_smooth(method = "lm", color = "#d95f0e", fill = "#fee391")+
  labs(x = "Heart formation", y = "Observed index") +
```

```
  theme_minimal()
```

### 4.5 - Figure S7

```
#Custom X-axis samples so that they are ordered according to the sampling order

#Function
custom_order <- function(x) {
  num <- as.numeric(str_extract(x, "\\d+"))
  letter <- str_extract(x, "[A-Za-z]")
  temp_df <- data.frame(sampleID3 = x, num = num, letter = letter)
  temp_df <- temp_df %>% arrange(letter, num)
  temp_df$new_index <- 1:nrow(temp_df)
  return(temp_df)
}

#Apply function and order samples (sampleID3)
ordered_data <- custom_order(datar16S$sampleID3)
datar16S <- datar16S %>%
  left_join(ordered_data, by = c("sampleID3" = "sampleID3"))

#Calculate the Spearman coefficient
res <- cor.test(datar16S$new_index, datar16S$Shannon, method = "spearman")
print(res)

#color palette
block_colors <- c(
  "A" = "#fde0dd",
  "B" = "#fa9fb5",
  "C" = "#c51b8a"
)

#Figure S9A- Scatter plot

gg_traits <- ggplot(datar16S, aes(x = new_index, y = Shannon, color = block)) +
  geom_point(size=6) +
  geom_smooth(method = "lm", color = "#5B3A29", fill = "#BFAE9A", se = TRUE) +
  labs(x = "Sampling order", y = "Shannon index") +
  scale_x_continuous(breaks = ordered_data$new_index, labels = ordered_data$sampleID3) +
  scale_color_manual(values = block_colors) +
  theme_minimal() +
  theme(
    axis.text.x = element_text(angle = 90, hjust = 1, vjust = 0.5, size = 5),
    panel.grid.major = element_blank(),
    panel.grid.minor = element_blank(),
    panel.border = element_blank()
  )

#Figure S9B - Box plot

gg_traits <- ggplot(datar16S, aes(x = new_index, y = Shannon, color = block)) +
  geom_boxplot() +
```

```
  geom_point(size = 4) +  # Points colored by block parameter
  labs(x = "Sampling order", y = "Shannon index") +
  scale_x_continuous(breaks = ordered_data$new_index, labels = ordered_data$sampleID3) +  # Maintain or
  scale_color_manual(values = block_colors) +  # Assign colors to blocks
  theme_minimal() +
  theme(
    axis.text.x = element_text(angle = 90, hjust = 1, vjust = 0.5, size = 5),  # Rotate x-axis text
    panel.grid.major = element_blank(),
    panel.grid.minor = element_blank(),
    panel.border = element_blank()
  )

gg_traits

res_obs<-datar16S %>%
  dunn_test(Shannon ~ block, p.adjust.method = "fdr")

#Figure S9C

#Shannon by blocks
gg_traits <- datar16S %>%
  ggplot(aes(x = Heart.formation, y = Shannon)) + #change Heart.formation with Head.height or Leaf.vena
  geom_boxplot() +
  geom_jitter(aes(color = block), size = 3, na.rm = TRUE) +  # Points colored by block
  theme_classic() +
  scale_color_manual(values = block_colors)
```

#### 4.6 - Figure S6A

```
#bind micro/macro nutrients' coordinates to alpha-diversity table
datar16S$sampleID  <- rownames(datar16S)
rownames(datar16S) <- NULL
datar16S <- cbind(sampleID = datar16S$sampleID, datar16S[, -ncol(datar16S)])
datar16S$sample_ID <- NULL
sampleID3_values <- rownames(datar16S)
var <- var %>% mutate(sampleID3 = rownames(var))
datar16S <- left_join(datar16S, var, by = "sampleID3")
rownames(datar16S) <- datar16S[,1]

#Calculate Spearman coefficient between Shannon index and dimensions
#Example for Dim.1
res<-cor.test(datar16S$Dim.1, datar16S$Shannon, method ="spearman")
res

## scatter plot
ggplot(datar16S, aes(x = Dim.1, y = Shannon)) +
  geom_point() +
  geom_smooth(method = "lm", color = "#2c7fb8", fill = "#7fcdbb") +
  labs(x = "Dim.1", y = "Observed index") +
  theme_minimal()

#Calculate Spearman coefficient between Observed index and dimensions
```

```
#Example for Dim.1
res<-cor.test(datar16S$Dim.1, datar16S$Observed, method ="spearman")
res

## scatter plot
ggplot(datar16S, aes(x = Dim.1, y = Observed)) +
  geom_point() +
  geom_smooth(method = "lm", color = "#2c7fb8", fill = "#7fcdbb") +
  labs(x = "Dim.1", y = "Observed index") +
  theme_minimal()
```

***5-FAPROTAX*** # 5- FAPROTAX analysis to uncover association with mammal and human gut-associated bacteria: Figure S10A

```
#libraries
library(phyloseq)
library(microeco)
library("file2meco")

###########First part: prepare the dataset

#Transform the phyloseq object into microtable object
meco_dataset <- phyloseq2meco(r16S_L)

#prepare tables
t2 <- trans_func$new(meco_dataset)
t2$cal_spe_func(prok_database = "FAPROTAX")
t2$res_spe_func
t2$cal_spe_func_perc(abundance_weighted = FALSE)
t2$trans_spe_func_perc()
t2$plot_spe_func_perc()

#We want to know the number of OTU assigned to each function
func_table0 <- t2$res_spe_func

#Number of OTUs without any associated function in FAPROTAX
#and number of unensured functions
length(which(rowSums(func_table0) == 0))
length(which(colSums(func_table0) == 0))
func_table0 <- func_table0[-which(rowSums(func_table0) == 0), -which(colSums(func_table0) == 0)]
write.csv(func_table0, file = "Bacterial_functional_annotation_FAPROTAX_for_16S_zOTUs.csv")

length(which(rowSums(func_table0) == 0))
colSums(func_table0)
ASV_table<- as.data.frame(otu_table(r16S_L))

#Calculate the percentage of OTUs within a community that ensure the same function
#First, list the OTUs that are present in each site
list_sites_otu <- apply(ASV_table, 2, function(x) rownames(ASV_table)[which(x>0)])
#Create an empty table with functions in columns and sites in rows
df_func_site <- as.data.frame(matrix(0, ncol = ncol(func_table0), nrow = ncol(ASV_table), dimnames = li
#For each function and each site, find the number of OTUs present in the site
#that ensure the given function
```

```
for(i in 1:ncol(df_func_site)){
  for(j in 1:nrow(df_func_site)){
    df_ind_prim <- func_table0[which(func_table0[, colnames(df_func_site)[i]] >0),]
    df_ind1_prim <- df_ind_prim[which(rownames(df_ind_prim) %in% list_sites_otu[[j]]),]
    df_func_site[j,i] <- sum(ASV_table[rownames(ASV_table) %in% rownames(df_ind1_prim), j])/colSums(ASV
  }
}
func_table_perc_16S <- df_func_site

save(func_table_perc_16S, file = "Bacterial_functional_groups_16S_zOTUs.RData")

write.table(func_table_perc_16S, "/yourPath/new_func_table_perc.txt", sep=",")

write.csv(func_table_perc_16S,'new_func_table_perc.csv')

#############end of the first part################

#part 2- statistical analysis

library(car)
library(ggplot2)
library(ggpubr)
library(rstatix)
library(psych)

#import tables
functab <- read.table("FAPROTAX/new_func_table_perc.csv",
                      sep = ";",
                      dec = ".",
                      header = TRUE)

#transform to factor
functab$sampleID3<-as.factor(functab$sampleID3)
functab$genetic.distance<-as.factor(functab$genetic.distance)
functab$variety<-as.factor(functab$variety)
functab$block<-as.factor(functab$block)
functab$Seedling.cotyledon.shape<-as.factor(functab$Seedling.cotyledon.shape)
functab$Seedling.anthocyanin.presence<-as.factor(functab$Seedling.anthocyanin.presence)
functab$Leaf.shape.cultivated.material<-as.factor(functab$Leaf.shape.cultivated.material)
functab$Leaf.blistering<-as.factor(functab$Leaf.blistering)
functab$Leaf.margin.undulation<-as.factor(functab$Leaf.margin.undulation)
functab$Leaf.venation<-as.factor(functab$Leaf.venation)
functab$Leaf.division<-as.factor(functab$Leaf.division)
functab$Leaf.color<-as.factor(functab$Leaf.division)
functab$Leaf.color.intensity<-as.factor(functab$Leaf.color.intensity)
functab$Leaf.anthocyanin.content<-as.factor(functab$Leaf.anthocyanin.content)
functab$Side.shoot.formation.tendency<-as.factor(functab$Side.shoot.formation.tendency)
functab$Tipburn.sensitivity<-as.factor(functab$Tipburn.sensitivity)
functab$Plant.diameter<-as.factor(functab$Plant.diameter)
functab$Head.shape<-as.factor(functab$Head.shape)
functab$Head.leafs.overlap<-as.factor(functab$Head.leafs.overlap)
functab$Head.height<-as.factor(functab$Head.height)
functab$Heart.formation<-as.factor(functab$Heart.formation)
```

```r
#before doing dunn post hoc test check for normality to see if you can apply ANOVA. Our data were not n

#statistical tests
dunn_test(mammal_gut ~ Leaf.venation, data = functab)
#substitute mammal_gut with human_gut associated bacteria or human associated bacteria (or any other va
#results are reported in Figure S10B

#################end of the second part###############

#part 3: graphical representation

#Figure S10A

#mammal gut associated bacteria

gg_traits <- functab_filtered %>%
  ggplot(aes(x = Leaf.venation, y = mammal_gut)) +
  geom_violin(aes(fill = Leaf.venation), na.rm = TRUE, alpha = 0.2) +
  geom_boxplot(aes(fill = Leaf.venation), width = 0.1, na.rm = TRUE, fill = "white", color = "black") +
  geom_jitter(aes(fill = Leaf.venation, color = Leaf.venation), width = 0.2, size = 3, shape = 21, strol
  theme_classic() +
  scale_fill_manual(values = colors) +
  scale_color_manual(values = dark_colors) +
  guides(fill = FALSE)
gg_traits

#Human gut associated bacteria

gg_traits <- functab%>%
  ggplot(aes(x = Leaf.venation, y = human_gut)) +
  geom_violin(aes(fill = Leaf.venation), na.rm = TRUE, alpha = 0.2) +
  geom_boxplot(aes(fill = Leaf.venation), width = 0.1, na.rm = TRUE, fill = "white", color = "black") +
  geom_jitter(aes(fill = Leaf.venation, color = Leaf.venation), width = 0.2, size = 3, shape = 21, strol
  theme_classic() +
  scale_fill_manual(values = colors) +
  scale_color_manual(values = dark_colors) +
  guides(fill = FALSE)
gg_traits

#Human associated bacteria

gg_traits <- functab%>%
  ggplot(aes(x = Leaf.venation, y = human_associated)) +
  geom_violin(aes(fill = Leaf.venation), na.rm = TRUE, alpha = 0.2) +
  geom_boxplot(aes(fill = Leaf.venation), width = 0.1, na.rm = TRUE, fill = "white", color = "black") +
  geom_jitter(aes(fill = Leaf.venation, color = Leaf.venation), width = 0.2, size = 3, shape = 21, strol
  theme_classic() +
  scale_fill_manual(values = colors) +
  scale_color_manual(values = dark_colors) +
  guides(fill = FALSE)
gg_traits
```

***6-Log2Fold change analysis***

# 6- Log2Fold change analysis with Dseq2: Figures 7A and S10A

```
##part 1: prepare the dataset with phenotypic traits in binary form

#heart formation: groups 1-2 are considered "without the heart formation capability", group 7 is consid
sample_data(r16S_L)$Heart.formation_binary <- ifelse(
  is.na(sample_data(r16S_L)$Heart.formation),
  NA,
  ifelse(sample_data(r16S_L)$Heart.formation %in% c("1", "2"), "absent",
         ifelse(sample_data(r16S_L)$Heart.formation %in% c("7"), "present", NA))
)

#head height: plants in group 3 are considered short, in group 5 tall.
sample_data(r16S_L)$Head.height_binary <- ifelse(
  is.na(sample_data(r16S_L)$Head.height), # Check if Head.height is NA
  NA,
  ifelse(sample_data(r16S_L)$Head.height == "3", "short",
         ifelse(sample_data(r16S_L)$Head.height %in% c("5"), "high", NA))
)

#Leaf venation is already binary

##part 2: apply the Dseq2 function

library( "DESeq2" )
library(ggplot2)
library(tidyverse)
library(ggrepel)

#in the following script substitute "Heart.formation_binary" with the phenotypic trait you want to stud

#select the data in the binary format
r16S_L_selected= subset_samples(r16S_L, Heart.formation_binary != "NA")

#tranform as factor
sample_data(r16S_L_selected)$Heart.formation_binary<-as.factor(sample_data(r16S_L_selected)$Heart.forma

#apply sizeFactors(diagdds) function
diagdds = phyloseq_to_deseq2(r16S_L_selected, ~Heart.formation_binary)

diagdds = DESeq(diagdds, test="Wald", fitType="parametric")

res = results(diagdds, cooksCutoff = FALSE)

#to see the levels' order in the graph
head(res)

#set the p-value
alpha = 0.05
sigtab = res[which(res$pvalue < alpha), ]
sigtab = cbind(as(sigtab, "data.frame"), as(tax_table(r16S_L)[rownames(sigtab), ], "matrix"))

dimnames
```

```r
head(sigtab)
dim(sigtab)

##part 3: graphical representation. Figures 6A and S10A

t #set the theme
theme_set(theme_bw() +
          theme(panel.grid.major = element_blank(),
                panel.grid.minor = element_blank(),
                panel.border = element_rect(color = "black", fill = NA),
                axis.title.x = element_text(size = 12),
                axis.title.y = element_text(size = 12),
                axis.text.x = element_text(size = 7),
                axis.text.y = element_text(size = 7),
                legend.title = element_text(size = 10),
                legend.text = element_text(size = 8)))

# Reorder the Log2Foldchange values in the plot
sigtab <- sigtab[order(sigtab$log2FoldChange, decreasing = FALSE), ]
sigtab$ASV <- factor(sigtab$ASV, levels = sigtab$ASV)

#
gg <- ggplot(sigtab, aes(x = log2FoldChange, y = ASV, color = Order, size = baseMean)) +
  geom_point() +
  geom_vline(xintercept = 0, linetype = "dashed", color = "black") +
  scale_size_continuous(range = c(1, 10)) +
  scale_color_brewer(palette = "Set1") +
  labs(x = "log2 Fold Change", y = "Taxa", size = "Base Mean", color = "Order") +
  theme(axis.text.x = element_text(angle = 90, vjust = 0.5, hjust = 1, size = 7))
```

***7-Network analysis*** # 7- Network analysis: Figures 7B-C and S10B-C

```r
# Libraries
library(phyloseq)
library(tidyverse)
library(igraph)

#In the following script, substitute "Heart.formation_binary" with the other phenotypic traits you want

r16S_L_present= subset_samples(r16S_L, Heart.formation_binary == "present")
view(sample_data(r16S_L_present))

# Extract tax_table from phyloseq object
taxa_table <- as.data.frame(tax_table(r16S_L_present))
taxa_table <- tibble::rownames_to_column(taxa_table, var = "species")

# Extract metadata table from phyloseq object
metadata <- sample_data(r16S_L_present)
metadata <- as.data.frame(metadata)
metadata$rownames <- rownames(metadata)
metadata <- as_tibble(metadata) %>%
  column_to_rownames(var = "rownames")
```

```r
# Extract OTU table from phyloseq object and convert to relative abundance
otu_table <- as.data.frame(otu_table(r16S_L_present))

# Convert OTU table to relative abundance
sp_ratio <- otu_table %>%
  mutate(across(everything(), ~ ./sum(.))) %>%
  rownames_to_column(var = "species") %>%
  as_tibble()

# Display the first few rows of the sp_ratio
head(sp_ratio)

# Transform sp_ratio for network analysis
sp_ratio <- sp_ratio %>%
  as_tibble() %>%
  rename_all(tolower) %>%
  mutate(species = str_replace_all(species, "__", "")) %>%
  column_to_rownames(var = "species")

min.prevalence= 5
#for heart formation present: min.prevalence set at 6
#for heart formation absent: min.prevalence set at 6
#for head height short: min.prevalence set at 10
#for head height high: min.prevalence set at 4
#for leaf venation 1 : min.prevalence set at 12
#for leaf venation 2 : min.prevalence set at 5
incidence=sp_ratio
incidence[incidence>0]=1
sp_ratio_filtered <- sp_ratio[which(rowSums(incidence)>=min.prevalence),] ### end of prevalence filteri

sp_correl <- sp_ratio_filtered %>%
  t() %>%
  cor(method = "spearman")  ### correlation calculations

sp_correl[abs(sp_correl)<0.65]=0  ### define threshold for correlations

net_work <- graph_from_adjacency_matrix(sp_correl,mode="lower",weighted=TRUE, diag=FALSE)  ## create ne
net_work <- delete.vertices(net_work, degree(net_work)==0) #remove nodes without edges

plot(net_work, vertex.label = NA, edge.width = 5, vertex.size=10) ## first plot

###annotations

taxa_tbl <- taxa_table %>%
  as_tibble() %>%
  mutate(species = as.character(species),
         species = ifelse(is.na(species), "unknown", species),
         species = str_replace_all(species, "__", ""))  #tidy taxonomic infos

net_work_used <- V(net_work)$name %>%
   as_tibble() %>%
  mutate(species = value) %>%
  select(species) #extract species represented in the network
```

```r
v_attr <- sp_ratio %>% ### we create a table of attributes for nodes (vertex)
  rownames_to_column( var = "species") %>%
  as_tibble() %>%
  pivot_longer(-species, names_to = "sample_id", values_to = "ratio" ) %>%
  mutate(species = str_replace_all(species, "__", "")) %>%
  group_by(species) %>%
  summarise(rel_abundance = sum(ratio)) %>%
  inner_join(net_work_used, by = "species") %>%
  inner_join(taxa_tbl, by = "species") %>%
  mutate(rel_abundance = abs(exp (rel_abundance))) # we join taxonomic infos, relative abundance and sp


network_table <- igraph::as_data_frame(net_work, 'both') ##we convert the network in data frames

network_table$vertices <- network_table$vertices %>%
    as_tibble() %>%
    inner_join(v_attr, by = c("name"="species"))  # we add our attribute in the data frames

  net_work1 <- graph_from_data_frame(network_table$edges,
                                    directed = F,
                                    vertices = network_table$vertices) # we

################plot

mom_data <- V(net_work1)$Order %>%
  as_tibble_col(column_name = "species") #formating the class variable as factor (is needed for colorin
mom_data$species <- as_factor(mom_data$species)

color_easy <-  c("#FF0000", "#000080", "#32CD32", "#FFD700","#00FFFF", "#800080",  "#FF00FF", "#4682B4"

V(net_work1)$color <-  color_easy ## we have now the color attributes based on class

E(net_work1)$sign <- E(net_work1)$weight ##we create another attribute from the weight

E(net_work1)$weight <- abs(E(net_work1)$weight) ## we then use absolute value of weight because of the

######network plot: Figure 6B and S9B-C

plot(net_work1,
     vertex.size = 10,
     edge.width = abs(E(net_work1)$weight) * 2,
     vertex.label= NA,
     edge.color = ifelse(E(net_work1)$sign > 0, "blue", "red"),
     vertex.color = (net_work1)$Order,
     layout = layout_with_kk(net_work1))

title_legend1 <- V(net_work1)$Order %>%
  as_factor() %>%
  levels()

legend(x = 1, y = 1, title_legend1,
       pch = 21, pt.bg = c("#FF0000", "#000080", "#32CD32", "#FFD700","#00FFFF", "#800080",  "#FF00FF",
       pt.cex = 1.5, bty = "n", ncol = 1)
```

```
######hub ASVs plot: Figures 7C and S10B-C

#identify the hub score
hs <- hub_score(net_work1)$vector

#plot
plot(net_work1,
     vertex.size = hs*20,
     edge.width = abs(E(net_work1)$weight) * 2,
     vertex.label.cex= 0.6,
     edge.color = ifelse(E(net_work1)$sign > 0, "blue", "red"),
     vertex.color = (net_work1)$Order,
     layout = layout_with_kk(net_work1))

title_legend1 <- V(net_work1)$Order %>%
  as_factor() %>%
  levels()

legend(x = 1, y = 1, title_legend1,
       pch = 21, pt.bg = c("#FF0000", "#000080", "#32CD32", "#FFD700","#00FFFF", "#800080",  "#FF00FF",
       pt.cex = 1.5, bty = "n", ncol = 1)
```

***8- Varpart analysis*** # 8- Varpart analysis: Figure 5 and Tables S12-13

```
#library
library(vegan)

# Table S14- Create the model
mod <- varpart(bray.16S, ~genetic.distance, ~ variety,~ Dim.2+Dim.4+Dim.5,~ Heart.formation+Head.height
    data=mdat.16S_bray, transfo="hel")
mod
summary(mod)


#Table S14 - Redundancy analysis (RDA) model using Hellinger-transformed variables

#genetic distance
rda.result <- rda(decostand(bray.16S, "hell") ~ genetic.distance, data = mdat.16S_bray)
anova(rda.result)
#variety
rda.result <- rda(decostand(bray.16S, "hell") ~ variety, data = mdat.16S_bray)
anova(rda.result)
#mineral content
rda.result <- rda(decostand(bray.16S, "hell") ~ Dim.2+Dim.4+Dim.5, data = mdat.16S_bray)
anova(rda.result)
#phenotypic traits
rda.result <- rda(decostand(bray.16S, "hell") ~ Heart.formation+Head.height+Head.shape+Leaf.venation+Hea
    data=mdat.16S_bray)
anova(rda.result)

#Figure 5A- UpSetR plot
```

```r
library(UpSetR)

input <- c(
  "Genetic distance" = 3.5,
  "Variety" = 4.5,
  "Mineral content" = 2.4,
 "Phenotypic traits" = 6.5,
  "Variety&Genetic distance" = 7,
  "Genetic distance&Mineral content" = 5.8,
  "Genetic distance&Phenotypic traits" = 8.7,
  "Variety&Mineral content" = 6.8,
  "Variety&Phenotypic traits" = 9.6,
  "Mineral content&Phenotypic traits" = 8.6,
  "Mineral content&Genetic distance&Variety" = 9.2,
  "Genetic distance&Variety&Phenotypic traits" = 11.8,
  "Genetic distance&Mineral content&Phenotypic traits" = 10.9,
 "Variety&Mineral content&Phenotypic traits"= 11.7,
 "Genetic distance&Variety&Mineral content&Phenotypic traits"=13.9)


names_list <- sub("=.*", "", names(input))
numeric_values <- as.numeric(input)

# Combine names and values
sorted_data <- data.frame(names = names_list, values = numeric_values)
sorted_data <- sorted_data[order(-sorted_data$values), ]

# Create a bar plot using ggplot2
ggplot(sorted_data, aes(x = factor(names, levels = sorted_data$names), y = values)) +
  geom_bar(stat = "identity", fill = "gray23", width = 0.7) +
  labs(title = "Total variance explained by each factor", x = "Category", y = "% of variation") +
  theme(axis.text.x = element_text(angle = 45, hjust = 1),
        panel.border = element_blank(),  # Remove border around the image
        panel.grid.major = element_blank(),  # Remove major grid lines
        panel.grid.minor = element_blank(),  # Remove minor grid lines
        panel.background = element_blank(),  # Remove gray background
        plot.background = element_blank(),   # Remove plot background
        axis.line = element_line(color = "black")) +
  geom_text(aes(label = values), vjust = -0.5, size = 3)

library(UpSetR)
set_order <- c("Phenotypic traits", "Variety", "Genetic distance", "Mineralcontent")

upset<-upset(fromExpression(input),
      nintersects = 15,
      nsets = 15,
      order.by = "freq",
      decreasing = T,
      mb.ratio = c(0.6, 0.4),
      number.angles = 0,
      text.scale = 1.5,
      point.size = 2.8,
      line.size = 1
```

```
      )
upset
```

***9-HEATMAP*** # 9- Figure S11 -heatmap

```
#Creation of an heatmap with differential relative abundance between the different heart formation stag

gpt <- subset_taxa(r16S_L, Kingdom == "d__Bacteria")

# Etract OTU matrix and taxonomical data
otu_table_df <- as.data.frame(otu_table(gpt))
tax_table_df <- as.data.frame(tax_table(gpt))

#Be sure rownames are the same
otu_table_df$ASV <- rownames(otu_table_df)
tax_table_df$ASV <- rownames(tax_table_df)

# join OTU table with taxa table
otu_taxa_df <- merge(otu_table_df, tax_table_df, by = "ASV")
otu_taxa_df <- otu_taxa_df[, -c(224:226)] #delete eventual columns like "confidence", "interval", "king

# Convert data in long format
library(tidyr)
#as character
otu_taxa_df[] <- lapply(otu_taxa_df, function(x) if(is.numeric(x)) as.character(x) else x)

otu_table_long <- pivot_longer(
  otu_taxa_df,
  cols = -c(ASV, Phylum, Class, Order, Family, Genus, Species),
  names_to = "SampleID",
  values_to = "Abundance"
)

# Add SampleID colum in metadata
metadata_df <- as.data.frame(sample_data(gpt))
metadata_df$SampleID <- rownames(metadata_df)

# Debugging: check if it works
print(names(metadata_df))
head(metadata_df)

# add metadata to the long-format table
add_metadata <- function(otu_table_long, metadata) {
  metadata_df <- metadata
  metadata_df <- metadata_df[, c("SampleID", "Heart.formation")]

  # function to add metadata
  otu_table_long$Heart.formation <- NA
  for (i in seq_len(nrow(otu_table_long))) {
    sample_id <- otu_table_long$SampleID[i]
    if (sample_id %in% metadata_df$SampleID) {
      otu_table_long$Heart.formation[i] <- metadata_df$Heart.formation[metadata_df$SampleID == sample_i
    }
```

```r
  }

  return(otu_table_long)
}


# apply function
otu_table_long <- add_metadata(otu_table_long, metadata_df)
otu_table_long$Family<-as.factor(otu_table_long$Family)
otu_table_long$Heart.formation<-as.factor(otu_table_long$Heart.formation)
otu_table_long$Abundance<-as.numeric(otu_table_long$Abundance)

otu_table_long %>%
  filter(!is.na(Heart.formation)) %>%
  droplevels()

#Select only relevant columns
otu_table_long_1 <- otu_table_long[, c(5,9, 10)]

otu_table_long_1 %>%
  group_by(Family, Heart.formation) %>%
  summarise (Abundance= mean(Abundance)) -> otu_table_long_1

#Transform to wide
otu_table_long_1_wide <- otu_table_long_1 %>%
  pivot_wider(
    names_from = Heart.formation,
    values_from = Abundance,
    names_vary = "slowest"
  )

#Convert to data frame
otu_table_long_1_wide <- as.data.frame(otu_table_long_1_wide)

#Remove NA in ASV column
otu_table_long_1_wide <- na.omit(otu_table_long_1_wide)

#Set rownames
row.names(otu_table_long_1_wide) <- otu_table_long_1_wide[, 1]

#Remove col1
otu_table_long_1_wide <- otu_table_long_1_wide[, -1]

#Convert to matrix
otu_table_long_1_wide_matrix <- as.matrix(otu_table_long_1_wide)

#Quantile breakes function
quantile_breaks <- function(xs, n = 10) {
  xs <- na.omit(xs)
  zero_included <- 0
  positive_values <- xs[xs > 0]
  quantiles <- quantile(positive_values, probs = seq(0, 1, length.out = n - 1), na.rm = TRUE)
  breaks <- unique(c(zero_included, quantiles))
```

```
  breaks <- sort(breaks)
  return(breaks)
}
mat_breaks <- quantile_breaks(otu_table_long_1_wide_matrix, n = 10)


#pheatmap: Figure S12
pheatmap(
  mat               = otu_table_long_1_wide_matrix,
  color             = colorRampPalette(c("#f7fcf0", "#e0f3db","#ccebc5", "#a8ddb5", "#7bccc4", "#4eb3d3
 breaks            = mat_breaks,
  border_color      = NA,
  show_colnames     = TRUE,
  show_rownames     = TRUE,
  cluster_cols = F,
 cellwidth         = 30,
 # annotation_col    = mat_col,
  #annotation_colors = mat_colors,
  drop_levels       = TRUE,
  fontsize          = 10,
 fontsize_row      = 2,
  fontsize_col      = 10,
 cutree_rows = 2,
  main              = "Family relative abundance across heart formation capacities"
)
```

***10-FEAST***

# 10- FEAST analyis, unravelling origins: Figure S3

### 10.1- Dataset preparation

```
#To do this part we used the 1) leaf dataset with 2) no rarefaction and 3) no Abundance threshold.

Denoising <- read.csv("Output_qiime_qzv/Denoising.tsv", header=TRUE, sep = "\t",  dec=".")

Denoising

#Download packages

library("phyloseq")
library("ggplot2")
library("readxl")
library("dplyr")
library("tibble")
library("tidyverse")
library("magrittr")


#Upload tables
##ASV Table
```

```
asv16S <- read.table(file="yourPath/16S_table-FINAL.txt",sep="\t",dec = ".",header=TRUE)
##Tax Table
tax16S <- read.table(file="yourPath/taxonomy.tsv",sep="\t",dec = ".",header=TRUE)
##Design
  design <- read.table(file="yourPath/Design.txt",sep="\t",dec = ",",header=TRUE)

##Sequences
library(Biostrings)
refseq16S <- readDNAStringSet("yourPath/dna-sequences.fasta")

#Manage data for Phyloseq
asv16S <- asv16S %>%
    tibble::column_to_rownames("OTU.ID")
tax16S <- tax16S %>%
    tibble::column_to_rownames("OTU.ID")

#Collapse Tax Table
library(stringr)
tax16S[c("Kingdom","Phylum", "Class", "Order", "Family", "Genus", "Species")] <- str_split_fixed(tax16S
tax16S[tax16S == ""] <- NA
tax16S[tax16S == " "] <- NA
design <- design %>%
    tibble::column_to_rownames("sampleID")

otu_mat <- as.matrix(asv16S)
tax_mat <- as.matrix(tax16S)

#Create Phyloseq Object  #phylogenetic info to be added
ps16S_0 <- phyloseq(tax_table(tax_mat),
                  otu_table(otu_mat, taxa_are_rows = TRUE), sample_data(design), refseq(refseq16S))

#TAXA
# Calculate the number of taxa before filtering
num_taxa_before <- ntaxa(ps16S_0)


#Filtered ASV based on taxonomy
ps16S_1a <- subset_taxa(ps16S_0, !is.na(Taxon) & !Taxon %in% c("Unassigned"))
ps16S_1b <- subset_taxa(ps16S_1a, !Order %in% c(" o__Chloroplast"))
ps16S_1c<- subset_taxa(ps16S_1b, !Order %in% c("o__Mitochondria"))
ps16S_1d<- subset_taxa(ps16S_1c, !Family %in% c("f__Mitochondria"))
ps16S_1e<- subset_taxa(ps16S_1d, !Family %in% c("f__Chloroplast"))
ps16S_1f<- subset_taxa(ps16S_1e, !Genus %in% c(" g__Mitochondria"))
ps16S_1g<-subset_taxa(ps16S_1f, !Taxon %in% c("d__Eukaryota"))
ps16S_1h<-subset_taxa(ps16S_1g, !Kingdom %in% c("d__Eukaryota"))
ps16S_1i<-subset_taxa(ps16S_1h, !Kingdom %in% c("d__Archaea"))

##decontamination from blank control samples
#libraries
library(here); packageVersion("here")
library(decontam); packageVersion("decontam")
library(phyloseq); packageVersion("phyloseq")
library(Biostrings); packageVersion("Biostrings")
```

```r
library(tidyverse); packageVersion("tidyverse")

d16S_leaves <- subset_samples(ps16S_1i, tissue %in% c("leaf"))
d16S_leaves <- subset_taxa(d16S_leaves, !taxa_sums(d16S_leaves) == 0)
d16S_soil <- subset_samples(ps16S_1i, tissue %in% c("soil"))
d16S_soil <- subset_taxa(d16S_soil, !taxa_sums(d16S_soil) == 0)
d16S_endophytes <- subset_samples(ps16S_1i, tissue %in% c("endophytes"))
d16S_endophytes <- subset_taxa(d16S_endophytes, !taxa_sums(d16S_endophytes) == 0)
d16S_seeds <- subset_samples(ps16S_1i, tissue %in% c("seed"))
d16S_seeds <- subset_taxa(d16S_seeds, !taxa_sums(d16S_seeds) == 0)

#import clean phyloseq from blanks
phyloseq_bianchi <- readRDS("ps_blanks.rds")

#prepare dataset
ps16S_1i.2<- merge_phyloseq(d16S_leaves, phyloseq_bianchi)
sample_data(ps16S_1i.2)$FEAST[394:400] <- paste("blank")
base::as.data.frame(phyloseq::sample_data(ps16S_1i.2))
base::as.data.frame(phyloseq::sample_data(d16S_leaves))
view(sample_data(ps16S_1i.2))

#inspect libraries sizes

library("gtable")

df <- as.data.frame(sample_data(ps16S_1i.2))
df$LibrarySize <- sample_sums(ps16S_1i.2)
df <- df[order(df$LibrarySize),]
df$Index <- seq(nrow(df))
df$FEAST<-as.factor(df$FEAST)
ggplot(data=df, aes(x=Index, y=LibrarySize, color=FEAST)) + geom_point()+
  theme(
    legend.title = element_text(size = 5),
    legend.text = element_text(size = 5),
    legend.key.size = unit(0.5, "cm"),
    legend.key.width = unit(0.5, "cm")
  )

#Prevalence based-decontamination
 sample_data(ps16S_1i.2)$is.neg <- sample_data(ps16S_1i.2)$FEAST == "blank"
contamdf.prev <- isContaminant(ps16S_1i.2, method="prevalence", neg="is.neg")
table(contamdf.prev$contaminant)

# Make phyloseq object of presence-absence in negative controls and true samples
ps.pa <- transform_sample_counts(ps16S_1i.2, function(abund) 1*(abund>0))
ps.pa.neg <- prune_samples(sample_data(ps.pa)$FEAST == "blank", ps.pa)
ps.pa.pos <- prune_samples(sample_data(ps.pa)$FEAST == "leaf", ps.pa)
# Make data.frame of prevalence in positive and negative samples
df.pa <- data.frame(pa.pos=taxa_sums(ps.pa.pos), pa.neg=taxa_sums(ps.pa.neg),
                      contaminant=contamdf.prev$contaminant)
ggplot(data=df.pa, aes(x=pa.neg, y=pa.pos, color=contaminant)) + geom_point() +
  xlab("Prevalence (Negative Controls)") + ylab("Prevalence (True Samples)")
```

```
#trim contaminants

ps.noncontam <- prune_taxa(!contamdf.prev$contaminant, ps16S_1i.2)
ps.noncontam

ps16S_1i.5<- merge_phyloseq(ps.noncontam, d16S_endophytes,d16S_seeds, d16S_soil)
view(otu_table(ps16S_1i.5))

ps16S_1i.5= subset_samples(ps16S_1i.5, FEAST != "blank")

#remove samples with less than 100 reads
ps16S_1i.4 <- prune_samples(sample_sums(ps16S_1i.5)>=100, ps16S_1i.5)#Before
ps16S_1i.5 <- filter_taxa(ps16S_1i.4, function(x) sum(x) > 0, TRUE)
view(sample_data(ps16S_1i.5))
```

### 10.2- One sink: Table S10 B

```
Packages <- c("Rcpp", "RcppArmadillo", "vegan", "dplyr", "reshape2", "gridExtra", "ggplot2", "ggthemes"
lapply(Packages, library, character.only = TRUE)
library(FEAST)

FEAST_dataset = merge_samples(ps16S_1i.5, "FEAST")
otu_table<- as.matrix(t(otu_table(FEAST_dataset)))
write.csv(otu_table, "otu_table.csv")

#upload the metadata table
metadata <- Load_metadata(metadata_path = "C:/yourPath/metadata.txt")

#upload the otu table
otus <- Load_CountMatrix(CountMatrix_path = "C:/yourPath/otu_table.txt")

#This output is reported in Table S10 B
FEAST_output <- FEAST(C = otus, metadata = metadata, EM_iterations = 1000, different_sources_flag = 1,
```

### 10.3- Multiple sinks: Table S10 A and Figure S3A

```
Packages <- c("Rcpp", "RcppArmadillo", "vegan", "dplyr", "reshape2", "gridExtra", "ggplot2", "ggthemes"
lapply(Packages, library, character.only = TRUE)
library(FEAST)

FEAST_dataset = merge_samples(ps16S_1i.5, "FEAST2")

sample_data<- as.matrix(sample_data(FEAST_dataset))
otu_table<- as.matrix(otu_table(t(FEAST_dataset)))
write.csv(otu_table, "otu_table_byblocks.csv")
write.csv(sample_data, "sample_byblocks.csv")

metadata <- Load_metadata(metadata_path = "C:/yourPath/metadata.txt")
```

```r
otus <- Load_CountMatrix(CountMatrix_path = "C:/yourPath/otu_table_byblocks.txt")
#otus <- otus[-c(1, 2), ]

#This output is reported in Table S10 A
FEAST_output <- FEAST(C = otus, metadata = metadata,  EM_iterations = 10000, different_sources_flag = 1


################################boxplot##############################
#in this case the FEAST function have to be applied for every sample. Since tables are large, there is 

#metadata preparation

#extract samples in your metadata table
sample_data<- as.matrix(sample_data(ps16S_1i.5))

# Use samples to create the following vector
colonna_F <- c("01A", "02A", "03A", "04A", "06A", "07A", "08A", "09A", "11A", "13A",
  "14A", "16A", "17A", "19A", "20A", "21A", "22A", "23A", "24A", "25A",
  "26A", "27A", "28A", "29A", "30A", "31A", "32A", "33A", "34A", "35A",
  "36A", "37A", "38A", "39A", "40A", "41A", "42A", "43A", "44A", "45A",
  "46A", "47A", "48A", "49A", "50A", "51A", "53A", "54A", "55A", "56A",
  "57A", "58A", "59A", "62A", "63A", "64A", "65A", "66A", "67A", "68A",
  "69A", "70A", "71A", "72A", "73A", "74A", "75A", "76A", "77A", "78A",
  "79A", "80A", "81A", "82A", "83A", "84A", "85A", "86A", "87A", "88A",
  "89A", "90A", "91A", "92A", "93A", "94A", "95A", "96A", "97A", "98A",
  "99A", "100A", "101A", "102A", "103A", "104A", "105A", "106A", "107A",
  "108A", "109A", "110A", "111A", "112A", "113A", "114A", "115A", "116A",
  "117A", "118A", "119A", "120A", "121A", "122A", "123A", "124A", "125A",
  "126A", "127A", "128A", "129A", "130A", "131A", "132A", "134A", "135A",
  "137A", "138A", "139A", "495A", "01B", "02B", "03B", "04B", "06B",
  "07B", "08B", "09B", "11B", "13B", "14B", "16B", "17B", "19B", "20B",
  "21B", "22B", "23B", "24B", "25B", "26B", "27B", "28B", "29B", "30B",
  "31B", "32B", "33B", "34B", "35B", "36B", "37B", "38B", "39B", "40B",
  "41B", "42B", "43B", "44B", "45B", "46B", "47B", "48B", "49B", "50B",
  "51B", "53B", "54B", "55B", "56B", "57B", "58B", "59B", "62B", "63B",
  "64B", "65B", "66B", "67B", "68B", "69B", "70B", "71B", "72B", "73B",
  "74B", "75B", "76B", "77B", "78B", "79B", "80B", "81B", "82B", "83B",
  "84B", "85B", "86B", "87B", "88B", "89B", "90B", "91B", "92B", "93B",
  "94B", "95B", "96B", "97B", "98B", "99B", "101B", "102B", "103B",
  "104B", "105B", "106B", "107B", "108B", "109B", "110B", "111B", "112B",
  "113B", "115B", "116B", "117B", "118B", "119B", "120B", "121B", "122B",
  "123B", "124B", "125B", "126B", "127B", "128B", "129B", "130B", "131B",
  "132B", "134B", "135B", "136B", "137B", "138B", "139B", "495B", "01C",
  "02C", "03C", "04C", "06C", "07C", "08C", "09C", "11C", "14C", "16C",
  "19C", "20C", "21C", "22C", "23C", "24C", "25C", "26C", "27C", "28C",
  "29C", "30C", "31C", "32C", "33C", "34C", "35C", "36C", "37C", "38C",
  "39C", "40C", "41C", "42C", "43C", "44C", "45C", "46C", "47C", "48C",
  "49C", "50C", "51C", "53C", "54C", "55C", "56C", "57C", "58C", "59C",
  "62C", "63C", "64C", "65C", "66C", "67C", "68C", "69C", "70C", "71C",
  "72C", "73C", "74C", "75C", "76C", "77C", "78C", "79C", "80C", "81C",
  "82C", "83C", "84C", "85C", "86C", "87C", "88C", "89C", "90C", "91C",
  "92C", "93C", "94C", "95C", "96C", "97C", "98C", "99C", "100C", "101C",
  "102C", "103C", "104C", "105C", "106C", "107C", "108C", "109C", "110C",
```

```r
  "111C", "112C", "113C", "114C", "115C", "116C", "118C", "119C", "120C",
  "121C", "122C", "123C", "124C", "125C", "126C", "127C", "128C", "129C",
  "130C", "131C", "132C", "134C", "135C", "136C", "137C", "138C", "139C",
  "495C")

# Function to generate the table
generate_table <- function(env_list) {
  sampleID <- c()
  Env <- c()
  SourceSink <- c()
  id <- c()

  # ID counter
  id_counter <- 1

  for (env in env_list) {
    # Sink insertion
    sampleID <- c(sampleID, paste0("A", id_counter))
    Env <- c(Env, env)
    SourceSink <- c(SourceSink, "Sink")
    id <- c(id, id_counter)

    #Source insertion
    for (source in c("soil 1", "soil 2", "soil 3", "seed", "endophytes")) {
      sampleID <- c(sampleID, paste0("A", id_counter + 1))
      Env <- c(Env, source)
      SourceSink <- c(SourceSink, "Source")
      id <- c(id, id_counter)

      # To increase the ID row by row
      id_counter <- id_counter
    }

    id_counter <- id_counter + 1
  }

  df <- data.frame(sampleID, Env, SourceSink, id, stringsAsFactors = FALSE)
  return(df)
}

#Create a data frame with our sample names
df <- generate_table(colonna_F)

# Modify ID to have an increase of 6 columns after every sample
df$id <- rep(seq(1, ceiling(nrow(df) / 6)), each = 6, length.out = nrow(df))

# Save the dataframe as CSV
write.csv(df, "tabella_generata.csv", row.names = FALSE)

###otu_table

library(dplyr)
library(tidyr)
```

```r
#otu_table for sink samples
otu_table<- as.matrix(otu_table(ps16S_1i.5))
write.csv(otu_table, "otu_table_byblocks.csv")

#otu_table for source samples
otu_sources = merge_samples(ps16S_1i.5, "FEAST")
otu_table_sources<- as.matrix(t(otu_table(otu_sources)))
write.csv(otu_table_sources, "otu_table_sources.csv")

#now the otu of source samples have to be inserted after the otu of each sink samples. The following sc

# Read the .txt files
otu_table_byblocks <- read.table(file.path(file_path, "otu_table_byblocks.txt"), header = TRUE, sep = "

# Set the first column as row names for both tables
otu_table_byblocks <- otu_table_byblocks %>%
  column_to_rownames(var = names(otu_table_byblocks)[1])

# Function to insert empty columns
insert_empty_columns <- function(df, n) {
  df <- as.data.frame(df)
  # Create an empty list to hold the columns
  expanded_list <- list()

  # Iterate through each column in the original data frame
  for (i in 1:ncol(df)) {
    expanded_list[[length(expanded_list) + 1]] <- df[[i]]
    if (i < ncol(df)) { # Add empty columns only if it's not the last column
      expanded_list <- c(expanded_list, replicate(n, NA, simplify = FALSE))
    }
  }

  # Combine the list into a new data frame
  expanded_df <- do.call(cbind, expanded_list)
  return(expanded_df)
}

# Create a copy of the blocks table and add empty columns
otu_table_expanded <- insert_empty_columns(otu_table_byblocks, 5)

write.csv(otu_table_expanded, "otu_table_expanded.csv")

#now insert the sources'otus after the otus of each sample

##########################apply function


metadata <- Load_metadata(metadata_path = "C:/yourPath/metadata.txt")

otus <- Load_CountMatrix(CountMatrix_path = "C:/yourPath/otu_table.txt")

FEAST_output <- FEAST(C = otus, metadata = metadata,  EM_iterations = 10000, different_sources_flag = 1
```

```
##IMPORT OUTPUT
  FEAST_output <- read.table(file="C:/yourPath/demo_prova_source_contributions_matrix.txt",sep="\t",dec

FEAST_output<-as.data.frame(FEAST_output)

write.csv(FEAST_output, "FEAST_output.csv")


#script to delete the NA from the FEAST_output file

shift_left <- function(df) {
  t(apply(df, 1, function(row) {
    non_na_values <- row[!is.na(row)]
    c(non_na_values, rep(NA, length(row) - length(non_na_values)))
  }))
}


df_shifted <- as.data.frame(shift_left(FEAST_output))

write.csv(df_shifted, "df_shifted.csv", row.names = FALSE, na = "")
#join this output with the metadata table

####graphical representation

#import your new metadata table
Origin_dataset <- read.table(file="C:/yourPath/sample_byblocks.txt" ,sep="\t",dec = ",",header=TRUE)

Origin_dataset$A2_soil.1<-as.numeric(Origin_dataset$A2_soil.1)
Origin_dataset$A3_soil.2<-as.numeric(Origin_dataset$A3_soil.2)
Origin_dataset$A4_soil.3<-as.numeric(Origin_dataset$A4_soil.3)
Origin_dataset$A5_seed<-as.numeric(Origin_dataset$A5_seed)
Origin_dataset$A6_endophytes<-as.numeric(Origin_dataset$A6_endophytes)
Origin_dataset$Unknown<-as.numeric(Origin_dataset$Unknown)
Origin_dataset$genotype2<-as.factor(Origin_dataset$genotype2)
Origin_dataset$variety<-as.factor(Origin_dataset$variety)
Origin_dataset$block<-as.factor(Origin_dataset$block)
Origin_dataset$genetic.distance<-as.factor(Origin_dataset$genetic.distance)
Origin_dataset$sampleID3<-as.factor(Origin_dataset$sampleID3)

#remove Na in "genetic distance" column
Origin_dataset <- Origin_dataset %>%
  filter(!is.na(genetic.distance) & genetic.distance != "" & genetic.distance != "Na") %>%
  droplevels()

# Reshape data
library(reshape2)

dati_long <- melt(Origin_dataset, id.vars = c("sampleID3", "variety", "genetic.distance", "block"), mea

dati_long$value<-as.numeric(dati_long$value)
dati_long$variable<-as.factor(dati_long$variable)
dati_long$value <- round(dati_long$value, 2)
```

```
dati_long$log_value <- log(dati_long$value)

#Figure S4A
#by block
# Imposta l'ordine desiderato per 'variable'
ordine_variabili <- c("A5_seed", "A4_soil.3", "A2_soil.1", "A3_soil.2", "A6_endophytes")
dati_long$variable <- factor(dati_long$variable, levels = ordine_variabili)

block_colors <- c(
  "A" = "#fcbba1",
  "B" = "#fa9fb5",
  "C" = "#c51b8a"
)

#boxplot
gg1 <- ggplot(dati_long, aes(x = variable, y = value, color=block)) +
  geom_boxplot() +
  geom_jitter(aes(color = block), size = 1,  position = position_jitterdodge(jitter.width = 0.3, dodge.
  labs(x = "Sources", y = "Average source contribution") +
  facet_wrap(~ variable, scales = "free_x", ncol=5) +
  scale_color_manual(values = block_colors) +
  theme_classic() +
  ggtitle("Average source contribution") +
  theme(axis.text.x = element_text(angle = 90, vjust = 0.5, hjust = 1),
        strip.text = element_text(size = 12),
        legend.position = "bottom",
        panel.spacing = unit(1, "lines"))
```

#10.4- Fig. S9- Leaf metagenome-assembled genomes are differentially enriched among shoot phenotypic traits

```
###############################################################
#   Analysis of MAG-associated Shannon Diversity Across Traits
###############################################################

# Load required packages
library(rstatix)
library(ggpubr)
library(dplyr)
library(picante)
library(vegan)

###############################################################
# 1. Data Import and Preprocessing
###############################################################

# Import the MAG table (TPM + phenotypic traits; MICE-imputed)
data_MAGs <- read.delim(
  "C:/your path/table_mags_.tsv", #Table S16
  header = TRUE, sep = "\t"
)

# Ensure correct variable types
```

```r
data_MAGs$Sample          <- as.factor(data_MAGs$Sample)
data_MAGs$MAG             <- as.factor(data_MAGs$MAG)
data_MAGs$Heart.formation <- as.factor(data_MAGs$Heart.formation)
data_MAGs$Head.height     <- as.factor(data_MAGs$Head.height)
data_MAGs$Head.shape      <- as.factor(data_MAGs$Head.shape)
data_MAGs$Leaf.venation   <- as.factor(data_MAGs$Leaf.venation)

# Remove missing trait values
data_MAGs <- data_MAGs %>%
  filter(Heart.formation != "") %>%
  droplevels()

# Remove MAGs with zero relative abundance
data_MAGs <- data_MAGs %>%
  filter(Relative.Abundance != "0") %>%
  droplevels()

################################################################
# 2. Shannon Diversity per Sample
################################################################

Shannon_values <- data_MAGs %>%
  group_by(Sample) %>%
  summarise(Shannon = diversity(Relative.Abundance, index = "shannon"))

# Merge Shannon diversity back into the dataset
data_MAGs <- left_join(data_MAGs, Shannon_values, by = "Sample")

################################################################
# 3. Trait-Based Statistical Testing and Visualization
################################################################
#   For each trait:
#   - Dunn test with FDR correction
#   - Custom color palette
#   - Boxplot + jitter, publication-quality aesthetics
################################################################

################################################################
# A. Heart Formation
################################################################

# Dunn test
res <- data_MAGs %>%
  dunn_test(Shannon ~ Heart.formation, p.adjust.method = "fdr")

# Coordinates for plotting significant comparisons
stat.test <- res %>%
  filter(p.adj < 0.05) %>%
  add_xy_position(x = "Heart.formation")

# Color palette
Heart.palette <- c("#ffffe5", "#f7fcb9", "#d9f0a3", "#addd8e",
                   "#78c679", "#41ab5d", "#238443", "#006837", "#004529")
```

```r
# Plot
gg_traits <- data_MAGs %>%
  ggplot(aes(x = Heart.formation, y = Shannon, fill = Heart.formation)) +
  geom_boxplot(na.rm = TRUE) +
  geom_jitter(na.rm = TRUE, size = 1) +
  theme_classic() +
  scale_fill_manual(values = Heart.palette) +
  labs(y = "Shannon Diversity Index", x = "Heart formation")
gg_traits

################################################################
# B. Head Height
################################################################

# Dunn test
res <- data_MAGs %>%
  dunn_test(Shannon ~ Head.height, p.adjust.method = "fdr")

# Significant comparisons
stat.test <- res %>%
  filter(p.adj < 0.05) %>%
  add_xy_position(x = "Head.height")

# Color palette
Height.palette <- c("#ffffd4", "#fee391", "#fec44f",
                    "#fe9929", "#d95f0e", "#993404")

# Plot
gg_traits <- data_MAGs %>%
  ggplot(aes(x = Head.height, y = Shannon, fill = Head.height)) +
  geom_boxplot(na.rm = TRUE) +
  geom_jitter(na.rm = TRUE, size = 1) +
  theme_classic() +
  scale_fill_manual(values = Height.palette) +
  labs(y = "Shannon Diversity Index", x = "Head height")
gg_traits

################################################################
# C. Leaf Venation
################################################################

# Dunn test
res <- data_MAGs %>%
  dunn_test(Shannon ~ Leaf.venation, p.adjust.method = "fdr")

# Significant comparisons
stat.test <- res %>%
  filter(p.adj < 0.05) %>%
  add_xy_position(x = "Leaf.venation")

# Color palette
venation.palette <- c("#d8b365", "#5ab4ac")
```

```
# Plot
gg_traits <- data_MAGs %>%
  ggplot(aes(x = Leaf.venation, y = Shannon, fill = Leaf.venation)) +
  geom_boxplot(na.rm = TRUE) +
  geom_jitter(na.rm = TRUE, size = 1) +
  theme_classic() +
  scale_fill_manual(values = venation.palette) +
  labs(y = "Shannon Diversity Index", x = "Leaf venation")
gg_traits
```
